# Identification of *Drosophila* Gene Products Required for Phagocytosis of *Leishmania donovani*

**DOI:** 10.1371/journal.pone.0051831

**Published:** 2012-12-13

**Authors:** Adam Peltan, Laura Briggs, Gareth Matthews, Sean T. Sweeney, Deborah F. Smith

**Affiliations:** 1 Centre for Immunology and Infection, University of York, York, United Kingdom; 2 Department of Biology, Hull-York Medical School, University of York, York, United Kingdom; Louisiana State University, United States of America

## Abstract

The identity and function of host factors required for efficient phagocytosis and intracellular maintenance of the protozoan parasite *Leishmania donovani* are poorly understood. Utilising the phagocytic capability of *Drosophila* S2 cells, together with available tools for modulating gene expression by RNAi, we have developed an experimental system in which to identify host proteins of this type on a genome-wide scale. We have shown that *L. donovani* amastigotes can be phagocytosed by S2 cells, in which they replicate and are maintained in a compartment with features characteristic of mammalian phagolysosomes. Screening with dsRNAs from 1920 conserved metazoan genes has identified transcripts that, when reduced in expression, cause either increased or decreased phagocytosis. Focussing on genes in the latter class, RNAi-mediated knockdown of the small GTPase Rab5, the prenylated SNARE protein YKT6, one sub-unit of serine palmitoyltransferase (*spt2/lace),* the Rac1-associated protein Sra1 and the actin cytoskeleton regulatory protein, SCAR, all lead to a significant reduction in parasite phagocytosis. A role for the *lace* mammalian homologue in amastigote uptake by mammalian macrophages has been verified using the serine palmitoyltransferase inhibitor, myriocin. These observations suggest that this experimental approach has the potential to identify a large number of host effectors required for efficient parasite uptake and maintenance.

## Introduction

*Leishmania* are single celled parasitic protozoa, transmitted by the bite of female phlebotomine sand flies and responsible for a spectrum of human and animal diseases, collectively termed the Leishmaniases. Visceral leishmaniasis (VL, ‘Kala-azar’) is the most serious form of leishmaniasis (caused by species including *L. donovani* and *L. infantum*), and involves the migration of parasites from the site of infection to internal viscera such as the liver, spleen and bone marrow [1]. Patients present with hepatomegaly, splenomegaly, fever, pain and cachexia [2] and the disease is almost always fatal if untreated [3]. *Leishmania* species are found in 98 countries worldwide, including the tropics, subtropics and southern Europe [4] (http://www.dndi.org/diseases/vl.html). It is estimated that 350 million people are at risk from these diseases, with at least 90,000 cases of VL (visceral leishmaniasis) annually and 300,000 cases of CL (cutaneous leishmaniasis) [5] although these figures may underestimate the disease burden due to under-reporting (http://www.dndi.org/diseases/vl.html).

*Leishmania* parasites undergo a digenetic life cycle with both insect and mammalian hosts. Upon inoculation into the mammalian host by the sand fly, metacyclic promastigotes are rapidly engulfed by mononuclear phagocytes including macrophages [6], dendritic cells [7] and neutrophils [8]. Once internalised, promastigotes develop into macrophage-adapted aflagellated amastigotes within a membrane bound organelle termed the phagosome which matures into a phagolysosome by fusion events with endocytic organelles. *Leishmania* amastigotes are able to replicate within a fully mature phagolysosome, and infection is thought to be spread either by rupture of host macrophages and release of amastigotes, or by a process related to exocytosis [9]. During the next blood meal, amastigote-containing macrophages are taken up by the sand fly in which they differentiate into procyclic promastigotes to continue the transmission cycle.

The release of amastigotes and subsequent infection of new host cells is essential for disease progression, but poorly understood mechanistically, with most research to date focussing on the entry of promastigotes into macrophages. Both promastigotes and amastigotes enter host cells by host-mediated phagocytosis [10], with *L. donovani* amastigotes entering cells at a higher rate and eliciting a smaller respiratory burst than stationary phase (metacyclic enriched) promastigotes [11]. Amastigotes can enter by a variety of receptors including CR3 and FcγR [12] but other pathways may also be utilised depending on the opsonisation state of the parasites. Thus hamster spleen derived *L. donovani* amastigotes enter RAW264.7 macrophages via a FcγRII/III, RhoA and Cdc42 independent but Rac1 and ARF6 dependent route [13]. Similarly, opsonised *L. amazonensis* amastigotes enter CHO cells via a Rac1-mediated pathway [14], while non-opsonised parasite entry is Cdc42 and RhoA dependent but Rac1 independent. Further research is required to delineate all host factors required for phagocytosis of *Leishmania* parasites, especially the amastigote stages. This knowledge could enhance our mechanistic understanding of this vital process, leading to therapeutic intervention to prevent parasite maintenance and dissemination within the mammalian host. This current study utilises genome-wide RNAi-based screening methods to investigate the effect of knocking-down expression of specific host proteins on parasite uptake.

The fruit fly, *Drosophila melanogaster*, has been widely utilised as a model eukaryote with approximately 7,200 of its 13,676 genes sharing identity with *C. elegans* or *M. musculus* genes [15]. Forward genetic screens, in which genes are identified by their mutant phenotypes in P-element transposon or chemically-induced mutagenesis screens, have been extremely successful in *Drosophila* [16]. Sequencing of the genome [15] and the development of powerful genetic tools, including RNAi libraries, now allow genome-wide reverse genetic screening of gene function across a wide range of biological processes. Therefore, the study of *D. melanogaster* may rapidly reveal information on conserved processes in other, less genetically-tractable organisms [17].

95% of *Drosophila* haemolymph cells are specialised phagocytic cells termed plasmatocytes [18], [19]. Like mammalian phagocytes, *Drosophila* plasmatocytes express a wide diversity of cell surface receptors that mediate particle recognition. Many of these share identity with mammalian receptors: for example the apoptotic cell receptor Draper [20] shares identity with Jedi-1, an apoptotic cell receptor expressed by murine glial cells [21]. S2 cells were isolated from fruit fly embryos [22] and probably originate from embryonic plasmatocytes. They demonstrate haemocyte-like gene expression and, like plasmatocytes, exhibit robust phagocytosis [19], [22], [23]. The discovery that S2 cells bathed in specific dsRNAs can rapidly endocytose the RNA, leading to reduced expression of target genes, has advanced functional genomic studies in *Drosophila* [24], [25]. Development of dsRNA libraries targeting the entire or partial coding *Drosophila* genome has allowed high throughput screening of S2 cells for novel factors in processes such as establishment of cell morphology [26], Golgi organisation [27] and cell signalling [28]. S2 cells have also been used to analyse phagocytosis of a range of prokaryotic pathogens including *Mycobacterium fortuitum* [29], *Listeria monocytogenes* [30], [31], *Chlamydia* [32], [33], and the eukaryotic fungus, *Candida albicans* [34], demonstrating their utility for studying host-pathogen interactions.

To date, S2 cells have not been used to investigate phagocytosis of a protozoan parasite. In this study, we characterise these cells as a novel genetically tractable *in vitro* model for studying *Leishmania*-host cell interactions. We demonstrate that S2 cells can take up *Leishmania* parasites with subsequent parasite replication and show that entry utilises similar mechanisms to those used by parasites to enter mammalian cells. Using this model, a RNAi screen against 1920 host-factors reveals several pathways that are essential for uptake of the parasite and one of these has been validated in mammalian macrophages, demonstrating the utility of this approach for identifying conserved and novel pathways for parasite entry.

## Results and Discussion

### *Leishmania* Amastigotes can Enter S2 Cells

**Figure 1:**
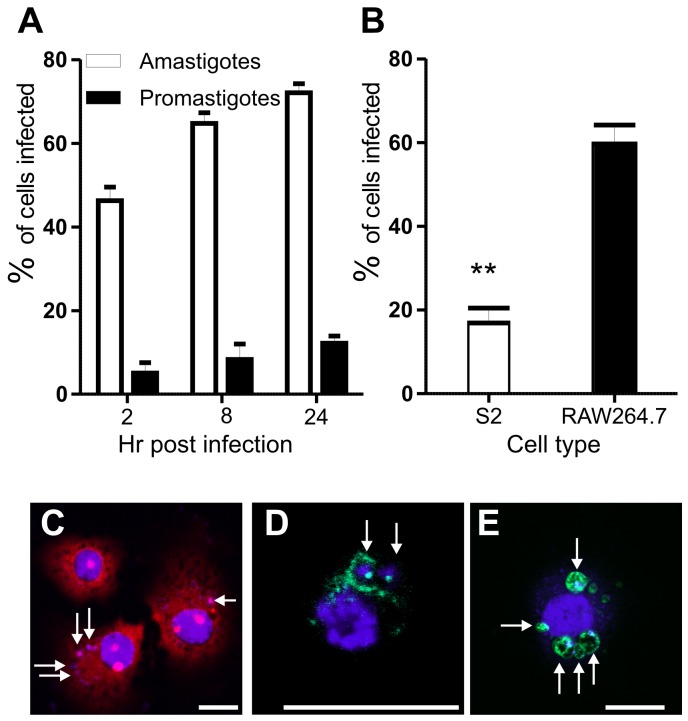
Infection of S2 cells with different *Leishmania* species and life cycle stages. A. Percentage of S2 cells infected after incubation with either *L. donovani* amastigotes or *L. major* late stationary phase promastigotes over a 24 hr time course. B. Comparison of infection levels of S2 cells and mammalian RAW264.7 macrophages incubated with late stationary phase *L. major* promastigotes. p = 0.0025; error bars in A and B indicate one standard error (SE). C. S2 cells infected with *L. donovani* amastigotes for 24 hr are stained with 1 µg/ml DAPI, 20 µg/ml propidium iodide; parasites were pre-labelled with 10 µM CFSE. D. S2 cells infected with *L. donovani* amastigotes (24 hr post infection) stained with anti-ARL8, a late endosomal/lysosomal marker. E. S2 cells expressing LAMP-GFP (green) infected with *L. donovani* amastigotes for 12 hr. C – E, scale bars, 10 µm. Arrows indicate intracellular parasites.

To determine whether *Drosophila* S2 cells can host *Leishmania* parasites, S2 cells were incubated with CFSE (5,6-carboxyfluorescein diacetate succinimidyl ester)-labelled *L. donovani* amastigotes or *L. major* late stationary (metacyclic-enriched) promastigotes and maintained over a time course. Infected cells were detected by staining and scoring of intracellular parasites (Figure 1). Staining with DAPI (4′,6-diamidino-2-phenylindole) allowed visualisation of DNA, while staining with propidium iodide (PI) in 0.1% saponin allowed clear visualisation of cytoplasmic RNA and intracellular organelles (Figure 1C), and therefore parasite internalisation. *L. donovani* amastigotes but not *L. major* promastigotes were readily phagocytosed (Figure 1A, C), with up to 70% of S2 cells infected by amastigotes while <10% of S2 cells contained promastigotes. *L. major* promastigotes at the same stage of development could, however, be efficiently phagocytosed by RAW264.7 murine macrophages (Figure 1B) with ∼60% of cells infected, suggesting that the defect in S2 cell phagocytosis of promastigotes is not due to parasite damage or other factors affecting the pathogen surface. A more likely explanation is due to the large size of promastigotes as compared with amastigotes and a lack of sufficient S2 cell membrane (S2 cell diameter 10–25 µm) to engulf these relatively large flagellated cells (10–15 µm); amastigotes are smaller (approx. 2–4 µm) and have only a rudimentary flagellum. Supporting this hypothesis, S2 cells have previously been shown to rapidly engulf *C. albicans* cells, which are approximately the same size as *L. donovani* amastigotes (∼4 µm) and the largest particles shown to be phagocytosed by S2 cells to date [34]. Alternatively, either S2 cells may not express suitable receptors for the recognition of *L. major* promastigotes or amastigotes may enter via pattern recognition receptors specific for amastigote surface molecules. Our limited observations on *Leishmania* size versus life cycle stage phagocytosis in S2 cells currently await further investigation.

**Figure 2:**
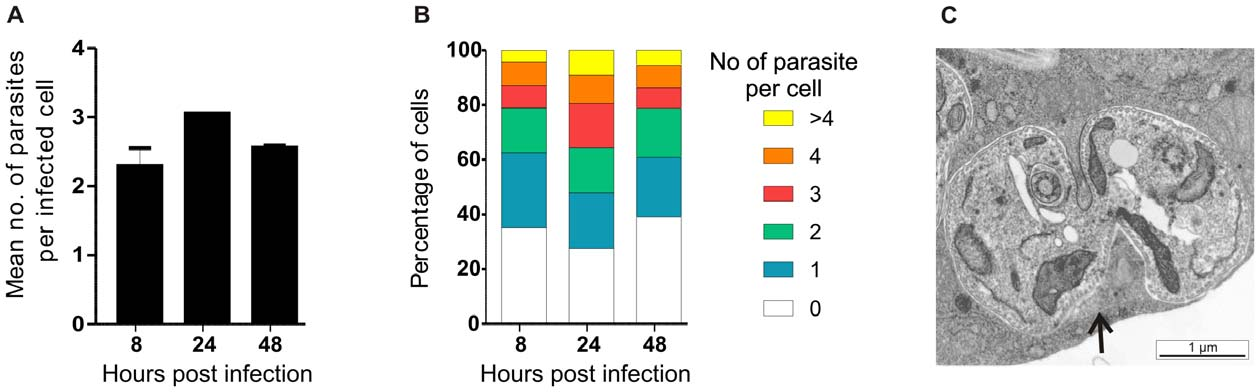
Maintenance of *L. donovani* amastigotes in S2 cells. S2 cells were incubated with *L. donovani* amastigotes for 8 hr, external parasites removed by washing and fresh medium returned to the cells prior to fixation at the time points indicated. A. Mean number of parasites per cell at each time point. Data were analysed by two way unpaired Student’s t-test (not significant). B. Distribution of the number of parasites per cell at each time point. C. Transmission electron micrograph of S2 cell following incubation with *L. donovani* amastigotes for 24 hr. An amastigote in the process of cell division is shown, with the phagosome membrane clearly separated from the dividing parasite (black arrow). The sub-pellicular microtubules and flagella of the dividing parasites are also clearly visible (white arrows).

Given the role of *L. donovani* as the causative agent of the most severe form of leishmaniasis (VL) and our lack of knowledge of the critical mechanisms used by amastigotes to enter host cells, further experiments were undertaken to characterise S2 cells as a model for studying host-amastigote interactions. Transmission electron micrographs of infected S2 cells revealed that phagocytosed amastigotes are contained within a tight phagosome (Figure 2C) which is positive for the lysosomal marker, ARL8 (Figure 1D) [35]. Infection of S2 cells expressing LAMP1 (lysosomal membrane protein 1) -GFP also revealed that parasites are held in a LAMP1-positive compartment (Figure 1E). Together, these data suggest that, like amastigote-containing phagosomes in mammalian cells [13], [36], S2 cells maintain phagocytosed parasites within compartments that share characteristics of phagolysosomes.

### *Leishmania* Amastigotes are Able to Replicate in S2 Cells

Incubation of S2 cells for 8 hr at 26°C with *L. donovani* amastigotes, followed by removal of external parasites and fixation and counting of intracellular parasites over a time course, revealed that mean parasite burdens were maintained at ∼2 amastigotes per cell for 8–48 hr (Figure 2A, B). Transmission electron microscopic analysis of cells infected for the same period, revealed parasites in the process of dividing (Figure 2C). Taken together these data suggest that parasite replication was occurring within the S2 cells, possibly balanced by parasite killing. By 48 hr, we observed some changes in the morphology of the intracellular amastigotes, suggesting that by this time point at 26°C, the amastigotes (which are usually maintained at 37°C in mammalian macrophages) begin to differentiate to their promastigote morphology. The survival of *Leishmania* in S2 cells beyond 48hr was not studied.

**Figure 3:**
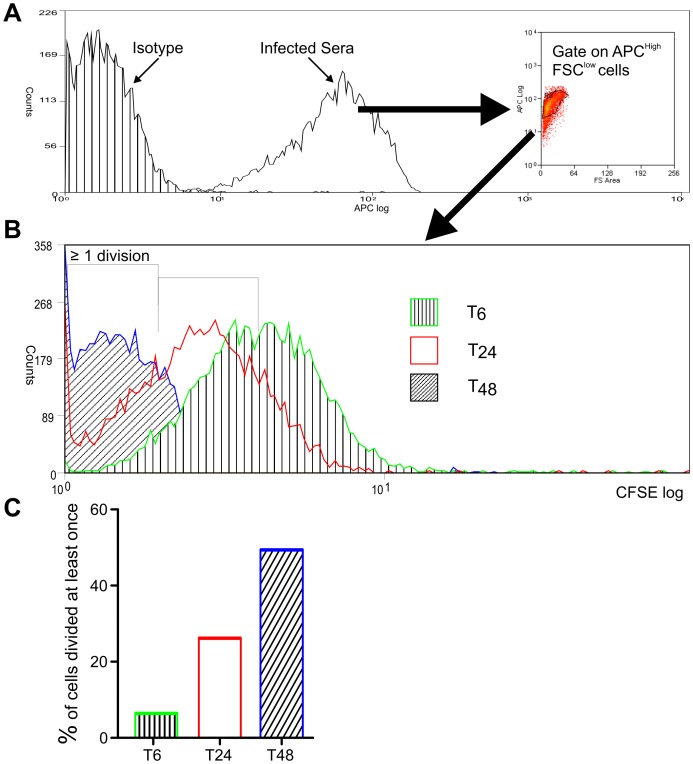
Replication of *L. donovani* amastigotes within S2 cells. S2 cells were infected with CFSE-labelled parasites for 6 hr, followed by washing to remove external parasites. At the time points indicated, S2 cells were lysed with saponin to release amastigotes. A. Parasites were identified from cell debris by staining with the sera from an infected hamster conjugated to fluorescent allophycocyanin (APC), followed by flow cytometry and gating on APC high, forward scatter low cells. B. Flow cytometry overlay of CFSE staining of parasites identified in A, released after 6, 24 and 48 hr. C. By gating on CFSE staining in B, the percentage of parasites that had replicated at least once was calculated at each time point. Data are representative of two independent experiments.

To further investigate parasite replication following phagocytosis, amastigotes labelled with CFSE were incubated with S2 cells for 6 hr, followed by washing to remove external parasites. The S2 cells were then lysed at the time points indicated, the parasites identified by staining with serum from a *L. donovani*-infected hamster (as described in [37]) and their CFSE-staining calculated following separation by flow cytometry (Figure 3). These data reveal that by 48 hr post infection, 50% of parasites had replicated at least once in S2 cells (Figure 3C). By comparison, Phillips and colleagues [37] found that 80% of *L. donovani* amastigotes phagocytosed by RAW264.7 murine macrophages had replicated over the same time period. This difference is not unexpected, given that S2 cells are not the natural host of *Leishmania* parasites and that the parasites were maintained at 26°C in these experiments. Overall, as amastigotes are rapidly and easily phagocytosed by S2 cells in which they can also replicate, it can be concluded that S2 cells provide a good model in which to study early molecular events required for parasite uptake.

### Amastigotes Enter S2 Cells by Conserved Pathways

**Figure 4:**
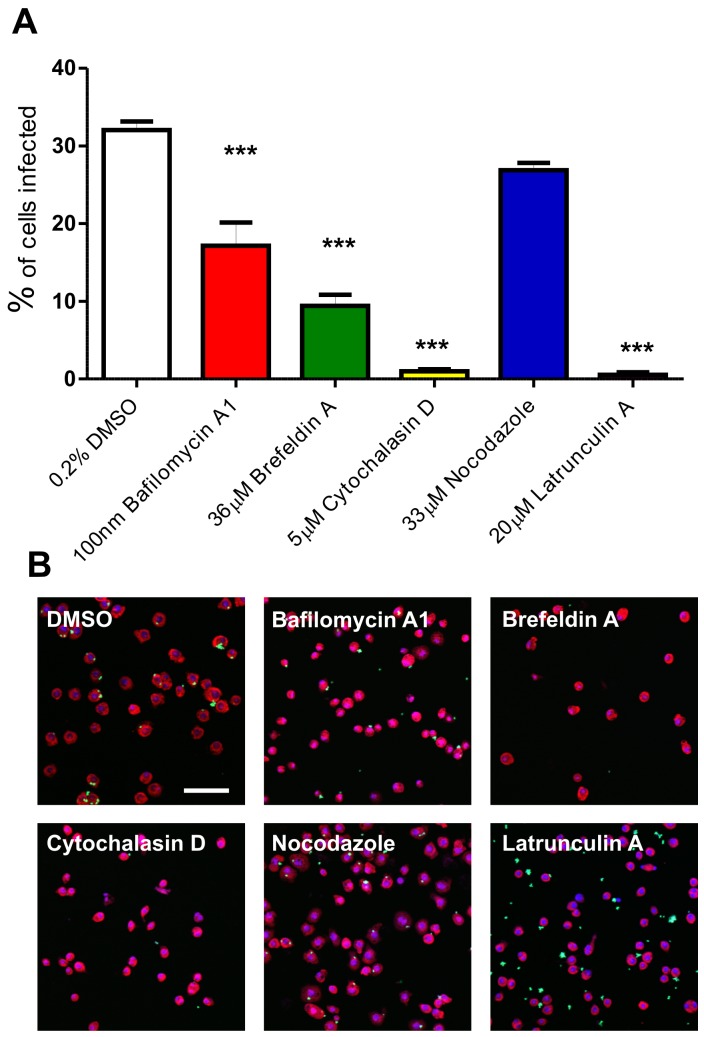
Effect of pharmacological inhibitors on S2 cell phagocytosis of *L. donovani* amastigotes. S2 cells were treated with inhibitory compounds for 1.5 hr prior to incubation with CFSE-labelled *L. donovani* amastigotes (at a 10∶1 ratio) for 3 hr in the continuing presence of these drugs. A. Percentage of S2 cells infected with *L. donovani* after treatment with the compounds indicated. Approximately 300 cells in 3 independent wells were scored by eye for each treatment. Figures were compared to DMSO-treated controls by one-way analysis of variance (Anova) with Dunnett’s Multiple Comparison Test, *** p<0.0001. Bars indicate one standard error of the mean. B. Representative confocal laser scanning microscopy images of cells treated with the compound indicated. Cells were stained with 1 µg/ml DAPI, 20 µg/ml propidium iodide; amastigotes were stained with CFSE; size bar, 50 µm.

*Leishmania* entry into mammalian cells by host-mediated phagocytosis requires regulation of assembly of the host actin cytoskeleton, an essential component for parasite uptake. In order to confirm the role of actin in *L. donovani* uptake and identify other host pathways required for phagocytosis, S2 cells were incubated with a panel of inhibitory compounds prior to, and during infection with, *L. donovani* amastigotes (Figure 4). Compounds were selected to focus on cytoskeletal and endocytic processes. Live/dead cell staining methods revealed that these treatments were not cytotoxic to parasites or to S2 cells (data not shown). Cytochalasin D and latrunculin A, two inhibitors of actin polymerisation, almost entirely abolished uptake of *L. donovani* amastigotes. These data indicate that, as in mammalian cells, phagocytosis of amastigotes by S2 cells requires dynamic rearrangement of the actin cytoskeleton [10]. In contrast, nocadazole-mediated depolymerisation of microtubules [38] had no significant effect on phagocytosis (Figure 4), indicating that host tubulin is not required for phagocytosis. Inhibition of vacuolar-type H^+^-ATPases with bafilomycin A1 reduced phagocytosis by 50%, suggesting a role for endosomal/lysosomal acidification in uptake of the parasite. Similarly, brefeldin A inhibition of ARF1-mediated vesicular transport [39], [40], [41] resulted in a 73% reduction in phagocytosis. As described above, phagocytosis of lesion-derived *L. donovani* amastigotes by mammalian phagocytes is FcγR and Cdc42 independent but Rac1 and ARF6 dependent [13]. As phagocytosis in S2 cells is brefeldin A sensitive, this could suggest that S2 cells may not be utilising the same pathways as those used by macrophages. However, the above studies utilised IgG-coated parasites, whereas in our study, parasites were derived from immunoglobulin-deficient mice (and therefore non-opsonised) and applied to invertebrate-derived cells (that are therefore unlikely to bear Fc receptors). Thus, our data suggest that parasites enter S2 host cells by an ARF1-dependent mechanism. This is distinct to S2 cell uptake of *Brucella abortus* which has been shown to be brefeldin A insensitive [42].

**Figure 5:**
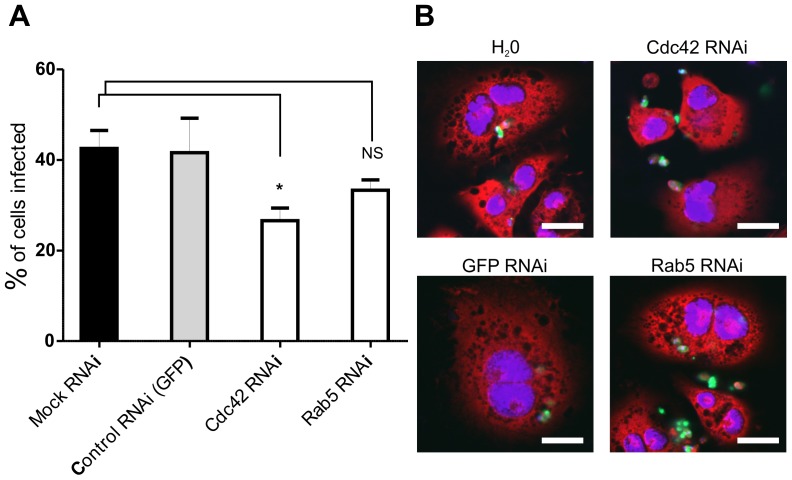
RNAi against Cdc42 reduces phagocytosis of *L. donovani* amastigotes. S2 cells were treated with dsRNA against Cdc42 and Rab5 for 4 days and then incubated with amastigotes for 24 hr. A. Three wells from a 96 well plate were analysed for each treatment and at least 100 cells counted in each well. * p<0.05. B. Representative confocal laser scanning microscopy images of cells treated with dsRNA as indicated. Cells were stained with 1 µg/ml DAPI, 20 µg/ml propidium iodide; amastigotes were stained with CFSE; size bar, 10 µm.

To further validate the use of S2 cells as a model for studying *L. donovani* amastigote uptake, these cells were treated with dsRNAs against two host factors previously shown to be required for phagocytosis. The small GTPase, Cdc42, is essential for phagocytosis of *Salmonella* and *L. amazonensis* amastigotes (within mammalian Chinese hamster ovary (CHO) cells) [14], [43]. As shown in Figure 5, knock-down of Cdc42 in S2 cells results in a 40% reduction in amastigote uptake, providing good correlation with the previously-published data. RNAi knock-down of Rab5, a second molecule shown in RNAi screens to be required for phagocytosis and maintenance of intracellular pathogens [29], caused a 20% reduction in amastigote uptake although this figure was not statistically significant in this analysis. This observation suggests that either this protein is not essential for phagocytosis of *Leishmania* amastigotes in S2 cells or that the levels of knock-down achieved were not sufficient to observe a biological effect. Overall, however, the experiments described in Figure 5 provide good evidence to support the use of S2 cells for the study of amastigote phagocytosis, thereby facilitating a high-throughput screening approach to identify multiple factors required for this process.

### RNAi Screen for Novel Regulators of Phagocytosis

**Figure 6:**
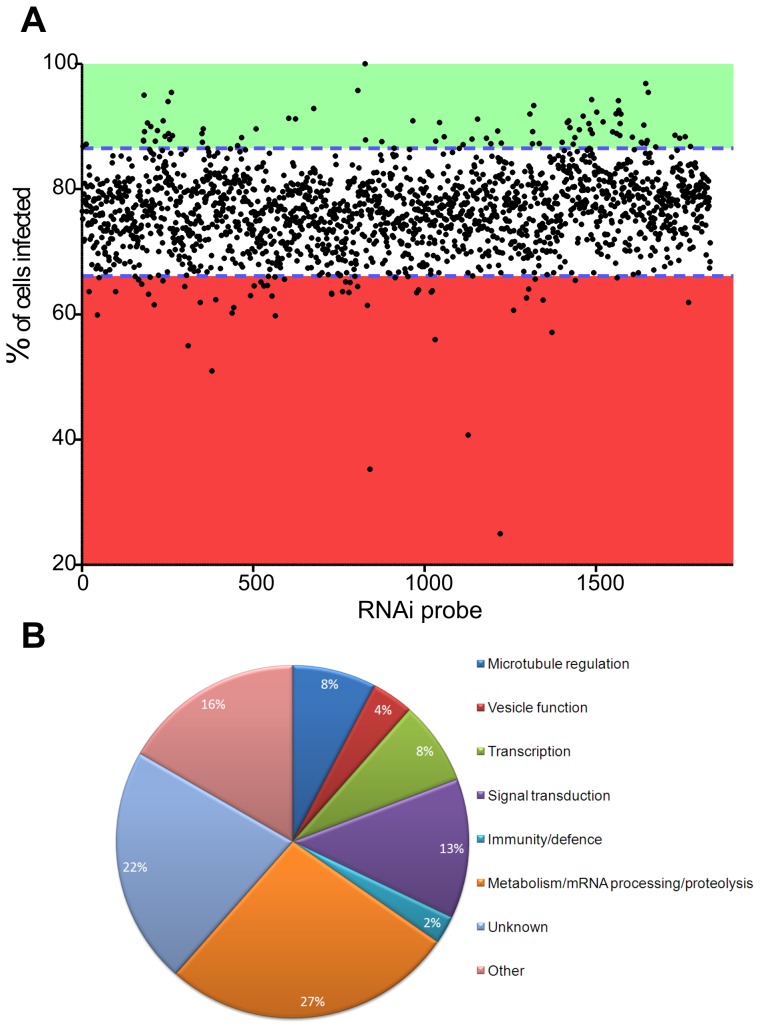
Results from RNAi screen against 1920 *Drosophila* gene products. A. Scatter plot of effect of dsRNA on uptake of *L. donovani* amastigotes. Each point represents an individual dsRNA treatment. dsRNA probes are ordered on the x axis by probe number (1–1920). Hits outside 1.5 standard deviations from the mean (blue dashed line) were chosen for further analysis. Green shading indicates hits that caused a significant increase in phagocytosis. Red shading indicates hits that caused a significant decrease in phagocytosis. B. Predicted GoFunctions of 87 proteins for which RNAi mediated knock-down of gene expression resulted in a significant decrease in phagocytosis of *L. donovani* amastigotes by S2 cells.

In order to identify further regulators of parasite uptake, S2 cells were treated with dsRNA generated from the first 1920 probes of a *Drosophila* Expression Arrest™ RNAi dsDNA Library (Data S1) which is designed to target gene products that are also conserved in *C. elegans* and *M. musculus* [17]. This unbiased selection represents approximately 30% of the total population of genes conserved across metazoan species. Using the methods described under Experimental Procedures (see also Data S2 for overview of screening process), S2 cells were incubated with specific dsRNAs for 4 days (to achieve optimal knock-down of gene expression, see Data S3) prior to infection with CFSE-labelled *L. donovani* amastigotes for 22 hr. After transfer to ConA-coated imaging plates, infected cells were fixed, stained and imaged by automated confocal microscopy. To distinguish between infected and uninfected cells, algorithms were developed in CellProfiler, an open source image analysis program [44] to analyse each of the 23,000 images generated (analysis ‘pipeline’ can be found in Data S4). Automated scoring gave an output for the number of cells infected after each RNAi treatment and these values are plotted in Figure 6A. Hits with an infection rate ±1.5 standard deviations were chosen for further analysis, a cut-off utilised previously in other similar screens [34]. Using this cut-off, 87 dsRNA treatments significantly decreased infection rates and 84 significantly increased infection rates (Data S5). Treatments that significantly decreased infection rates were chosen for further analysis here. Analysis of these 87 gene products revealed proteins with predicted functions in cell signalling, cytoskeletal processes and gene transcription as well as a large number of genes with no predicted function to date (Figure 6B).

### Curation of the RNAi Screen Hit List

In high content screens, it is necessary to distinguish true, biologically relevant hits from false positives. These can result from a variety of processes, including off target effects of the RNAi (see below), but can also result from knocking down a protein that plays a key role in multiple processes essential for cell viability. With this rationale in mind, fifteen hits annotated with roles in proteolysis, translation and RNA processing were removed from further analysis at this time (Data S6). Similarly, hits previously shown to reduce viability in a *Drosophila* RNAi screen [45] and hits identified visually in this screen as causing a major reduction in S2 cell numbers were removed prior to second round screening (Data S7).

Off target effects (OTEs) present a significant problem in high content RNAi screens [46], [47], with dsRNAs containing ≥19 nucleotide (nt) perfect matches able to generate these potential artefacts [46]. Additionally, one study identified 13 nt CAN trinucleotide repeats (with N representing any base) as sufficient to induce OTEs when studying the Wingless signalling pathway in *Drosophila* [47]. The library utilised in the screen reported here [17] was generated at a time when OTEs were poorly understood. Thus, many of its probes share ≥19 nt identity with other targets. To reduce further problems, all dsRNAs causing a significant decrease in parasite uptake in Figure 6A were analysed for CAN repeats and predicted 19 nt identity with other *Drosophila* mRNAs [48]. This resulted in removal of nine hits from further analysis as their dsRNA contained more than four predicted OTE regions (Data S8).

### Second Round Screening for Hit Validation

Further refinement of the hit list generated from Figure 6A, as described above, resulted in 34 targets that are predicted to function in the phagocytosis of *L. donovani* amastigotes. These are listed in Data S9. Even when using OTE-free probes, a robust method to determine whether a hit is real or artefactual is to generate a second RNAi probe to a new region of the transcript and monitor whether the observed phenotype is reproducible [49].

Second round RNAi experiments were therefore performed against 12 hits with both new validation probes and the original probe (if OTE free, Data S10). In addition, two hits (Neuroglian and Stx5) which increased infection in the primary screen were also chosen for further analysis at this stage. Although Rab5 and SCAR were not significant hits in the primary screen in Figure 6A, these molecules have been shown to be key regulators of phagocytosis in other screens [29], [30], [31], [32], [34], [50]. Indeed, 66.38% of cells treated with dsRNA targeting SCAR (CG4636) were infected, only 0.34 percentage points outside the cut-off for significant hits. Therefore both SCAR and Rab5 (using a different OTE free probe to that used in the primary screen and in the experiments described in Figure 4) were also included in the second round knock down experiments.

**Figure 7:**
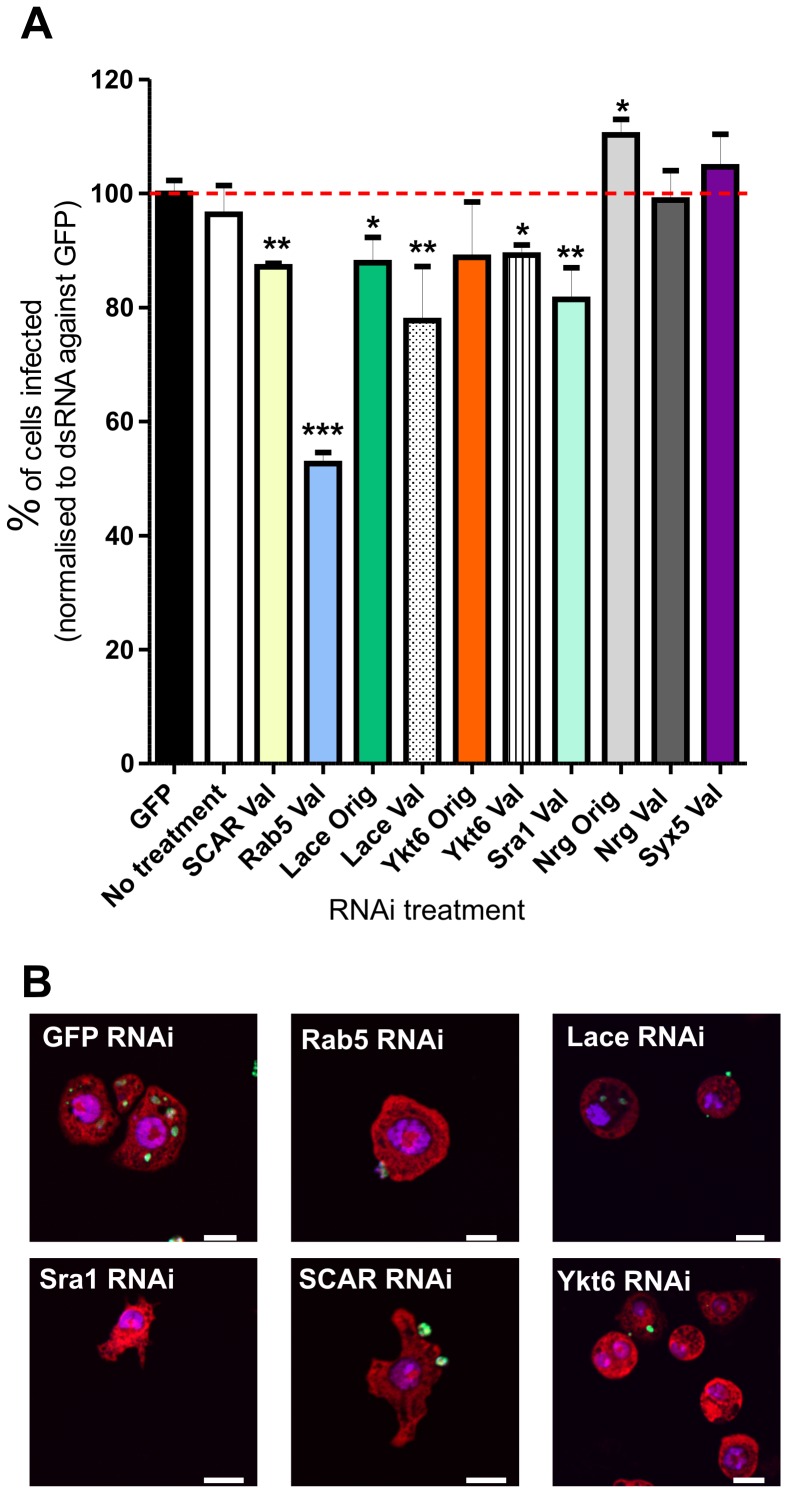
Validation RNAi experiments. A. Percentage of cells infected after RNAi with dsRNA validation (Val) and original (Orig, if OTE free) probes (as indicated). Infection rates normalised to dsRNA GFP control treated cells. Error bars indicate one standard error of the mean. Wells were imaged on a Zeiss LSM 510 confocal laser scanning microscope, and infection rates scored manually. A mean of 154 cells were counted per well, with three replicates of each dsRNA treatment and eight replicate GFP dsRNA treatments. Infection rates were normalised to GFP infection rates (100.0% ±2.264 [1 SEM]). Infection rates compared by two way unpaired Student’s t-test *, p<0.05; **, p<0.01; *** p<0.001. B. Representative images of RNAi treated cells. Bar indicates 10 µm.

Validation screening was performed as described and infection rates were normalised to those achieved with control dsRNA GFP (Figure 7). Of the 14 targets tested in this secondary screen, 5 showed significant down-regulation; these were *Scar, Rab5, lace, YKT6* and *Sra1.*

*lace* encodes serine palmitoyltransferase long chain base subunit 2 (*spt2*), one subunit of serine palmitoyltransferase (SPT), an enzyme that catalyses condensation of serine and palmitoyl CoA to produce 3-ketodihydrosphingosine in the first step of the sphingolipid synthesis pathway [51]. Second round screening with both a *lace* original and new validation probes resulted in a significant reduction in phagocytosis (Figure 7).

RNAi mediated knockdown of CG1515 resulted in decreased S2 cell infection rates. CG1515 encodes YKT6, a prenylated nonsyntaxin SNARE protein which has been implicated in ER to Golgi transport, intra-Golgi transport and homotypic vacuole fusion [52], [53], [54]. As this protein has multiple functions, it is difficult at this stage to propose its precise role in the mechanism of phagocytosis. However, studies have suggested that YKT6 acts in conjunction with Cdc42 to generate actin forces required for vacuole fusion [55], possibly linking YKT6 to Cdc42-generated actin polymerisation during phagocytosis. Interestingly, YKT6 has also been found by RNAi screening to be essential for phagocytosis and/or survival of *C. trachomatis*, *L. monocytogenes* and *M. fortuitum*, suggesting a conserved role in this process [29], [30], [31], [50].

The Rab5 validation RNAi probe caused the greatest defect in phagocytosis in the secondary screen, despite a different dsRNA probe not causing a significant decrease in preliminary experiments (Figure 4). This observation highlights the issue of probe choice and how distinct RNAi probes against the same target can produce different effects. Rab5 has already been shown to be important for phagocytosis of many bacteria [29], [30], [31]. Although these data could suggest a common requirement for Rab5 in phagocytosis, uptake was not separated from replication in these assays. In contrast, Rab5 was not a hit in screens in which phagocytosis was directly scored [34], [56]. Rab5 has been shown to regulate fusion of *L. donovani* promastigote phagosomes in murine macrophages, although no data were provided in this study on whether phagocytosis was increased in cells expressing a dominant-active form of Rab5 [57]. Interestingly, *Trypanosoma cruzi* infection is abrogated in macrophages expressing a dominant-negative form of Rab5 [58], perhaps suggesting a conserved role for Rab5 in host cell entry by kinetoplastid parasites.

Sra1 (specifically Rac1 associated protein 1) and SCAR knock-down with validation probes also caused significant decreases in the number of cells infected (Figure 7). The Sra1 gene product is already described as playing a role in *L. monocytogenes* and *M. fortuitum* phagocytosis [29], [31] and is known to regulate the actin nucleation pathway through interactions with SCAR and Rac1 [59], [60]. Like Sra1, SCAR also regulates the actin cytoskeleton, reorganisation of which is essential for phagocytosis [19]. RNAi of Sra1 and SCAR resulted in abnormally shaped cells suggesting potential disruption of the cytoskeleton (Figure 7B). The number of “actin-regulating” hits identified in the secondary screen described in Figure 7 supports the hypothesis that alternative host signalling pathways can operate in the uptake of *Leishmania* amastigotes. In the first pathway, non-opsonised parasites would bind to an unknown S2 cell surface receptor leading to a signalling cascade and Cdc42 activation. Cdc42 normally signals via WASP [61], although a role for this protein was not investigated in the current RNAi screen (as WASP was not included as a target gene). WASP activates the ARP2/3 complex and, in this model, could lead to actin nucleation [62] and amastigote phagocytosis. In the second alternative pathway, parasites could ligate an alternative surface receptor that signals via Rac1. Sra1 mediated repression of SCAR is removed upon Rac1-GTP binding, leading to ARP2/3 stimulation and actin nucleation [63]. Rac1 itself was not identified in this RNAi screen as *Drosophila* Rac1, Rac2 and Rac-like Mtl are known to be functionally redundant [64]. It is relevant that parasite phagocytosis was abolished by the addition of latrunculin A and cytochalasin D (Figure 4), two inhibitors of actin branching and polymerisation, demonstrating a requirement for dynamic actin rearrangements during amastigote uptake by S2 cells.

### Myriocin Treatment of Mammalian Macrophages Prevents Phagocytosis of both Amastigotes and Promastigotes

**Figure 8:**
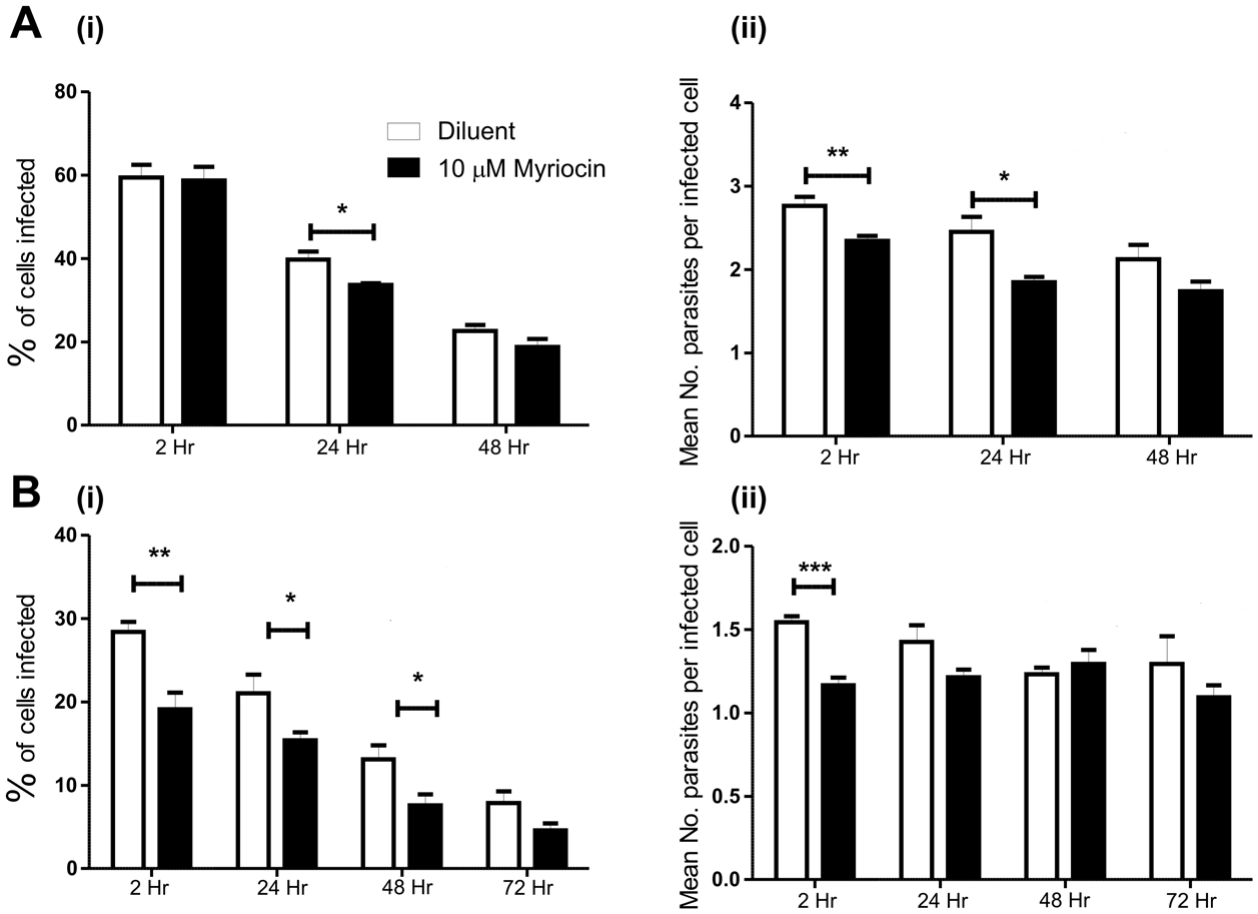
Effect of myriocin on RAW264.7 macrophage phagocytosis of *L. donovani* amastigotes and promastigotes. RAW264.7 macrophages were pre-treated with 10 µM myriocin or 0.5% methanol (diluent) for 24 hr to inhibit *de novo* sphingolipid biogenesis. They were then incubated with *L. donovani* parasites (at a MOI of 10∶1) for 2 hr, external parasites removed and the infected macrophages fixed or incubated with medium containing myriocin or methanol for the periods indicated. A. Infection with *L. donovani* amastigotes; B. Infection with *L. donovani* promastigotes. (i) Percentage of cells infected at the timepoints indicated; (ii) Mean number of parasites per infected cell. Infections rate compared by two way unpaired Student’s t-test * p<0.05, ** p<0.01, *** p<0.001. Error bars indicate one standard error of the mean.

To independently validate hits from the *Drosophila* RNAi screen in mammalian cells, we focused on one target, SPT. RAW264.7 macrophages were treated with 10 µM myriocin, a specific inhibitor of SPT, for 20 hr followed by incubation with *L. donovani* parasites for 2 hr in the presence of inhibitor. When amastigotes were incubated with RAW264.7 cells pre-treated with myriocin, there was a significant decrease in infection rates at 24 hr (Figure 8A). Interestingly, there was also a significant decrease in the mean number of internalised parasites per infected cell at both 2 hr and 24 hr. These data suggest that *de novo* sphingolipid biosynthesis is required for the uptake and possibly replication of amastigotes in a mouse macrophage cell line. Similarly, myriocin-treated RAW264.7 macrophages infected with late stationary *L. donovani* promastigotes, showed much lower infection rates than cells treated with methanol as a control (Figure 8B). At 2, 24 and 48 hr, there were significantly decreased infection rates, with a significant reduction in parasite number per infected cell at 2 hr.

Sphingolipids such as ceramide, sphingomyelin and glycosphingolipids contain two saturated acyl chains that can pack tightly in the membrane to form rigid lipid microdomains (also termed lipid rafts). Unesterified cholesterol can also intercalate with these sphingolipids, increasing the packing density. These detergent resistant membranes are thought to allow compartmentalisation of the plasma membrane and generate distinct regions for signalling or protein interactions [65]. Many Fcγ receptors are recruited to or near lipid rafts during phagocytosis bringing them into proximity to the Src kinases required for initiating phagocytosis [66]. We hypothesise that disruption of sphingolipid biosynthesis leads to altered sphingolipid concentrations and lipid raft dynamics, resulting in aberrant receptor recruitment and downstream effects on phagocytosis. Further analysis of myriocin-induced alterations to the abundance and distribution of different lipids species will be required to further validate this interpretation.

## Concluding Remarks

This paper describes the development of a new model for investigating host-*Leishmania* interactions, focussing on the intracellular parasite stages that give rise to the most serious form of human leishmaniasis. *L. donovani* amastigotes are phagocytosed by *D. melanogaster* macrophage-like S2 cells, in which they reside and replicate within mature phagolysosomes. Using a combination of inhibitor studies and an RNAi screen against 1920 metazoan-conserved host factors, a range of genes and molecular pathways required for uptake and survival of *L. donovani* amastigotes have been identified. As proof-of-principle, *lace*, which encodes a subunit of host serine palmitoyltransferase (SPT), has been identified in the RNAi screen as an important factor for the uptake of parasites by S2 cells, while specific inhibitor studies demonstrate that SPT activity is required for efficient phagocytosis of both *L. donovani* amastigotes and promastigotes in mammalian macrophages. These data validate the high throughput screening approach and suggest that further analysis will identify new components to inform our understanding of the molecular pathways used by amastigotes to enter and survive within host cells.

The CellProfiler image analysis pipeline developed in this project can be used for automated image analysis of any labelled host cell, phagocytosing any labelled organism. A similar image-based screen has recently been used to identify drug compounds that inhibit amastigote growth in human macrophages [67]. As this analysis was performed on proprietary software (which could be prohibitively expensive for many research groups), the pipeline described here is available for download in Data S4.

## Materials and Methods

### Ethics Statement

All experiments were approved by the University of York Animal Procedures and Ethics Committee and performed under UK Home Office license (‘Immunity and Immunopathology of Leishmaniasis’ Ref # PPL 60/3708).

### Cell and *Drosophila* Culture/propagation

S2 cells were obtained from the *Drosophila* Genomics Resource Centre and grown at 26°C in Schneider’s *Drosophila* medium supplemented with 10% FCS (Hyclone) plus 100 units/ml penicillin and 100 µg/ml streptomycin. RAW264.7 murine macrophage-like cells were obtained from ATCC and maintained in complete DMEM. The plasmid pMT DmLAMP-GFP (a kind gift of Gudrun Ihrke, Uniformed Services University of the Health Sciences, Bethesda, USA) was digested with *Xho*I to release DmLAMP-GFP for subcloning into pAc5.1 (Invitrogen), generating pAc5.1-DmLAMP-GFP. This plasmid was transfected into S2 cells using CellFectin reagent (Invitrogen) and standard protocols.

*L. major* Friedlin clone V1 (MHOM/IL/81/Friedlin) and *L. mexicana* (MNYC/BZ0/62/M379) promastigotes were grown at 26°C in 1xM199 supplemented with 10% Gibco fetal bovine serum (FBS, Invitrogen) and penicillin-streptomycin (Invitrogen). *L. donovani* LV9 (MHOM/ET/67/L28/HU3) promastigotes were maintained in Donovani medium [68].

C57BL/6 (B6) recombination activating gene deficient mice (RAG1^−/−^), Originally obtained from Jackson Laboratories, Bar Harbor, USA [69], were intravenously inoculated with 3×10^7^ amastigotes into the lateral tail vein and infection maintained for 3–6 months. Mice were then sacrificed according to Home Office procedures and the spleen removed into RPMI. The spleen was homogenised to a single cell suspension in RPMI, centrifuged at 140 g for 5 min, the supernatant pipetted into a 50 ml Falcon tube coated with 1.25 mg/ml Saponin (Sigma) and incubated at room temperature for 5 min. The sample was then centrifuged twice at 2200 g for 10 min, the supernatant discarded, and the pellet washed three times by resuspension in 25 ml RPMI and centrifugation at 2200 g for 10 min. The pellet was then resuspended in 20 ml *Drosophila* medium, 20% FCS, passed through a 26 gauge needle and parasites counted on a haemocytometer.

### Infection of *Drosophila* S2 Cells

S2 cells were incubated with 10 µM CFSE-labelled parasites at a MOI (multiplicity of infection) of 10∶1 for the indicated time periods. At the indicated timepoint, the medium containing non-phagocytosed parasites was removed and the S2 cells washed three times to remove external parasites. To facilitate cell binding and spreading and improve resolution of the cytoplasmic area, S2 cells were dislodged by pipetting up and down vigorously three times and transferred, either to wells containing coated 13 mm 0.5 mg/ml Concanavalin A (Sigma) coverslips or to Concanavalin coated optical bottomed 96-well imaging plates (Greiner Bio-one). After 1 hr for adherence, cells were then fixed with 3.6% paraformaldehyde (PFA) for 30 min followed by staining with 1 µg/ml 4',6-diamidino-2-phenylindole (DAPI), 20 µg/ml propidium iodide (PI, Sigma) and 0.1% saponin (Sigma, w/v) in phosphate buffered saline (PBS). Where indicated, infected S2 cells were stained for immunofluorescence following fixation, permeabilisation with 0.1% Triton X100 and blocking with ImageIt (Invitrogen) for 30 min. Primary antibody incubations for 1 hr were followed by three 10 min washes in PBS and incubation with fluorophore-conjugated secondary antibodies for 1 hr. ARL8 antibodies were the kind gift of Sean Munro, Cambridge. Cells were DAPI stained, mounted and imaged on a LSM 510 Confocal Laser Canning Microscope (Zeiss) or Nikon Eclipse E600 fluorescent microscope with a Plan-Fluor 100x/1.30 oil objective lens.

Multiplicity of infection data were monitored by counting 200 S2 cells (using fluorescence microscopy, focusing up and down through each cell to confirm parasite internalisation). DAPI staining allowed visualisation of S2 cell and parasite nuclei, whereas PI also stained RNA within the cytoplasm allowing good discrimination of the host cell boundary.

### Cell Proliferation Studies

Parasite proliferation within S2 cells was monitored by loss of CFSE staining during cell division, using the methods described in Phillips *et al.* [37].

### Transmission Electron Microscopy

S2 cells were incubated with *L. donovani* amastigotes for 24 hr, harvested, the pellet resuspended in 500 µl 1% (w/v) glutaraldehyde for 1 hr, treated for 10 min in 1% (w/v) tannic acid, followed by 45 min in 0.5% (w/v) osmium tetroxide (all in 100 mM phosphate buffer), and then 1 hr in 1% (w/v) aqueous uranyl acetate. Cells were dehydrated in an acetone series and embedded in a Spurrs resin, before sections were cut with a Leica Ultracut, and then stained with Reynolds lead citrate plus saturated uranyl acetate in 50% ethanol. Sections were viewed with a Tecnai 12 BioTwin (FEI) at 120 kV and images acquired with a SIS MegaView III digital camera.

### Drug Treatment of Cells

5×10^4^ S2 cells per well were plated into a tissue culture-treated 96 well plate (BD Biosciences) and left overnight at 26°C to settle. Cells were treated with inhibitors at the concentrations indicated for 1.5 hr (with the exception of myriocin, see below) prior to addition of CFSE-labelled *L. donovani* amastigotes at 1×10^6^/well in 200 µl appropriate drug (or 0.25% DMSO as control). Following incubation for 3 hr, infected cells were dislodged by pipetting, transferred to a ConA-coated imaging plate and left to adhere for 1 hr. Cells were then fixed with 3.6% PFA for 30 min, stained with 20 µg/ml PI, 5 µg/ml DAPI, 0.1% saponin in 1× PBS and infection rates scored manually. RAW264.7 macrophages were treated with 10 µM myriocin (Sigma) for 20 hr prior to infection, with control cells treated with 0.5% methanol. Parasite infection was carried out in the presence of either myriocin or methanol. After 2 hr, macrophages were gently washed to remove external parasites and incubated in medium plus inhibitors until fixation at the time points indicated and processed as above.

### RNAi Screen

Primer sequences used for preliminary RNAi experiments and for the high content screen are provided as supplementary data (Data S1). For the high content screen, dsRNA was generated from the first 1920 probes of *Drosophila* Expression Arrest™ RNAi dsDNA Library 1.0 (purchased as dsDNA templates in 96 well format from OpenBiosystems). This library was designed to allow generation of 200–800 bp dsRNAs targeting exonic transcript regions of *Drosophila* genes with orthologues in *Caenorhabditis elegans* or *Mus musculus* [17]. T7 polymerase was generated and purified by the method of He *et al.* [70]. In vitro transcription reactions were carried out in a 20 µl volume reaction with 3 µg of DNA template, 5 mM rNTPs 0.015U/µl of yeast inorganic pyrophosphatase (Sigma), 0.2U/µl RNasin in a transcription buffer of 30 mM HEPES (pH 7.8), 100 mM potassium glutamate, 15 mM magnesium acetate, 25 mM EDTA, 1 mM DTT. Activity of T7 polymerase was assessed using these conditions and an optimal concentration per reaction employed for the library synthesis. Reactions were incubated at 37°C for 4 hr, RNAi was then diluted 5× by the addition of DEPC-treated H_2_O. Yield was assessed by agarose gel electrophoresis.

Validation probes against a new transcript region were designed using FlyBase and BLAST. Current validated RNAi probes were visualised by GenomeBrowse and the primer sequences plus *GGGTCCCT* site ordered for in-house PCR as above. Where no current probe was available, one was designed using SnapDragon RNAi design (http://www.flyrnai.org/cgi-bin/RNAi_find_primers.pl) or E-RNAi (http://www.dkfz.de/signaling/e-rnai3/). Again the *GGGTCCCT* linker was added to the 5′ end of the PCR primer and dsRNA generated as above.

For the high content screen, S2 cells were washed twice in Schneider’s *Drosophila* medium without FCS and resuspended at 1.5×10^5^/ml, and plated at 1×10^4^ cells per well in 96 well flat-bottomed tissue culture plates (Corning). RNAs generated from plates 1–20 of the *Drosophila* RNAi dsDNA Library 1.0 (OpenBiosystems) were defrosted, spun at 100 g for 1 minute and 5 µl of dsRNA pipetted into each well with RNase-free tips. RNA and cells were mixed by pipetting up and down gently 3 times. After 45 min, 133 µl Schneider’s *Drosophila* medium plus 10% FCS was added to each well, plates sealed with Polyolefin StarSeal (Starlabs) and incubated at 26°C for 4 days. On day 4, 100 µl of medium was removed and 25 µl of CFSE labelled RAG1^−/−^ spleen derived *L. donovani* amastigotes at 4×10^7^/ml added (1×10^6^/well). The plates were then sealed with new film and incubated for 24 hr at 26°C. Cells were then transferred to Con A (concanavalin A) -coated imaging plates, allowed to spread for 2 hr and stained with PI and DAPI as above. A 4 day RNAi treatment was chosen after quantifying mRNA and protein levels of a subset of hits after various lengths of incubation (Data S2 and S3).

### Imaging and Automated Image Analysis of Infected S2 Cells

Cells were imaged on an ImageXpress Ultra Confocal Laser Scanning Microscope (Molecular Devices) at the University of Nottingham, using filters and excitation lasers appropriate for DAPI, FITC and Texas Red. Wells were auto-focussed on DAPI-stained nuclei and all wells imaged with identical microscope settings. Four sites per well were imaged and data saved as 16 bit TIFFs. A “pipeline” was generated in CellProfiler [44] and this was used to score infection rates (see Data S2 and S4). dsRNA targets were removed from further analysis if they were found to be lethal. Lethal treatments were classified if they fell in one of two categories (i) already shown to be essential in S2 cells [45], or (ii) caused greater than 75% fewer cells than the average number of cells for the whole screen (scored visually).

## Supporting Information

Data S1 **Sequences of primers and subsequent dsRNA used in this study.** Sequences relate to first 1920 probes of *Drosophila* Expression Arrest™ RNAi dsDNA Library 1.0 (OpenBiosystems).
(XLS)

Data S2 **Schematic of RNAi Screen.**
(PDF)

Data S3 **Quantitation of ARL8 protein levels after RNAi mediated knock-down was used to determine optimal time period for maximum protein reduction.**
(PDF)

Data S4 **CellProfiler Pipeline.** This file must be opened in CellProfiler which can be downloaded here: http://www.cellprofiler.org/.
(MAT)

Data S5 **Infection rates after each dsRNA treatment.**
(PDF)

Data S6 **Hits removed from further analysis, as gene products predicted to be involved in proteolysis, translation or RNA processing.**
(PDF)

Data S7 **Targets removed from further analysis as their introduction led to cell death.**
(PDF)

Data S8 **Hits removed from further analysis as their dsRNAs contained multiple ≥19nt sequences with identity to other gene products and thus are predicted to generate off target effects.**
(DOCX)

Data S9 **Curated list of hits that decreased infection rates.**
(DOCX)

Data S10 **List of dsRNA probes used for validation screening.**
(DOCX)
