# Supplementary material for: Identification of Drosophila Gene Products Required for Phagocytosis of Leishmania donovani
Source: PLoS One. 2012 Dec 13;7(12):e51831. doi: 10.1371/journal.pone.0051831 (PMC3521716; doi:10.1371/journal.pone.0051831)
Supplement: Data S2 — Schematic of RNAi Screen. (PDF) [file pone.0051831.s002.pdf]

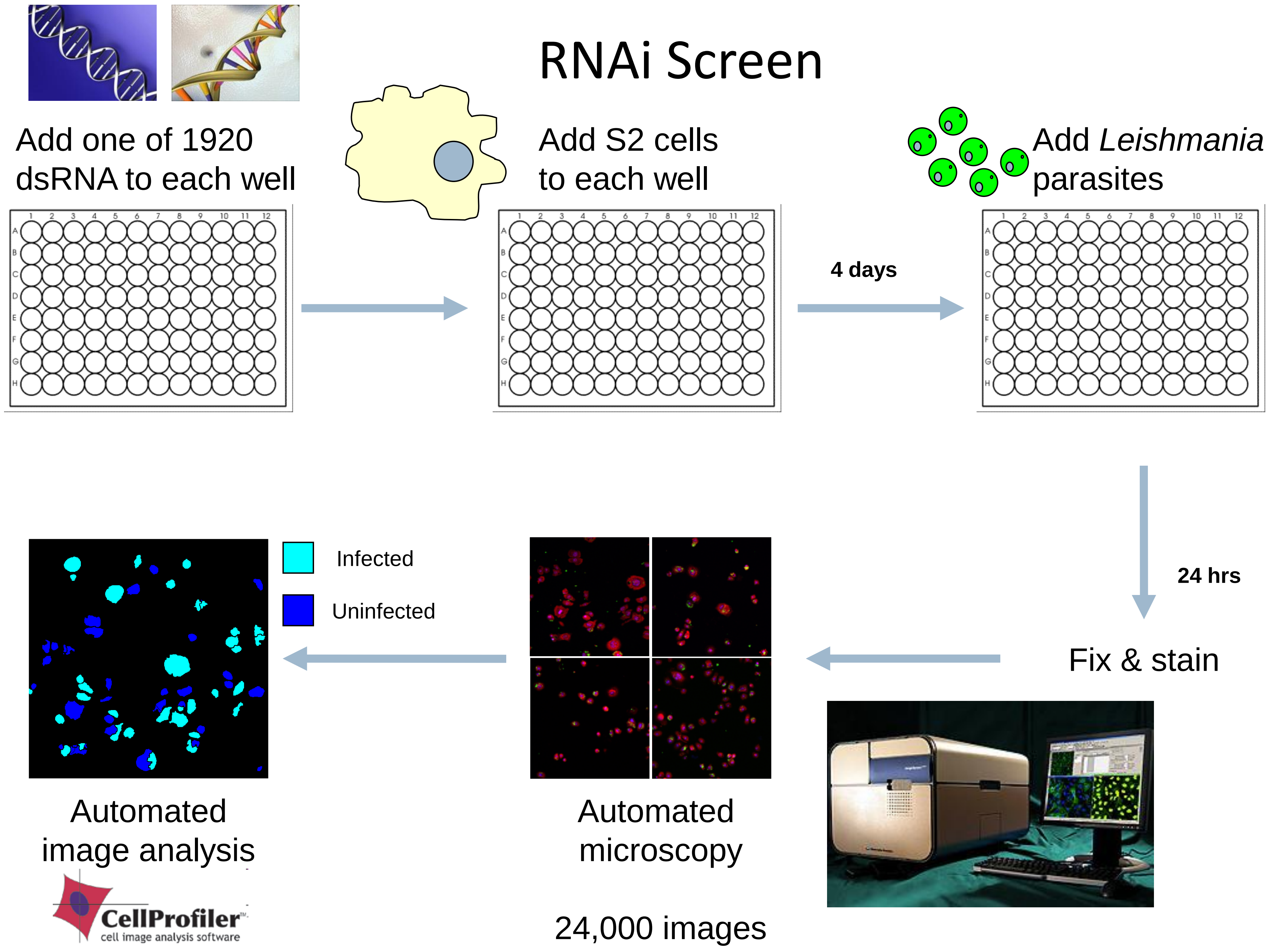
RNAi Screen
Add one of 1920 dsRNA to each well
Add S2 cells to each well
Add Leishmania parasites
4 days
24 hrs
Fix & stain
Infected
Uninfected
Automated image analysis
CellProfiler
cell image analysis software
Automated microscopy
24,000 images
