# Supplementary material for: Identification of Drosophila Gene Products Required for Phagocytosis of Leishmania donovani
Source: PLoS One. 2012 Dec 13;7(12):e51831. doi: 10.1371/journal.pone.0051831 (PMC3521716; doi:10.1371/journal.pone.0051831)
Supplement: Data S3 — Quantitation of ARL8 protein levels after RNAi mediated knock-down was used to determine optimal time period for maximum protein reduction. (PDF) [file pone.0051831.s003.pdf]

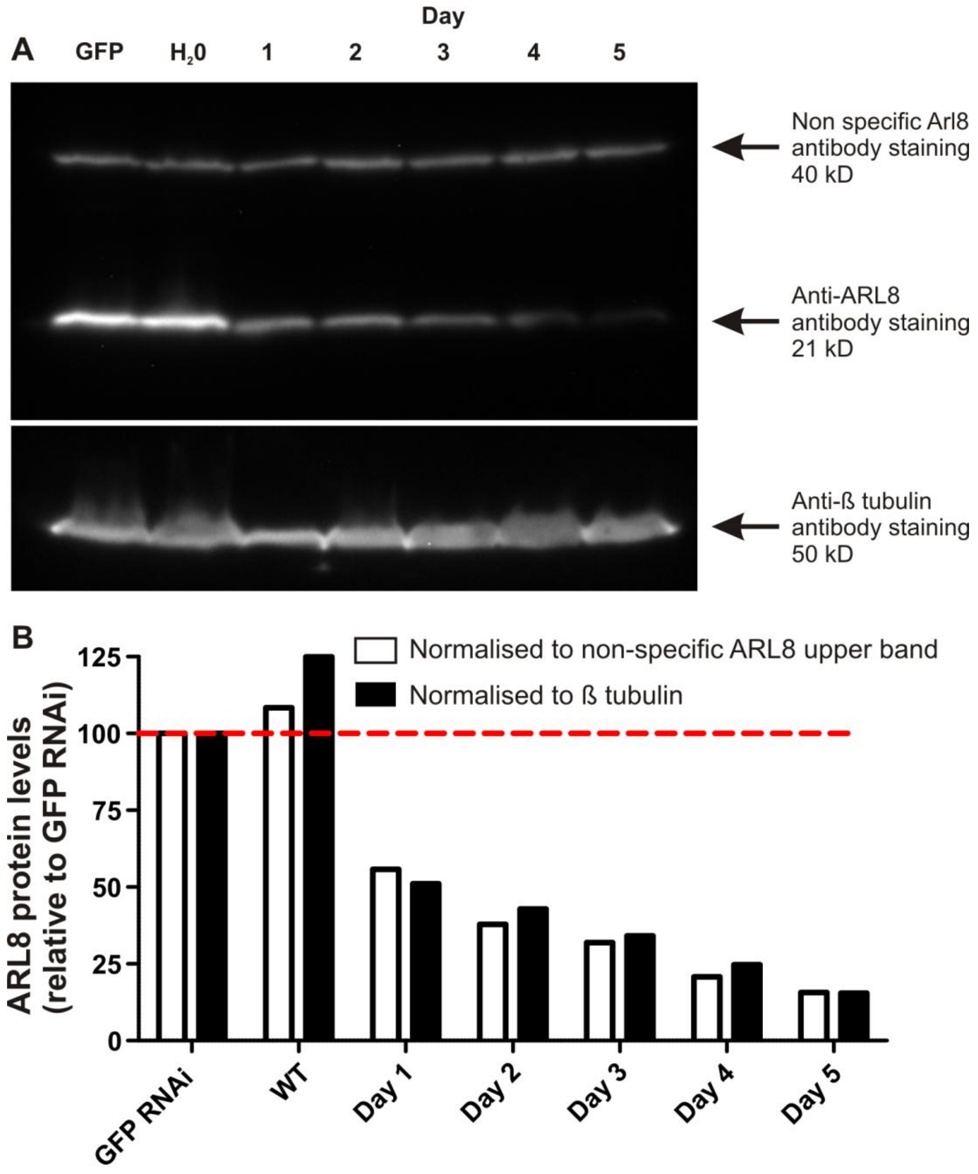

Supplementary Data 3: Quantitation of ARL8 protein levels after RNAi mediated knock-down. **A:** Cells were treated with dsRNA targeting ARL8 for 1 – 5 days and cell lysates analysed by Western blotting. Cells were also treated with a control dsRNA against GFP or no RNAi (H_2O). A larger non-specific protein recognised by the ARL8 antibody and reprobing against β tubulin were used as independent internal controls for protein loading. **B:** Quantitation of band intensities allowed relative protein levels to be calculated.
