# Supplementary material for: Identification of Drosophila Gene Products Required for Phagocytosis of Leishmania donovani
Source: PLoS One. 2012 Dec 13;7(12):e51831. doi: 10.1371/journal.pone.0051831 (PMC3521716; doi:10.1371/journal.pone.0051831)
Supplement: Data S5 — Infection rates after each dsRNA treatment. (PDF) [file pone.0051831.s005.pdf]

Well	CG number	Name	Number of S2 cells	Number of parasites	Number of uninfected cells	Number of infected cells	**Percentage infected**
plate14-H06	CG4038	CG4038	17	18	13	4	**23.53**
plate13-H11	CG3876	CG3876	4	2	3	1	**25.00**
plate16-H07	CG4383	CG33960	35	64	24	11	**31.43**
plate17-H11	CG4568	fuzzy onions	6	40	4	2	**33.33**
plate13-H03	CG3861	knockdown	41	48	27	14	**34.15**
plate10-C04	CG3208	RhoGAP5A	68	95	44	24	**35.29**
plate9-B10	CG3021	CG3021	44	36	28	16	**36.36**
plate12-H05	CG3692	Calpain C	27	69	16	11	**40.74**
plate3-F11	CG1632	CG1632	137	361	74	63	**45.99**
plate5-C10	CG1941	CG1941	306	735	150	156	**50.98**
plate11-E12	CG18408	CAP	94	522	45	49	**52.13**
plate3-E11	CG1607	CG1607	332	788	157	175	**52.71**
plate17-G10	CG4551	smell impaired 35A	44	82	20	24	**54.55**
plate4-E05	CG1785	CG1785	329	680	148	181	**55.02**
plate1-B04	CG1059	Karyopherin beta 3	96	406	43	53	**55.21**
plate11-G12	CG3494	CG3494	50	151	22	28	**56.00**
plate13-H01	CG3853	Glucose transporter type 3	41	76	18	23	**56.10**
plate15-F07	CG4162	lace	84	161	36	48	**57.14**
plate3-F10	CG1630	Inositol 1,4,5-triphosphate kinase 2	19	80	8	11	**57.89**
plate18-H12	CG4721	CG4721	19	55	8	11	**57.89**
plate2-H10	CG1475	Ribosomal protein L13A	183	451	77	106	**57.92**
plate15-D04	CG4111	Ribosomal protein L35	61	243	25	36	**59.02**
plate11-G08	CG30414	CG30414	543	832	220	323	**59.48**
plate7-D12	CG2577	CG2577	430	794	173	257	**59.77**
plate11-B08	CG3367	CG42340	234	474	94	140	**59.83**
plate1-D11	CG1112	alpha-Esterase-7	217	436	87	130	**59.91**
plate5-H12	CG2076	CG2076	264	614	105	159	**60.23**
plate14-D06	CG3956	snail	399	837	157	242	**60.65**
plate15-A04	CG4053	CG4053	41	305	16	25	**60.98**
plate6-A06	CG2086	draper	72	128	28	44	**61.11**

plate7-B06	CG31484	CG31484	178	397	69	109	**61.24**
plate7-H11	CG31973	CG31973	524	848	202	322	**61.45**
plate3-C11	CG1546	prolyl-4-hydroxylase-alpha SG2	143	506	55	88	**61.54**
plate18-H08	CG4713	lethal (2) giant discs 1	13	33	5	8	**61.54**
plate5-G03	CG2028	Casein kinase Ialpha	60	240	23	37	**61.67**
plate4-H08	CG1859	Spn43Ad	168	343	64	104	**61.90**
plate20-C08	CG4931	specifically Rac1-associated protein 1	239	601	91	148	**61.92**
plate15-C12	CG4105	Cytochrome P450-4e3	61	219	23	38	**62.30**
plate5-D09	CG1963	pterin-4a-carbinolamine dehydratase	308	694	116	192	**62.34**
plate18-B11	CG4602	Srp54	40	81	15	25	**62.50**
plate14-G09	CG4021	CG4021	447	844	167	280	**62.64**
plate1-F09	CG1179	Lysozyme B	303	605	113	190	**62.71**
plate7-C12	CG2512	alpha-Tubulin at 84D	421	953	156	265	**62.95**
plate6-G09	CG2238	Elongation factor 2b	281	508	104	177	**62.99**
plate6-E12	CG2179	Xe7	500	915	185	315	**63.00**
plate9-C09	CG3036	CG3036	87	147	32	55	**63.22**
plate3-B05	CG1515	lethal (1) G0155	253	687	93	160	**63.24**
plate9-C08	CG3035	carmine	445	769	163	282	**63.37**
plate11-B12	CG3380	Organic anion transporting polypeptide 58Dc	52	217	19	33	**63.46**
plate9-G05	CG3119	CG3119	1417	2273	517	900	**63.51**
plate11-F12	CG3477	Peroxidase	280	778	102	178	**63.57**
plate1-B11	CG1071	E2F transcription factor 2	88	309	32	56	**63.64**
plate9-E11	CG3085	CG3085	231	475	84	147	**63.64**
plate2-A09	CG1299	CG1299	99	247	36	63	**63.64**
plate11-G03	CG3480	Myb-interacting protein 130	265	777	96	169	**63.77**
plate11-C08	CG3397	CG3397	388	798	140	248	**63.92**
plate14-H04	CG4035	Eukaryotic initiation factor 4E	89	226	32	57	**64.04**
plate13-G01	CG3827	scute	64	155	23	41	**64.06**
plate17-H08	CG4563	CG4563	28	113	10	18	**64.29**
plate20-A12	CG4904	Proteasome 35kD subunit	14	68	5	9	**64.29**
plate4-D06	CG1764	CG1764	374	630	133	241	**64.44**
plate9-H11	CG3156	CG3156	377	703	134	243	**64.46**
plate7-A09	CG2292	CG2292	127	273	45	82	**64.57**

plate6-F11	CG2201	CG2201	302	527	107	195	**64.57**
plate7-B09	CG2381	Syt7	195	429	69	126	**64.62**
plate6-C03	CG2113	CG2113	164	317	58	106	**64.63**
plate2-H07	CG1472	sec24	145	301	51	94	**64.83**
plate9-G07	CG3123	CG3123	169	322	59	110	**65.09**
plate6-H11	CG2261	CstF-50	336	754	117	219	**65.18**
plate9-F11	CG3106	CG3106	1227	2536	427	800	**65.20**
plate6-H06	CG2256	CG2256	253	509	88	165	**65.22**
plate19-A10	CG4738	CG4738	23	47	8	15	**65.22**
plate11-E11	CG3446	CG3446	328	809	114	214	**65.24**
plate3-E12	CG1609	Gcn2	280	608	97	183	**65.36**
plate4-G06	CG1829	Cyp6v1	295	695	102	193	**65.42**
plate16-D11	CG4306	CG4306	272	701	94	178	**65.44**
plate2-G10	CG1454	wings down	612	1438	211	401	**65.52**
plate7-G08	CG2698	CG2698	526	1004	181	345	**65.59**
plate6-H03	CG2252	female sterile (1) homeotic	259	525	89	170	**65.64**
plate4-G08	CG1836	Rad23	155	394	53	102	**65.81**
plate17-H05	CG4560	Arpc3A	41	94	14	27	**65.85**
plate1-E04	CG1126	CG1126	1204	2448	411	793	**65.86**
plate10-F05	CG3277	CG3277	645	1145	220	425	**65.89**
plate4-D12	CG1775	Medea	405	778	138	267	**65.93**
plate3-B11	CG1522	cacophony	229	767	78	151	**65.94**
plate7-D11	CG2574	CG2574	1061	1926	361	700	**65.98**
plate10-H10	CG3325	spindle B	427	867	145	282	**66.04**
plate7-B12	CG2412	Rad51C	731	1222	248	483	**66.07**
plate7-E12	CG31473	CG31473	1330	2544	451	879	**66.09**
plate2-F12	CG1434	CG1434	348	714	118	230	**66.09**
plate13-A05	CG3707	wings apart-like	65	167	22	43	**66.15**
plate9-E03	CG3061	CG3061	956	1661	323	633	**66.21**
plate4-D11	CG1774	CG1774	154	332	52	102	**66.23**
plate9-E05	CG3066	Serine protease 7	1096	2017	370	726	**66.24**
plate3-D11	CG1583	CG1583	489	1538	165	324	**66.26**
plate8-H12	CG2988	empty spiracles	270	651	91	179	**66.30**

plate15-E02	CG4129	lethal (1) G0045	428	970	144	284	**66.36**
plate9-B11	CG3022	metabotropic GABA-B receptor subtype 3	547	1239	184	363	**66.36**
plate18-D09	CG4636	SCAR	461	961	155	306	**66.38**
plate11-F10	CG3473	CG3473	474	1154	159	315	**66.46**
plate9-D10	CG3056	CG3056	717	1257	240	477	**66.53**
plate9-F08	CG32697	lethal (1) G0232	643	1185	215	428	**66.56**
plate9-A08	CG3002	Gga	180	309	60	120	**66.67**
plate9-G02	CG3109	mitochondrial ribosomal protein L16	381	846	127	254	**66.67**
plate8-C11	CG32683	CG32683	81	160	27	54	**66.67**
plate10-H04	CG3314	Ribosomal protein L7A	36	87	12	24	**66.67**
plate11-H11	CG3510	Cyclin B	15	55	5	10	**66.67**
plate12-H04	CG3690	CG3690	45	108	15	30	**66.67**
plate14-A01	CG3879	Multi drug resistance 49	138	466	46	92	**66.67**
plate15-A11	CG4065	CG4065	15	47	5	10	**66.67**
plate15-F12	CG4167	Heat shock gene 67Ba	6	24	2	4	**66.67**
plate16-C12	CG4280	croquemort	24	60	8	16	**66.67**
plate17-A12	CG4412	ATPase coupling factor 6	3	15	1	2	**66.67**
plate18-F02	CG4662	CG4662	273	659	91	182	**66.67**
plate10-F12	CG3285	CG3285	638	1153	212	426	**66.77**
plate5-B05	CG1901	maverick	304	622	101	203	**66.78**
plate3-G11	CG1650	unplugged	274	694	91	183	**66.79**
plate1-F12	CG1200	APP-like protein interacting protein 1	253	690	84	169	**66.80**
plate14-F12	CG4007	Neurospecific receptor kinase	166	470	55	111	**66.87**
plate4-F12	CG1818	Updo	142	271	47	95	**66.90**
plate4-H02	CG1845	CG1845	378	825	125	253	**66.93**
plate11-F11	CG3476	CG3476	367	856	121	246	**67.03**
plate19-B07	CG4755	RhoGAP92B	562	1183	185	377	**67.08**
plate13-C04	CG3744	CG3744	705	1362	232	473	**67.09**
plate1-G12	CG1236	CG1236	210	675	69	141	**67.14**
plate7-C04	CG2471	Sclp	192	396	63	129	**67.19**
plate9-H06	CG3140	Adenylate kinase-2	561	1231	184	377	**67.20**
plate1-E09	CG1132	forkhead domain 64A	235	446	77	158	**67.23**
plate4-E12	CG1795	Ogg1	397	738	130	267	**67.25**

plate10-H03	CG3313	CG3313	281	594	92	189	**67.26**
plate1-D07	CG1106	Gelsolin	422	1035	138	284	**67.30**
plate5-E11	CG1986	CG1986	98	329	32	66	**67.35**
plate11-A06	CG3337	CG3337	138	393	45	93	**67.39**
plate20-H10	CG5030	CG34139	730	1936	238	492	**67.40**
plate8-E12	CG2924	CG2924	583	1410	190	393	**67.41**
plate8-F11	CG2944	-	381	838	124	257	**67.45**
plate5-C07	CG1937	septin interacting protein 3	378	934	123	255	**67.46**
plate7-F07	CG2671	lethal (2) giant larvae	163	325	53	110	**67.48**
plate2-F09	CG1429	Myocyte enhancing factor 2	114	326	37	77	**67.54**
plate13-B10	CG3734	CG3734	721	1334	234	487	**67.55**
plate4-F11	CG1817	Protein tyrosine phosphatase 10D	151	410	49	102	**67.55**
plate15-H09	CG4205	Ferredoxin	151	405	49	102	**67.55**
plate6-C08	CG2121	CG2121	262	564	85	177	**67.56**
plate11-D12	CG3424	pathetic	139	748	45	94	**67.63**
plate11-D05	CG3412	supernumerary limbs	476	1019	154	322	**67.65**
plate19-E04	CG4813	CG4813	439	1124	142	297	**67.65**
plate13-E11	CG3798	N-methyl-D-aspartate receptor-associated protein	402	878	130	272	**67.66**
plate10-B08	CG3189	DNA polymerase interacting tpr containing protein of 47kD	322	799	104	218	**67.70**
plate4-H10	CG1862	Ephrin	480	1044	155	325	**67.71**
plate15-B12	CG4086	Suppressor of ref(2)P sterility	183	602	59	124	**67.76**
plate7-E06	CG2615	IkappaB kinase-like 2	469	1005	151	318	**67.80**
plate1-D12	CG1114	HIF prolyl hydroxylase	292	670	94	198	**67.81**
plate11-C11	CG3401	beta-Tubulin at 60D	336	730	108	228	**67.86**
plate13-H05	CG3864	thioredoxin-2	28	116	9	19	**67.86**
plate9-B08	CG3019	suppressor of white-apricot	417	902	134	283	**67.87**
plate2-G12	CG1461	CG1461	218	418	70	148	**67.89**
plate11-E08	CG3437	CG3437	405	759	130	275	**67.90**
plate1-C07	CG1086	Glucose transporter 1	237	600	76	161	**67.93**
plate2-A03	CG1274	thioredoxin peroxidase 2	237	619	76	161	**67.93**
plate6-C04	CG2114	Fmrf Receptor	103	236	33	70	**67.96**
plate6-H02	CG2248	Rac1	153	346	49	104	**67.97**
plate4-C10	CG1750	CG1750	422	1395	135	287	**68.01**

plate9-H10	CG3152	Trap1	216	581	69	147	**68.06**
plate7-F06	CG2670	TBP-associated factor 7	166	412	53	113	**68.07**
plate2-D03	CG1372	yolkless	69	191	22	47	**68.12**
plate4-E11	CG1794	Matrix metalloproteinase 2	414	1062	132	282	**68.12**
plate10-E08	CG3263	cAMP-dependent protein kinase R1	662	1255	211	451	**68.13**
plate9-G09	CG3126	C3G	797	1645	254	543	**68.13**
plate6-A11	CG2093	CG2093	110	214	35	75	**68.18**
plate17-G12	CG4554	CG4554	22	54	7	15	**68.18**
plate7-D01	CG2520	like-AP180	478	894	152	326	**68.20**
plate19-F12	CG4841	CG4841	623	1456	198	425	**68.22**
plate9-C07	CG3034	Mediator complex subunit 22	384	832	122	262	**68.23**
plate11-D09	CG3421	RhoGAP93B	318	788	101	217	**68.24**
plate10-C11	CG3220	Muscle LIM protein at 60A	739	1278	234	505	**68.34**
plate8-H06	CG2981	Troponin C at 41C	373	761	118	255	**68.36**
plate2-E09	CG1410	waclaw	228	534	72	156	**68.42**
plate16-A12	CG4237	GTPase-activating protein 69C	19	56	6	13	**68.42**
plate20-H11	CG5032	adrift	19	69	6	13	**68.42**
plate7-B11	CG2411	patched	447	932	141	306	**68.46**
plate8-H08	CG2983	CG2983	1075	2294	339	736	**68.47**
plate7-E08	CG2621	shaggy	333	652	105	228	**68.47**
plate12-B12	CG3572	visceral mesodermal armadillo-repeats	146	358	46	100	**68.49**
plate2-B09	CG1321	shaking B	54	162	17	37	**68.52**
plate8-B11	CG2849	Ras-related protein	267	563	84	183	**68.54**
plate9-F10	CG3105	CG3105	302	673	95	207	**68.54**
plate9-H08	CG3149	CG3149	328	649	103	225	**68.60**
plate1-G10	CG1233	CG1233	188	564	59	129	**68.62**
plate7-E11	CG2647	period	1246	2395	391	855	**68.62**
plate7-D10	CG32654	CG32654	612	1211	192	420	**68.63**
plate19-H12	CG4886	cyclophilin-33	102	326	32	70	**68.63**
plate9-E10	CG3075	CG3075	697	1425	218	479	**68.72**
plate2-B03	CG1311	CG1311	461	982	144	317	**68.76**
plate4-B12	CG1732	CG1732	365	759	114	251	**68.77**
plate9-D12	CG3058	Dim1	285	664	89	196	**68.77**

plate6-A03	CG2079	Downstream of kinase	327	659	102	225	**68.81**
plate7-F12	CG2679	goliath	1036	2099	322	714	**68.92**
plate11-A08	CG3340	Kruppel	428	1033	133	295	**68.93**
plate11-H08	CG3505	CG3505	412	915	128	284	**68.93**
plate10-C07	CG3212	-	293	693	91	202	**68.94**
plate5-F12	CG2019	dispatched	596	1395	185	411	**68.96**
plate14-F11	CG4006	Akt1	203	478	63	140	**68.97**
plate14-C11	CG3943	kraken	316	793	98	218	**68.99**
plate2-A10	CG1303	O-6-alkylguanine-DNA alkyltransferase	1077	2221	334	743	**68.99**
plate15-B11	CG4084	lethal (2) neighbor of tid	287	633	89	198	**68.99**
plate6-E11	CG2177	CG2177	300	524	93	207	**69.00**
plate4-E08	CG1791	CG1791	484	1062	150	334	**69.01**
plate14-H01	CG4030	CG4030	113	329	35	78	**69.03**
plate7-E10	CG2641	CG2641	682	1460	211	471	**69.06**
plate11-H07	CG3504	inactivation no afterpotential D	262	559	81	181	**69.08**
plate19-F01	CG4825	CG4825	220	594	68	152	**69.09**
plate8-H01	CG2975	CG2975	1774	3398	548	1226	**69.11**
plate9-D11	CG3057	congested-like trachea	761	1730	235	526	**69.12**
plate1-G09	CG1228	Ptpmeg	298	724	92	206	**69.13**
plate12-D05	CG3613	quaking related 58E-1	367	853	113	254	**69.21**
plate14-G12	CG4027	Actin 5C	13	43	4	9	**69.23**
plate15-H06	CG4202	Sas10	39	87	12	27	**69.23**
plate17-E06	CG4494	smt3	78	284	24	54	**69.23**
plate18-F11	CG4675	Na[+]-driven anion exchanger 1	130	282	40	90	**69.23**
plate4-D05	CG1763	no distributive disjunction	322	762	99	223	**69.25**
plate13-C11	CG3758	escargot	192	528	59	133	**69.27**
plate4-C01	CG1736	Prosalpha3T	316	774	97	219	**69.30**
plate4-B03	CG33071	CG33714	75	208	23	52	**69.33**
plate5-C09	CG1939	CG1939	441	1197	135	306	**69.39**
plate11-G06	CG3484	Adh-related	147	372	45	102	**69.39**
plate19-A09	CG4735	shutdown	245	644	75	170	**69.39**
plate13-E10	CG3797	CG3797	941	1940	288	653	**69.39**
plate8-F10	CG2943	CG2943	556	1175	170	386	**69.42**

plate15-A02	CG4050	CG4050	72	330	22	50	**69.44**
plate7-H09	CG2747	CG2747	419	857	128	291	**69.45**
plate11-E09	CG3443	pecanex	213	468	65	148	**69.48**
plate20-G12	CG5017	CG5017	282	786	86	196	**69.50**
plate3-H12	CG1676	cactin	774	2004	236	538	**69.51**
plate10-H11	CG3326	CG3326	674	1219	205	469	**69.58**
plate10-H09	CG3324	cGMP-dependent protein kinase 21D	306	728	93	213	**69.61**
plate17-C10	CG4452	CG4452	102	237	31	71	**69.61**
plate8-C12	CG2885	RabX2	385	796	117	268	**69.61**
plate1-F10	CG1180	Lysozyme E	316	769	96	220	**69.62**
plate9-F02	CG3090	Sox box protein 14	303	696	92	211	**69.64**
plate7-C11	CG31547	CG31547	359	744	109	250	**69.64**
plate6-E09	CG2171	Triose phosphate isomerase	145	342	44	101	**69.66**
plate9-H09	CG3151	RNA-binding protein 9	290	679	88	202	**69.66**
plate14-E09	CG3981	Unc-76	689	1389	209	480	**69.67**
plate2-A11	CG1304	CG1304	122	262	37	85	**69.67**
plate6-C11	CG2125	cubitus interruptus	254	488	77	177	**69.69**
plate12-G10	CG3678	CG3678	574	1251	174	400	**69.69**
plate7-A04	CG2275	Jun-related antigen	198	525	60	138	**69.70**
plate9-B05	CG3016	CG3016	327	736	99	228	**69.72**
plate1-E01	CG1116	CG1116	598	1418	181	417	**69.73**
plate14-H08	CG4040	CG4040	129	317	39	90	**69.77**
plate14-G10	CG33138	CG33138	354	958	107	247	**69.77**
plate13-D10	CG3779	numb	417	857	126	291	**69.78**
plate14-A07	CG3889	COP9 complex homolog subunit 1 b	53	162	16	37	**69.81**
plate7-E05	CG2614	CG2614	646	1410	195	451	**69.81**
plate9-C11	CG3038	CG3038	358	872	108	250	**69.83**
plate6-D07	CG32687	CG32687	63	214	19	44	**69.84**
plate13-H08	CG3871	Six4	93	244	28	65	**69.89**
plate9-D05	CG3045	CG3045	765	1642	230	535	**69.93**
plate1-F11	CG1193	CG1193	336	716	101	235	**69.94**
plate15-D11	CG4123	Multiple inositol polyphosphate phosphatase 1	509	1298	153	356	**69.94**
plate9-G10	CG3127	Phosphoglycerate kinase	533	1224	160	373	**69.98**

plate3-H05	CG1664	small bristles	10	49	3	7	**70.00**
plate6-E04	CG2160	Suppressor of Cytokine Signaling at 44A	420	905	126	294	**70.00**
plate12-H12	CG3702	CG3702	40	111	12	28	**70.00**
plate13-G12	CG3851	odd skipped	60	122	18	42	**70.00**
plate15-G04	CG4173	Septin-2	10	90	3	7	**70.00**
plate17-B11	CG4434	CG4434	100	306	30	70	**70.00**
plate6-G02	CG2210	abnormal wing discs	507	1100	152	355	**70.02**
plate3-D12	CG1584	Origin recognition complex subunit 6	257	685	77	180	**70.04**
plate12-E11	CG3641	CG33332	484	996	145	339	**70.04**
plate7-B04	CG2330	Neurochondrin	197	506	59	138	**70.05**
plate13-E09	CG3796	achaete	528	1347	158	370	**70.08**
plate12-B11	CG3571	CG3571	752	1570	225	527	**70.08**
plate15-B02	CG4069	CG4069	117	300	35	82	**70.09**
plate5-H11	CG2072	TXBP181-like	291	891	87	204	**70.10**
plate8-B12	CG2851	Goosecoid	281	770	84	197	**70.11**
plate15-F03	CG4157	Rpn12	87	234	26	61	**70.11**
plate14-G06	CG4017	CG4017	512	1278	153	359	**70.12**
plate19-B11	CG4759	Ribosomal protein L27	251	725	75	176	**70.12**
plate15-E01	CG4125	roughest	432	1043	129	303	**70.14**
plate18-C01	CG4607	CG4607	67	205	20	47	**70.15**
plate18-C11	CG4622	CG4622	134	334	40	94	**70.15**
plate8-F08	CG2938	CG2938	798	1782	238	560	**70.18**
plate3-A11	CG1505	gastrulation-defective	369	998	110	259	**70.19**
plate10-B07	CG3187	Sirt4	292	637	87	205	**70.21**
plate5-G12	CG2054	Chitinase 2	329	860	98	231	**70.21**
plate4-G01	CG1819	CG34120	131	405	39	92	**70.23**
plate15-C11	CG4104	Trehalose-6-phosphate synthase 1	353	922	105	248	**70.25**
plate9-B09	CG3020	CG42333	269	593	80	189	**70.26**
plate18-B08	CG4598	CG4598	380	1009	113	267	**70.26**
plate12-E08	CG3637	Cortactin	680	660	182	478	**70.29**
plate4-C09	CG1749	CG1749	303	882	90	213	**70.30**
plate18-E09	CG4654	DP transcription factor	165	505	49	116	**70.30**
plate14-H02	CG4032	Abl tyrosine kinase	192	534	57	135	**70.31**

plate13-C02	CG3739	CG3739	411	840	122	289	**70.32**
plate4-A11	CG1697	rhomboid-4	509	1333	151	358	**70.33**
plate18-E11	CG4658	CG4658	81	179	24	57	**70.37**
plate18-E12	CG4659	Signal recognition particle protein 54k	27	88	8	19	**70.37**
plate9-A11	CG3006	Flavin-containing monooxygenase 1	871	1807	258	613	**70.38**
plate9-E09	CG3074	CG3074	439	1010	130	309	**70.39**
plate7-H12	CG2767	CG2767	179	557	53	126	**70.39**
plate19-E10	CG4822	CG4822	473	1050	140	333	**70.40**
plate9-B03	CG3013		257	534	76	181	**70.43**
plate12-G02	CG3663	CG3663	832	1855	246	586	**70.43**
plate20-C12	CG4936	CG4936	115	335	34	81	**70.43**
plate7-H03	CG2714	deformed wings	237	483	70	167	**70.46**
plate6-F04	CG2185	CG2185	657	1262	194	463	**70.47**
plate12-A11	CG3534	CG3534	472	1149	139	333	**70.55**
plate13-G06	CG3842	CG3842	214	543	63	151	**70.56**
plate14-D11	CG3962	Keap1	445	1003	131	314	**70.56**
plate1-F06	CG1158	Tim17b1	286	719	84	202	**70.63**
plate9-F09	CG3104	CG3104	555	1097	163	392	**70.63**
plate1-C11	CG1091	CG1091	344	865	101	243	**70.64**
plate19-B03	CG4750	loopin-1	109	311	32	77	**70.64**
plate17-C06	CG4447	CG4447	201	469	59	142	**70.65**
plate2-H11	CG1479	bent	443	990	130	313	**70.65**
plate20-D09	CG4950	CG4950	809	1962	237	572	**70.70**
plate1-F08	CG1167	Ras oncogene at 64B	403	864	118	285	**70.72**
plate14-F04	CG3992	serpent	417	904	122	295	**70.74**
plate9-D08	CG3051	SNF1A/AMP-activated protein kinase	253	524	74	179	**70.75**
plate19-H11	CG4881	spalt-related	342	979	100	242	**70.76**
plate8-E10	CG2921	CG2921	373	933	109	264	**70.78**
plate6-H05	CG2254	CG2254	486	1075	142	344	**70.78**
plate4-F07	CG1810	mRNA-capping-enzyme	305	971	89	216	**70.82**
plate2-G04	CG1442	eIF4E-6	329	710	96	233	**70.82**
plate2-E10	CG1411	Collapsin Response Mediator Protein	809	1847	236	573	**70.83**
plate2-C09	CG1349	dj-1beta	175	453	51	124	**70.86**

plate7-D05	CG32671	Rab GTPase 9Fa	635	1298	185	450	**70.87**
plate4-H11	CG1864	Hormone receptor-like in 38	206	576	60	146	**70.87**
plate5-H05	CG2062	Cytochrome P450-4e1	261	703	76	185	**70.88**
plate12-F11	CG3656	Cytochrome P450-4d1	691	1451	201	490	**70.91**
plate7-G10	CG2701	CG2701	258	559	75	183	**70.93**
plate1-D03	CG1100	Rpn5	117	449	34	83	**70.94**
plate12-D11	CG3625	CG3625	272	693	79	193	**70.96**
plate8-H05	CG2980	thoc5	224	635	65	159	**70.98**
plate6-H10	CG2260	CG2260	193	447	56	137	**70.98**
plate14-F10	CG4003	pontin	162	459	47	115	**70.99**
plate7-G12	CG2711	deformed wings	586	1135	170	416	**70.99**
plate10-H12	CG3328	CG3328	576	1247	167	409	**71.01**
plate10-H08	CG3322	Laminin B2	276	669	80	196	**71.01**
plate14-E10	CG3983	CG3983	138	386	40	98	**71.01**
plate4-C12	CG1753	CG1753	442	939	128	314	**71.04**
plate18-C09	CG4618	CG4618	114	305	33	81	**71.05**
plate9-C01	CG3025	males absent on the first	826	1804	239	587	**71.07**
plate17-E11	CG4502	CG4502	671	1684	194	477	**71.09**
plate20-A05	CG4896	CG4896	246	634	71	175	**71.14**
plate17-B12	CG4435	FucTB	52	135	15	37	**71.15**
plate2-G07	CG1449	Zn finger homeodomain 2	319	714	92	227	**71.16**
plate17-F11	CG4533	lethal (2) essential for life	288	795	83	205	**71.18**
plate9-A10	CG3004	CG3004	531	1336	153	378	**71.19**
plate14-C10	CG3942	CG3942	354	840	102	252	**71.19**
plate4-G07	CG1832	CG1832	198	499	57	141	**71.21**
plate7-C10	CG2508	cdc23	153	371	44	109	**71.24**
plate2-B11	CG32251	CG32251	320	726	92	228	**71.25**
plate10-G08	CG3299	Vinculin	456	955	131	325	**71.27**
plate15-F01	CG4154	Guanylyl cyclase at 88E	338	771	97	241	**71.30**
plate11-F06	CG3460	Nonsense-mediated mRNA 3	157	403	45	112	**71.34**
plate1-G08	CG1227	CG1227	527	1198	151	376	**71.35**
plate11-G11	CG3493	CG3493	241	749	69	172	**71.37**
plate7-A11	CG2304	Trc8	283	639	81	202	**71.38**

plate11-C02	CG3385	nervy	521	1295	149	372	**71.40**
plate3-E10	CG1605	az2	21	69	6	15	**71.43**
plate4-A08	CG1693	tweety	224	556	64	160	**71.43**
plate4-C06	CG1746	CG1746	28	93	8	20	**71.43**
plate7-F10	CG2677	eIF2B-beta	420	1173	120	300	**71.43**
plate16-E06	CG4320	raptor	91	243	26	65	**71.43**
plate17-D12	CG4482	moladietz	14	38	4	10	**71.43**
plate20-H12	CG5033	CG5033	126	438	36	90	**71.43**
plate9-F06	CG3099	CG3099	294	674	84	210	**71.43**
plate9-H03	CG3135	shifted	578	1221	165	413	**71.45**
plate7-H05	CG2720	Hsp70/Hsp90 organizing protein homolog	494	1190	141	353	**71.46**
plate18-B09	CG4599	Tetratricopeptide repeat protein 2	459	1170	131	328	**71.46**
plate7-F09	CG2675	Csat	403	849	115	288	**71.46**
plate6-G08	CG2229	Jonah 99Fii	291	618	83	208	**71.48**
plate8-A07	CG2790	CG2790	491	1089	140	351	**71.49**
plate1-D09	CG1108	alpha-Esterase-6	242	516	69	173	**71.49**
plate11-D06	CG3415	CG3415	235	556	67	168	**71.49**
plate15-D05	CG4114	expanded	684	1532	195	489	**71.49**
plate15-B03	CG4070	Tis11 homolog	207	546	59	148	**71.50**
plate10-G09	CG3301	CG3301	674	1409	192	482	**71.51**
plate7-F11	CG2678	CG2678	488	1010	139	349	**71.52**
plate9-E08	CG3073	lethal (1) G0144	281	636	80	201	**71.53**
plate5-B07	CG32662	CG32662	109	313	31	78	**71.56**
plate14-D02	CG3948	zetaCOP	218	607	62	156	**71.56**
plate12-A10	CG3532	CG3532	331	767	94	237	**71.60**
plate7-G01	CG2680	CG2680	843	1578	239	604	**71.65**
plate14-D09	CG3960	-	642	1378	182	460	**71.65**
plate9-E12	CG3086	MAP kinase activated protein-kinase-2	819	1691	232	587	**71.67**
plate7-D07	CG2534	canoe	805	1636	228	577	**71.68**
plate10-D08	CG3238	CG3238	378	890	107	271	**71.69**
plate10-G04	CG3292	CG3292	106	299	30	76	**71.70**
plate1-A05	CG1021	CG1021	364	1004	103	261	**71.70**
plate12-D10	CG3622	stall	251	552	71	180	**71.71**

plate13-E07	CG3793	CG3793	527	1229	149	378	**71.73**
plate15-G10	CG4184	Mediator complex subunit 15	329	836	93	236	**71.73**
plate2-G08	CG1451	APC-like	92	200	26	66	**71.74**
plate20-C11	CG4935	CG4935	457	1132	129	328	**71.77**
plate15-C03	CG4089	CG4089	287	746	81	206	**71.78**
plate4-B08	CG1718	CG1718	284	807	80	204	**71.83**
plate11-G10	CG3491	CG3491	387	1048	109	278	**71.83**
plate10-C03	CG3204	Ras-associated protein 2-like	451	929	127	324	**71.84**
plate5-A12	CG1891	saxophone	128	475	36	92	**71.88**
plate6-H12	CG2262	Smad on X	288	636	81	207	**71.88**
plate14-A06	CG3887	CG3887	96	294	27	69	**71.88**
plate4-H07	CG1855	CG11138	381	920	107	274	**71.92**
plate16-G11	CG4371	Glutathione S transferase D7	82	257	23	59	**71.95**
plate17-G06	CG4547	Ataxin 1	132	337	37	95	**71.97**
plate19-C08	CG4775	Transport and Golgi organization 14	314	958	88	226	**71.97**
plate8-H07	CG2982	CG2982	728	1569	204	524	**71.98**
plate5-B10	CG1909	CG1909	389	935	109	280	**71.98**
plate1-A07	CG1030	Sex combs reduced	282	624	79	203	**71.99**
plate17-A08	CG4406	CG4406	50	132	14	36	**72.00**
plate1-C02	CG1075	CG1075	736	1646	206	530	**72.01**
plate11-B01	CG3355	CG3355	729	1847	204	525	**72.02**
plate1-H10	CG1257	alpha-Esterase-3	143	366	40	103	**72.03**
plate1-H03	CG1242	Heat shock protein 83	394	1248	110	284	**72.08**
plate9-F12	CG3107	CG3107	412	895	115	297	**72.09**
plate8-B03	CG2822	Shaker cognate w	584	1205	163	421	**72.09**
plate16-E07	CG4321	Cytochrome P450-4d8	129	343	36	93	**72.09**
plate18-C12	CG4623	CG4623	86	279	24	62	**72.09**
plate4-B09	CG1724	CG1724	276	843	77	199	**72.10**
plate2-F07	CG1427	CG1427	509	1231	142	367	**72.10**
plate2-D01	CG30498	boca	233	593	65	168	**72.10**
plate9-B07	CG3018	lesswright	104	326	29	75	**72.12**
plate12-G09	CG3675	Arginine methyltransferase 2	1069	2283	298	771	**72.12**
plate6-G11	CG2244	MTA1-like	524	1045	146	378	**72.14**

plate18-G11	CG4698	Wnt oncogene analog 4	115	314	32	83	**72.17**
plate19-G09	CG4854	CG4854	507	1197	141	366	**72.19**
plate8-F12	CG2945	cinnamon	975	2135	271	704	**72.21**
plate4-A05	CG1689	lozenge	270	729	75	195	**72.22**
plate4-D03	CG32666	CG32666	216	639	60	156	**72.22**
plate13-G11	CG3850	sugarbabe	18	69	5	13	**72.22**
plate4-C08	CG1748	RhoGAP102A	533	1418	148	385	**72.23**
plate1-E12	CG1135	CG1135	137	494	38	99	**72.26**
plate2-G05	CG1444	CG1444	411	967	114	297	**72.26**
plate6-B03	CG2098	ferrochelatase	440	1055	122	318	**72.27**
plate15-G06	CG4179	small conductance calcium-activated potassium channel	660	1516	183	477	**72.27**
plate15-E05	CG4140	CG4140	541	1324	150	391	**72.27**
plate2-G01	CG1438	Cytochrome P450-4c3	693	1458	192	501	**72.29**
plate11-F09	CG3469	beta subunit of type I geranylgeranyl transferase	213	524	59	154	**72.30**
plate10-F04	CG3274	Brahma associated protein 170kD	65	169	18	47	**72.31**
plate15-E09	CG4147	Heat shock protein cognate 3	130	316	36	94	**72.31**
plate2-H08	CG1473	prolyl-4-hydroxylase-alpha EFB	134	293	37	97	**72.39**
plate11-D08	CG3419	CG3419	268	682	74	194	**72.39**
plate8-C01	CG2855	anterior pharynx defective 1	932	1948	257	675	**72.42**
plate13-F10	CG3817	CG3817	283	581	78	205	**72.44**
plate4-G11	CG1839	F box and leucine-rich-repeat gene 4	254	758	70	184	**72.44**
plate19-C09	CG4778	obstructor-B	381	881	105	276	**72.44**
plate4-H05	CG1849	runt	265	600	73	192	**72.45**
plate11-C06	CG31774	friend of echinoid	236	594	65	171	**72.46**
plate7-C06	CG2488	(6-4)-photolyase	534	1154	147	387	**72.47**
plate1-C06	CG1084	Contactin	171	580	47	124	**72.51**
plate13-F12	CG3822	CG3822	182	435	50	132	**72.53**
plate4-E10	CG1793	Mediator complex subunit 26	233	633	64	169	**72.53**
plate2-F05	CG1422	p115	295	677	81	214	**72.54**
plate3-B10	CG32714	CG32714	62	188	17	45	**72.58**
plate14-G11	CG4025	CG4025	624	1295	171	453	**72.60**
plate16-A01	CG4210	CG4210	73	158	20	53	**72.60**
plate5-F03	CG1998	CG1998	230	628	63	167	**72.61**

plate15-B07	CG4079	TBP-associated factor 11	460	1128	126	334	**72.61**
plate6-H09	CG2259	Glutamate-cysteine ligase catalytic subunit	157	433	43	114	**72.61**
plate8-B04	CG2829	Tousled-like kinase	391	923	107	284	**72.63**
plate6-F06	CG2189	Deformed	424	943	116	308	**72.64**
plate8-C06	CG2862	CG2862	212	595	58	154	**72.64**
plate2-F01	CG1416	CG1416	563	1191	154	409	**72.65**
plate10-C12	CG3222	CG3222	589	1508	161	428	**72.67**
plate14-F06	CG3996	CG3996	520	1242	142	378	**72.69**
plate11-B03	CG3358	CG3358	535	1212	146	389	**72.71**
plate17-A11	CG32649	CG32649	44	179	12	32	**72.73**
plate12-H06	CG3695	Mediator complex subunit 23	33	108	9	24	**72.73**
plate17-A02	CG4394	-	165	421	45	120	**72.73**
plate17-D10	CG4477	CG4477	110	305	30	80	**72.73**
plate9-F05	CG31694	CG31694	312	610	85	227	**72.76**
plate12-B09	CG3564	CHOp24	760	1667	207	553	**72.76**
plate14-F07	CG3998	Zinc finger protein 30C	503	1212	137	366	**72.76**
plate5-G07	CG2048	discs overgrown	235	640	64	171	**72.77**
plate17-E05	CG4488	wee	235	555	64	171	**72.77**
plate19-C12	CG4785	CG4785	213	463	58	155	**72.77**
plate12-G06	CG3668	forkhead domain 59A	606	1365	165	441	**72.77**
plate4-E07	CG1789	CG1789	180	479	49	131	**72.78**
plate20-B12	CG4921	Rab-protein 4	147	464	40	107	**72.79**
plate17-D09	CG4476	CG4476	184	535	50	134	**72.83**
plate18-F07	CG4670	CG4670	162	417	44	118	**72.84**
plate6-E01	CG2155	vermilion	722	1489	196	526	**72.85**
plate2-C01	CG1333	Ero1L	619	1490	168	451	**72.86**
plate3-E04	CG1597	CG1597	188	515	51	137	**72.87**
plate19-D01	CG4787	-	225	648	61	164	**72.89**
plate7-F05	CG2669	humpty dumpty	262	664	71	191	**72.90**
plate11-B02	CG3356	CG3356	550	1301	149	401	**72.91**
plate18-F12	CG4676	CG4676	48	223	13	35	**72.92**
plate19-B12	CG4760	boule	506	1373	137	369	**72.92**
plate3-B12	CG1523	CG1523	218	667	59	159	**72.94**

plate4-A10	CG1696	lethal (1) G0269	255	674	69	186	**72.94**
plate20-B02	CG4907	CG4907	122	297	33	89	**72.95**
plate4-F06	CG1809	CG1809	525	1319	142	383	**72.95**
plate14-A11	CG30171	Unc-89	403	887	109	294	**72.95**
plate19-H02	CG4863	Ribosomal protein L3	603	1636	163	440	**72.97**
plate14-E11	CG3985	Synapsin	74	184	18	54	**72.97**
plate10-E09	CG3264	CG3264	441	1096	119	322	**73.02**
plate11-B09	CG3373	Hemomucin	319	822	86	233	**73.04**
plate10-D03	CG3226	CG3226	549	1177	148	401	**73.04**
plate7-A02	CG2264	CG2264	345	726	93	252	**73.04**
plate6-F05	CG2187	CG2187	245	561	66	179	**73.06**
plate4-A06	CG1691	IGF-II mRNA-binding protein	208	531	56	152	**73.08**
plate15-F11	CG4166	non-stop	338	910	91	247	**73.08**
plate15-H01	CG4192	kekkon-3	52	215	14	38	**73.08**
plate18-F10	CG4674	CG4674	197	520	53	144	**73.10**
plate1-H05	CG1245	Mediator complex subunit 27	435	1163	117	318	**73.10**
plate9-G01	CG3108	CG3108	357	802	96	261	**73.11**
plate12-G01	CG3696	kismet	119	462	32	87	**73.11**
plate15-B06	CG4078	CG4078	279	696	75	204	**73.12**
plate5-G05	CG2040	hikaru genki	268	718	72	196	**73.13**
plate11-H12	CG3511	CG3511	67	246	18	49	**73.13**
plate9-C12	CG3039	optic ganglion reduced	41	222	11	30	**73.17**
plate9-H04	CG3136	Atf6	164	436	44	120	**73.17**
plate20-F11	CG4994	Mitochondrial phosphate carrier protein	384	1376	103	281	**73.18**
plate8-A06	CG2789	CG2789	388	826	104	284	**73.20**
plate2-C10	CG1354	CG1354	993	2333	266	727	**73.21**
plate4-E04	CG1783	Slip1	112	268	30	82	**73.21**
plate14-C09	CG3941	pita	351	737	94	257	**73.22**
plate5-H06	CG2063	CG2063	579	1700	155	424	**73.23**
plate3-E01	CG1587	Crk	228	654	61	167	**73.25**
plate12-H09	CG3699	CG3699	501	1343	134	367	**73.25**
plate2-G03	CG1441	CG1441	617	1638	165	452	**73.26**
plate11-A01	CG3329	Proteasome beta2 subunit	217	641	58	159	**73.27**

plate12-F02	CG3645	CG3645	610	1353	163	447	**73.28**
plate7-B05	CG2331	TER94	176	531	47	129	**73.30**
plate14-C07	CG3939	CG3939	251	553	67	184	**73.31**
plate14-F01	CG3988	gamma-soluble NSF attachment protein	416	936	111	305	**73.32**
plate1-F05	CG1152	Glucose dehydrogenase	431	1285	115	316	**73.32**
plate1-G01	CG1208	CG1208	450	1169	120	330	**73.33**
plate17-H09	CG4565	CG4565	30	128	8	22	**73.33**
plate19-F08	CG4836	CG4836	379	1027	101	278	**73.35**
plate13-F06	CG3810	Edem1	473	1220	126	347	**73.36**
plate4-E01	CG1776	CG42347	443	1187	118	325	**73.36**
plate14-D07	CG3957	wing morphogenesis defect	751	1663	200	551	**73.37**
plate2-B02	CG1309	CG1309	308	677	82	226	**73.38**
plate7-B08	CG2380	Nuclear factor I	586	1300	156	430	**73.38**
plate9-B12	CG3024	torp4a	406	1014	108	298	**73.40**
plate14-G07	CG4019	CG4019	609	1394	162	447	**73.40**
plate2-E07	CG1407	CG1407	579	1274	154	425	**73.40**
plate7-B01	CG2316	CG2316	361	912	96	265	**73.41**
plate19-G05	CG4848	CG4848	158	432	42	116	**73.42**
plate6-B11	CG2108	-	162	341	43	119	**73.46**
plate10-E06	CG3260	Zinc finger protein RP-8	196	539	52	144	**73.47**
plate8-H11	CG2987	alpha-catenin related	886	1988	235	651	**73.48**
plate4-E09	CG1792	CG1792	411	1030	109	302	**73.48**
plate8-A03	CG32656	CG32656	577	1151	153	424	**73.48**
plate15-C06	CG4096	CG4096	283	688	75	208	**73.50**
plate6-F10	CG2198	Amalgam	200	438	53	147	**73.50**
plate13-B09	CG3733	Chromodomain-helicase-DNA-binding protein 1	434	1156	115	319	**73.50**
plate9-D02	CG3041	Origin recognition complex subunit 2	921	2205	244	677	**73.51**
plate4-B05	CG1708	costa	302	1020	80	222	**73.51**
plate20-A06	CG4897	Ribosomal protein L7	102	272	27	75	**73.53**
plate11-C10	CG3400	6-phosphofructo-2-kinase	276	864	73	203	**73.55**
plate9-A12	CG3008	CG3008	242	545	64	178	**73.55**
plate19-A12	CG4743	CG4743	121	317	32	89	**73.55**
plate9-C02	CG3026	mus81	692	1541	183	509	**73.55**

plate8-D05	CG2893	CG2893	817	1734	216	601	**73.56**
plate7-D08	CG2540	CG2540	443	1052	117	326	**73.59**
plate2-D06	CG1379	CG34145	288	787	76	212	**73.61**
plate15-F10	CG4165	CG4165	398	997	105	293	**73.62**
plate19-G10	CG4858	CG4858	853	1969	225	628	**73.62**
plate12-G11	CG3680	CG3680	311	702	82	229	**73.63**
plate12-H10	CG3700	CG3700	311	780	82	229	**73.63**
plate14-A12	CG3902	CG3902	239	703	63	176	**73.64**
plate1-G05	CG1216	mrityu	277	600	73	204	**73.65**
plate13-E06	CG3792	CG3792	740	1880	195	545	**73.65**
plate14-A03	CG33052	CG33052	167	547	44	123	**73.65**
plate7-D06	CG2533	CG2533	186	403	49	137	**73.66**
plate8-E01	CG31548	CG31548	949	2138	250	699	**73.66**
plate7-A10	CG2302	nicotinic Acetylcholine Receptor alpha 7E	224	496	59	165	**73.66**
plate2-H01	CG1462	Alkaline phosphatase 4	691	1637	182	509	**73.66**
plate6-D04	CG2174	unconventional myosin class XV	524	1327	138	386	**73.66**
plate3-H07	CG1666	Helicase	285	674	75	210	**73.68**
plate4-A04	CG1688	CG1688	57	175	15	42	**73.68**
plate14-A04	CG3885	sec3	95	377	25	70	**73.68**
plate2-E11	CG1412	RhoGAP19D	449	1116	118	331	**73.72**
plate9-H05	CG32758	CG32758	411	924	108	303	**73.72**
plate12-B08	CG3561	KH1	609	1466	160	449	**73.73**
plate2-G06	CG1447	Ptx1	453	1091	119	334	**73.73**
plate5-A09	CG1887	CG1887	80	216	21	59	**73.75**
plate13-C08	CG3752	Aldehyde dehydrogenase	602	1570	158	444	**73.75**
plate12-C11	CG3603	CG3603	282	703	74	208	**73.76**
plate2-D10	CG1386	antdh	881	2179	231	650	**73.78**
plate11-B04	CG3359	midline fasciclin	820	1819	215	605	**73.78**
plate4-C11	CG1751	Spase 25-subunit	370	1117	97	273	**73.78**
plate2-A04	CG1275	CG1275	420	913	110	310	**73.81**
plate20-B08	CG4917	wolfram syndrome 1	317	955	83	234	**73.82**
plate20-E11	CG4972	CG4972	592	1671	155	437	**73.82**
plate4-D09	CG1771	multiple edematous wings	466	1218	122	344	**73.82**

plate1-E08	CG1131	alpha-Esterase-10	531	1296	139	392	**73.82**
plate13-C10	CG3756	CG3756	619	1610	162	457	**73.83**
plate19-D09	CG4799	Pendulin	470	1303	123	347	**73.83**
plate14-B11	CG3926	Serine pyruvate aminotransferase	214	525	56	158	**73.83**
plate2-H05	CG1470	Guanylyl cyclase beta-subunit at 100B	497	1142	130	367	**73.84**
plate19-E12	CG4824	Bicaudal C	88	276	23	65	**73.86**
plate5-B09	CG1907	CG1907	111	289	29	82	**73.87**
plate15-D06	CG4117	Magi	513	1260	134	379	**73.88**
plate19-B09	CG4757	CG4757	276	687	72	204	**73.91**
plate20-E02	CG4957	CG4957	368	994	96	272	**73.91**
plate8-H04	CG2979	Yolk protein 2	211	523	55	156	**73.93**
plate14-F09	CG4001	Phosphofructokinase	618	1398	161	457	**73.95**
plate12-C01	CG3573	CG3573	311	803	81	230	**73.95**
plate9-E06	CG3068	aurora	580	1401	151	429	**73.97**
plate3-E05	CG1598	CG1598	484	1161	126	358	**73.97**
plate5-D12	CG1966	ATP-dependent chromatin assembly factor large subunit	388	1079	101	287	**73.97**
plate15-C04	CG4094	lethal (1) G0255	73	285	19	54	**73.97**
plate6-B01	CG2096	flap wing	346	867	90	256	**73.99**
plate2-F10	CG1430	by S6	546	1399	142	404	**73.99**
plate2-A05	CG1276	Transcription factor IIEbeta	200	509	52	148	**74.00**
plate5-G11	CG2053	CG2053	250	728	65	185	**74.00**
plate15-H07	CG4203	CG4203	50	137	13	37	**74.00**
plate13-C03	CG3743	Metal response element-binding Transcription Factor-1	231	552	60	171	**74.03**
plate17-B10	CG4433	CG4433	77	181	20	57	**74.03**
plate9-E07	CG3069	TBP-associated factor 10b	181	412	47	134	**74.03**
plate19-A02	CG4723	CG4723	104	368	27	77	**74.04**
plate10-G05	CG3294	CG3294	131	322	34	97	**74.05**
plate8-G12	CG2974	CG2974	752	1773	195	557	**74.07**
plate8-C10	CG2875	CG2875	270	793	70	200	**74.07**
plate13-D02	CG3763	Fat body protein 2	351	823	91	260	**74.07**
plate9-G08	CG3125	lethal (1) G0060	328	762	85	243	**74.09**
plate3-D06	CG1567	C901	398	1405	103	295	**74.12**
plate12-G05	CG3666	Transferrin 3	317	730	82	235	**74.13**

plate2-A06	CG32311	zormin	611	1538	158	453	**74.14**
plate11-G09	CG3488	CG3488	515	1113	133	382	**74.17**
plate7-G11	CG2702	PSEA-binding protein 95kD	670	1532	173	497	**74.18**
plate10-G07	CG3297	minidiscs	732	1684	189	543	**74.18**
plate8-F03	CG2929	Pi4KIIalpha	705	1437	182	523	**74.18**
plate6-B07	CG2103	polypeptide GalNAc transferase 6	62	188	16	46	**74.19**
plate15-H11	CG4208	XRCC1	31	96	8	23	**74.19**
plate12-A06	CG3526	CG3526	655	1455	169	486	**74.20**
plate15-C02	CG4088	latheo	283	747	73	210	**74.20**
plate18-D11	CG33158	CG33158	194	511	50	144	**74.23**
plate6-F03	CG2184	Myosin light chain 2	520	1209	134	386	**74.23**
plate7-H10	CG2759	white	229	557	59	170	**74.24**
plate20-G11	CG5014	Vap-33-1	625	1708	161	464	**74.24**
plate2-H06	CG1471	-	365	912	94	271	**74.25**
plate1-G07	CG1225	-	167	362	43	124	**74.25**
plate11-F08	CG3466	Cytochrome P450-4d2	571	1233	147	424	**74.26**
plate3-B04	CG1514	CG1514	202	603	52	150	**74.26**
plate19-E02	CG4806	CG4806	136	345	35	101	**74.26**
plate9-D09	CG3054	lethal (2) k05819	482	1063	124	358	**74.27**
plate1-B07	CG1064	Snf5-related 1	105	329	27	78	**74.29**
plate12-C12	CG3604	CG3604	175	465	45	130	**74.29**
plate2-F06	CG1424	misato	740	1844	190	550	**74.32**
plate6-D02	CG31619	CG31619	468	1132	120	348	**74.36**
plate19-F11	CG4840	centrosomin's beautiful sister	823	2170	211	612	**74.36**
plate16-F06	CG4335	CG4335	316	789	81	235	**74.37**
plate10-E05	CG3259	CG3259	324	878	83	241	**74.38**
plate5-C11	CG1942	CG1942	203	758	52	151	**74.38**
plate11-G07	CG3485	-	621	1414	159	462	**74.40**
plate1-C05	CG1082	alpha-Esterase-4	215	814	55	160	**74.42**
plate6-E07	CG2163	Pabp2	43	209	11	32	**74.42**
plate10-H01	CG3309	CG3309	1177	3154	301	876	**74.43**
plate9-D03	CG32732	CG32732	962	2036	246	716	**74.43**
plate1-G06	CG1218	CG1218	219	741	56	163	**74.43**

plate1-F07	CG1165	Lysozyme S	403	899	103	300	**74.44**
plate4-G02	CG1824	CG1824	90	220	23	67	**74.44**
plate13-A11	CG3715	SHC-adaptor protein	137	392	35	102	**74.45**
plate14-G08	CG4020	CG4020	423	1007	108	315	**74.47**
plate16-B01	CG4238	CG4238	47	149	12	35	**74.47**
plate4-F01	CG1796	Transport and Golgi organization 4	98	257	25	73	**74.49**
plate11-B05	CG3360	Cyp313a1	490	1242	125	365	**74.49**
plate20-F12	CG4995	CG4995	294	830	75	219	**74.49**
plate19-A08	CG4733	CG4733	522	1359	133	389	**74.52**
plate15-C10	CG4103	CG4103	577	1307	147	430	**74.52**
plate8-G03	CG2950	CG2950	648	1497	165	483	**74.54**
plate19-B06	CG4753	CG4753	271	858	69	202	**74.54**
plate13-B08	CG3732	CG3732	735	1643	187	548	**74.56**
plate7-E01	CG32659	Tenascin accessory	346	821	88	258	**74.57**
plate6-B04	CG2100	CG2100	59	143	15	44	**74.58**
plate1-B09	CG1066	Shaker cognate b	59	227	15	44	**74.58**
plate4-H09	CG32677	X11Lbeta	362	841	92	270	**74.59**
plate9-G12	CG32758	CG32758	315	666	80	235	**74.60**
plate17-F10	CG4532	pod1	63	167	16	47	**74.60**
plate13-B02	CG3723	Dynein heavy chain at 93AB	457	1023	116	341	**74.62**
plate4-A12	CG1698	CG1698	276	673	70	206	**74.64**
plate4-H12	CG1869	Cht7	138	359	35	103	**74.64**
plate7-H02	CG2713	tiny tim 50	276	661	70	206	**74.64**
plate10-F11	CG3284	RNA polymerase II 15kD subunit	513	1290	130	383	**74.66**
plate8-B10	CG2848	Transportin-Serine/Arginine rich	150	449	38	112	**74.67**
plate8-H10	CG2985	Yolk protein 1	525	1164	133	392	**74.67**
plate15-E07	CG4143	multiprotein bridging factor 1	229	587	58	171	**74.67**
plate9-C05	CG3032	CG3032	324	732	82	242	**74.69**
plate19-F09	CG30103	CG30103	419	1005	106	313	**74.70**
plate19-G12	CG4860	CG4860	419	1107	106	313	**74.70**
plate6-B06	CG2102	castor	87	246	22	65	**74.71**
plate8-A01	CG2772	CG2772	491	1209	124	367	**74.75**
plate8-G07	CG2964	CG2964	519	1313	131	388	**74.76**

plate11-G04	CG3481	Alcohol dehydrogenase	642	1567	162	480	**74.77**
plate17-B08	CG4429	RNA-binding protein 2	107	278	27	80	**74.77**
plate20-A10	CG31151	winged eye	111	316	28	83	**74.77**
plate2-F11	CG1433	Another transcription unit	341	802	86	255	**74.78**
plate6-A10	CG2092	scraps	500	1112	126	374	**74.80**
plate18-A05	CG4578	cap-n-collar	127	355	32	95	**74.80**
plate8-D01	CG2887	CG2887	731	1624	184	547	**74.83**
plate13-F01	CG3800	CG3800	155	346	39	116	**74.84**
plate13-B11	CG3735	CG3735	338	841	85	253	**74.85**
plate8-B06	CG2835	G protein salpha 60A	354	888	89	265	**74.86**
plate1-D04	CG1102	Melanization Protease 1	947	2233	238	709	**74.87**
plate8-C09	CG2867	Phosphoribosylamidotransferase	386	810	97	289	**74.87**
plate13-B01	CG3722	shotgun	207	559	52	155	**74.88**
plate5-F05	CG2005	Protein tyrosine phosphatase 99A	215	629	54	161	**74.88**
plate15-G07	CG4180	lethal (2) 35Bg	466	1279	117	349	**74.89**
plate14-C06	CG3938	Cyclin E	235	697	59	176	**74.89**
plate6-G04	CG2218	CG2218	243	578	61	182	**74.90**
plate17-C05	CG4446	-	255	727	64	191	**74.90**
plate4-E02	CG1780	Imaginal disc growth factor 4	255	703	64	191	**74.90**
plate15-F06	CG4161	CG4161	267	658	67	200	**74.91**
plate14-B09	CG3924	Chip	279	635	70	209	**74.91**
plate4-G09	CG1837	CG1837	287	742	72	215	**74.91**
plate18-E06	CG4649	Sorbitol dehydrogenase-2	355	875	89	266	**74.93**
plate13-E05	CG32809	CG32809	563	1135	141	422	**74.96**
plate1-C10	CG1090	CG1090	304	862	76	228	**75.00**
plate4-H06	CG1851	Ady43A	40	97	10	30	**75.00**
plate5-E01	CG1967	p24-related-1	148	404	37	111	**75.00**
plate6-D06	CG2139	aralar1	332	826	83	249	**75.00**
plate6-H07	CG2257	Ubc-E2H	44	145	11	33	**75.00**
plate11-A07	CG3338	CG3338	72	214	18	54	**75.00**
plate11-C12	CG3403	-	20	172	5	15	**75.00**
plate11-H01	CG3495	GDP-4-keto-6-deoxy-D-mannose 3,5-epimerase/4-reductase	24	84	6	18	**75.00**
plate13-A12	CG3719	CG3719	24	104	6	18	**75.00**

plate15-A07	CG4059	ftz transcription factor 1	36	113	9	27	**75.00**
plate15-H08	CG4204	Elongin B	136	289	34	102	**75.00**
plate16-D12	CG4307	Oligomycin sensitivity-conferring protein	24	54	6	18	**75.00**
plate17-F06	CG4525	CG4525	116	327	29	87	**75.00**
plate19-E09	CG4821	Tequila	112	312	28	84	**75.00**
plate20-E08	CG4968	CG4968	436	1304	109	327	**75.00**
plate9-F03	CG3093	deep orange	673	1766	168	505	**75.04**
plate13-E08	CG3795	CG3795	565	1263	141	424	**75.04**
plate6-H08	CG2258	CG2258	341	864	85	256	**75.07**
plate2-C05	CG1343	Sp1	337	843	84	253	**75.07**
plate10-G01	CG3289	Phosphotyrosyl phosphatase activator	1581	3606	394	1187	**75.08**
plate18-G09	CG4696	Muscle protein 20	309	853	77	232	**75.08**
plate12-B10	CG3567	Cyp6u1	305	706	76	229	**75.08**
plate13-E02	CG32809	CG32809	289	628	72	217	**75.09**
plate12-F08	CG3653	kin of irre	1040	2667	259	781	**75.10**
plate11-E07	CG3436	CG3436	253	613	63	190	**75.10**
plate11-F01	CG3454	Histidine decarboxylase	418	1044	104	314	**75.12**
plate2-D11	CG1389	torso	615	1517	153	462	**75.12**
plate13-D06	CG3770	CG3770	398	960	99	299	**75.13**
plate6-D12	CG2152	Protein-L-isoaspartate (D-aspartate) O-methyltransferase	370	802	92	278	**75.14**
plate13-D07	CG3772	cryptochrome	435	1114	108	327	**75.17**
plate12-D08	CG3619	Delta	540	1286	134	406	**75.19**
plate2-A02	CG1271	CG1271	250	660	62	188	**75.20**
plate11-D04	CG3411	blistered	738	1843	183	555	**75.20**
plate14-B04	CG3915	Derailed 2	973	2129	241	732	**75.23**
plate3-F05	CG1620	CG1620	424	1338	105	319	**75.24**
plate14-B05	CG3916	CG3916	735	1883	182	553	**75.24**
plate20-E10	CG4971	Wnt10	828	2225	205	623	**75.24**
plate9-G11	CG3129	Rab-related protein 4	1127	2759	279	848	**75.24**
plate19-B10	CG4758	Translocation protein 1	303	791	75	228	**75.25**
plate1-A02	CG1009	Puromycin sensitive aminopeptidase	400	1091	99	301	**75.25**
plate12-B04	CG3544	CG3544	291	761	72	219	**75.26**
plate14-H07	CG4039	Minichromosome maintenance 6	97	339	24	73	**75.26**

plate9-D04	CG3044	Cht11	946	2435	234	712	**75.26**
plate7-E07	CG2616	CG2616	275	643	68	207	**75.27**
plate7-H04	CG2718	Glutamine synthetase 1	1007	2285	249	758	**75.27**
plate9-A07	CG2999	unc-13	178	410	44	134	**75.28**
plate10-F10	CG3283	Succinate dehydrogenase B	672	1514	166	506	**75.30**
plate19-A11	CG4742	mitochondrial ribosomal protein L22	138	426	34	104	**75.36**
plate11-C01	CG3382	Organic anion transporting polypeptide 58Db	203	566	50	153	**75.37**
plate2-E12	CG1414	bobby sox	390	927	96	294	**75.38**
plate3-F08	CG32648	Phosphodiesterase 9	130	364	32	98	**75.38**
plate15-D10	CG4122	silver	585	1435	144	441	**75.38**
plate10-C08	CG3213	CG3213	317	896	78	239	**75.39**
plate8-D10	CG2904	echinus	313	869	77	236	**75.40**
plate10-G11	CG3307	pr-set7	362	1106	89	273	**75.41**
plate8-A09	CG2794	CG2794	301	687	74	227	**75.42**
plate2-D09	CG30497	CG30497	240	787	59	181	**75.42**
plate9-D01	CG3040	CG3040	582	1425	143	439	**75.43**
plate19-B04	CG4751	CG4751	696	1844	171	525	**75.43**
plate3-C10	CG1544	CG1544	285	715	70	215	**75.44**
plate3-E06	CG1599	CG1599	171	747	42	129	**75.44**
plate13-F05	CG3809	CG3809	440	1029	108	332	**75.45**
plate11-E03	CG3428	pallbearer	493	1398	121	372	**75.46**
plate4-D10	CG1773	CG1773	314	763	77	237	**75.48**
plate9-G06	CG3121	CG3121	208	451	51	157	**75.48**
plate12-E07	CG3635	CG3635	779	2188	191	588	**75.48**
plate12-D03	CG3608	CG3608	837	1925	205	632	**75.51**
plate18-C06	CG4612	CG4612	196	528	48	148	**75.51**
plate8-B08	CG2845	pole hole	237	604	58	179	**75.53**
plate19-F10	CG4839	CG4839	797	2095	195	602	**75.53**
plate8-G01	CG2947	CG2947	973	2042	238	735	**75.54**
plate12-E03	CG3631	CG3631	900	2184	220	680	**75.56**
plate15-H05	CG4200	small wing	90	384	22	68	**75.56**
plate13-G10	CG3849	Lasp	311	843	76	235	**75.56**
plate5-G06	CG2047	fushi tarazu	176	525	43	133	**75.57**

plate20-F05	CG4979	sex-specific enzyme 2	508	1423	124	384	**75.59**
plate12-E10	CG3639	CG3639	668	1860	163	505	**75.60**
plate2-H12	CG1486	CG1486	250	568	61	189	**75.60**
plate2-E01	CG1399	CG1399	582	1400	142	440	**75.60**
plate20-F10	CG4991	CG4991	742	2016	181	561	**75.61**
plate8-F02	CG2926	CG2926	238	604	58	180	**75.63**
plate2-C11	CG1358	CG1358	197	556	48	149	**75.63**
plate10-G10	CG3304	-	472	1158	115	357	**75.64**
plate17-C09	CG4451	Heparan sulfate 6-O-sulfotransferase	78	238	19	59	**75.64**
plate4-H04	CG1847	CG1847	74	217	18	56	**75.68**
plate12-H08	CG3697	meiotic 9	444	1135	108	336	**75.68**
plate5-D10	CG1964	Kuzbanian-like	177	537	43	134	**75.71**
plate7-G07	CG2692	gooseberry-neuro	671	1452	163	508	**75.71**
plate1-H04	CG1244	CG1244	704	2094	171	533	**75.71**
plate15-G09	CG4183	Heat shock protein 26	140	444	34	106	**75.71**
plate19-F03	CG4827	CG4827	173	459	42	131	**75.72**
plate15-D03	CG4110	pickpocket 11	412	973	100	312	**75.73**
plate18-A12	CG4587	CG4587	103	188	25	78	**75.73**
plate6-G03	CG2212	swiss cheese	375	920	91	284	**75.73**
plate8-G08	CG2967	-	746	1745	181	565	**75.74**
plate11-E05	CG3431	Ubiquitin C-terminal hydrolase	264	739	64	200	**75.76**
plate17-D11	CG4481	Glutamate receptor IB	330	774	80	250	**75.76**
plate18-G02	CG4678	CG4678	161	493	39	122	**75.78**
plate3-C05	CG1532	CG1532	413	1210	100	313	**75.79**
plate10-A09	CG3168	CG3168	413	1032	100	313	**75.79**
plate10-H05	CG3315	Thioredoxin T	95	240	23	72	**75.79**
plate19-H04	CG4867	bc10	252	791	61	191	**75.79**
plate1-E05	CG1128	alpha-Esterase-9	438	1181	106	332	**75.80**
plate12-A05	CG3525	easily shocked	186	484	45	141	**75.81**
plate3-A12	CG1506	Ac3	215	608	52	163	**75.81**
plate10-C05	CG3209	CG3209	335	898	81	254	**75.82**
plate5-E10	CG1982	Sorbitol dehydrogenase 1	273	728	66	207	**75.82**
plate7-A06	CG30375	CG30375	91	230	22	69	**75.82**

plate13-G09	CG3848	trithorax-related	182	583	44	138	**75.82**
plate1-D05	CG1104	CG1104	120	466	29	91	**75.83**
plate13-F08	CG3812	CG3812	120	291	29	91	**75.83**
plate12-G12	CG3682	PIP5K59B	149	402	36	113	**75.84**
plate4-D01	CG1756	CG1756	207	573	50	157	**75.85**
plate18-E10	CG4656	Ras association family member	381	957	92	289	**75.85**
plate6-G07	CG2227	GIP-like	87	226	21	66	**75.86**
plate11-H10	CG3508	CG3508	145	637	35	110	**75.86**
plate14-E12	CG3987	CG3987	58	150	14	44	**75.86**
plate16-C08	CG4272	CG4272	116	310	28	88	**75.86**
plate13-C09	CG3753	Marcal1	692	1913	167	525	**75.87**
plate19-H03	CG4866	CG4866	1157	2487	279	878	**75.89**
plate4-B10	CG1728	Tim8	394	1010	95	299	**75.89**
plate1-F02	CG1141	CG33232	776	2047	187	589	**75.90**
plate15-H10	CG4206	Minichromosome maintenance 3	291	899	70	221	**75.95**
plate10-H06	CG3319	Cyclin-dependent kinase 7	524	1254	126	398	**75.95**
plate19-D11	CG4802	CG4802	732	2008	176	556	**75.96**
plate10-E12	CG3268	phtf	936	2225	225	711	**75.96**
plate20-D10	CG4952	dachshund	828	1961	199	629	**75.97**
plate18-B07	CG4594	CG4594	129	339	31	98	**75.97**
plate6-C02	CG2112	CG42233	325	865	78	247	**76.00**
plate9-C03	CG3027	pyd3	575	1471	138	437	**76.00**
plate13-H09	CG30421	CG30421	25	68	6	19	**76.00**
plate11-H09	CG3506	vasa	50	180	12	38	**76.00**
plate13-A01	CG3703	CG3703	25	68	6	19	**76.00**
plate19-D04	CG4793	CG4793	321	887	77	244	**76.01**
plate9-H02	CG3132	Ect3	317	760	76	241	**76.03**
plate13-G04	CG3837	CG3837	580	1346	139	441	**76.03**
plate12-F05	CG3648	CG32751	476	1160	114	362	**76.05**
plate4-F10	CG1815	CG1815	309	717	74	235	**76.05**
plate8-D04	CG32687	CG32687	522	1124	125	397	**76.05**
plate2-F02	CG1417	sluggish A	426	1194	102	324	**76.06**
plate10-E10	CG3265	Eb1	781	1950	187	594	**76.06**

plate1-E06	CG1129	CG1129	117	377	28	89	**76.07**
plate17-F02	CG4511	CG4511	468	1198	112	356	**76.07**
plate18-F08	CG4672	TMS1	255	676	61	194	**76.08**
plate7-A05	CG2277	CG2277	636	1480	152	484	**76.10**
plate5-F07	CG31536	Cdep	159	505	38	121	**76.10**
plate18-B10	CG4600	yippee interacting protein 2	159	524	38	121	**76.10**
plate6-H04	CG2253	Upf2	113	252	27	86	**76.11**
plate8-A04	CG2781	CG2781	578	1274	138	440	**76.12**
plate12-C08	CG3595	spaghetti squash	687	1810	164	523	**76.13**
plate3-C08	CG1542	CG1542	109	373	26	83	**76.15**
plate16-F12	CG4349	Ferritin 3 heavy chain homologue	109	297	26	83	**76.15**
plate4-F02	CG1799	raspberry	172	530	41	131	**76.16**
plate19-A05	CG4726	CG4726	172	490	41	131	**76.16**
plate4-F04	CG1801	CG1801	214	579	51	163	**76.17**
plate1-F01	CG1140	CG1140	701	1586	167	534	**76.18**
plate5-E02	CG1968	CG1968	269	985	64	205	**76.21**
plate6-F02	CG2183	CG2183	366	831	87	279	**76.23**
plate12-F06	CG3649	CG3649	644	1530	153	491	**76.24**
plate4-B06	CG1710	Host cell factor	181	568	43	138	**76.24**
plate10-C06	CG3210	Dynamin related protein 1	299	757	71	228	**76.25**
plate10-E03	CG3252	CG3252	396	1031	94	302	**76.26**
plate11-G05	CG3483	CG3483	653	1579	155	498	**76.26**
plate10-D11	CG3245	Protein phosphatase N at 58A	493	1148	117	376	**76.27**
plate7-E02	CG32659	Tenascin accessory	249	600	59	190	**76.31**
plate14-A09	CG3895	polyhomeotic distal	114	297	27	87	**76.32**
plate16-H03	CG4376	alpha actinin	38	160	9	29	**76.32**
plate2-A08	CG1291	CG1291	262	682	62	200	**76.34**
plate2-E02	CG1401	cullin-5	486	1463	115	371	**76.34**
plate6-G01	CG2204	G protein oalpha 47A	224	648	53	171	**76.34**
plate8-E08	CG2918	CG2918	592	1555	140	452	**76.35**
plate11-D07	CG3416	Mov34	55	163	13	42	**76.36**
plate18-D10	CG4637	hedgehog	330	857	78	252	**76.36**
plate1-C04	CG1081	Rheb	326	1007	77	249	**76.38**

plate11-H06	CG3502	CG3502	72	224	17	55	**76.39**
plate17-D07	CG4472	Imaginal disc growth factor 1	305	768	72	233	**76.39**
plate12-C10	CG3599	Biotinidase	644	1674	152	492	**76.40**
plate17-A03	CG4395	CG4395	161	490	38	123	**76.40**
plate6-A02	CG2078	Myd88	356	1051	84	272	**76.40**
plate6-F08	CG2194	suppressor of rudimentary	373	817	88	285	**76.41**
plate5-D11	CG1965	CG1965	246	908	58	188	**76.42**
plate12-H07	CG3696	kismet	225	606	53	172	**76.44**
plate1-B01	CG1056	Serotonin receptor 2	760	2002	179	581	**76.45**
plate9-C06	CG3033	CG3033	327	779	77	250	**76.45**
plate1-A01	CG1004	rhomboid	633	1740	149	484	**76.46**
plate7-E09	CG2637	Female sterile (2) Ketel	85	226	20	65	**76.47**
plate16-G01	CG32707	Anaphase Promoting Complex 4	51	129	12	39	**76.47**
plate20-B10	CG4919	Glutamate-cysteine ligase modifier subunit	323	901	76	247	**76.47**
plate10-A08	CG3167	MAN1	302	877	71	231	**76.49**
plate5-F06	CG2006	CG2006	200	522	47	153	**76.50**
plate17-C07	CG4448	will decrease acetylation	200	471	47	153	**76.50**
plate8-B01	CG2818	CG2818	166	365	39	127	**76.51**
plate9-F04	CG3097	CG3097	643	1475	151	492	**76.52**
plate3-A06	CG1494	CG1494	115	411	27	88	**76.52**
plate12-F10	CG3655	CG3655	196	523	46	150	**76.53**
plate19-B08	CG4756	CG4756	277	747	65	212	**76.53**
plate3-B08	CG1518	CG1518	81	225	19	62	**76.54**
plate7-F04	CG31187	-	324	711	76	248	**76.54**
plate7-C08	CG2503	antimeros	273	697	64	209	**76.56**
plate7-A03	CG2272	slipper	128	304	30	98	**76.56**
plate13-G02	CG3829	CG3829	192	526	45	147	**76.56**
plate13-C05	CG3747	Excitatory amino acid transporter 1	709	1865	166	543	**76.59**
plate11-C09	CG3399	cappuccino	299	807	70	229	**76.59**
plate2-B10	CG1322	Zn finger homeodomain 1	141	465	33	108	**76.60**
plate14-C05	CG3937	cheerio	705	1705	165	540	**76.60**
plate9-E02	CG3060	morula	218	528	51	167	**76.61**
plate17-F07	CG32158	-	321	809	75	246	**76.64**

plate12-C07	CG3593	rudimentary-like	668	1731	156	512	**76.65**
plate18-D05	CG4629	CG4629	424	1252	99	325	**76.65**
plate16-C10	CG4274	fizzy	90	307	21	69	**76.67**
plate10-C01	CG3200	Rhythmically expressed gene 2	502	1301	117	385	**76.69**
plate19-D07	CG4797	CG4797	369	920	86	283	**76.69**
plate7-G09	CG2699	Pi3K21B	309	744	72	237	**76.70**
plate11-B10	CG3376	CG3376	176	545	41	135	**76.70**
plate8-H02	CG2976	CG2976	563	1487	131	432	**76.73**
plate2-D12	CG1395	string	301	814	70	231	**76.74**
plate11-B11	CG3379	Histone H4 replacement	129	361	30	99	**76.74**
plate11-F03	CG3456	Monocarboxylate transporter 1	598	1517	139	459	**76.76**
plate20-E09	CG4969	Wnt6	908	2611	211	697	**76.76**
plate3-G08	CG30502	CG30502	340	942	79	261	**76.76**
plate7-G03	CG2682	d4	551	1274	128	423	**76.77**
plate11-B06	CG3362	CG3362	280	771	65	215	**76.79**
plate7-C09	CG2505	alpha-Esterase-2	405	952	94	311	**76.79**
plate7-F02	CG2656	CG2656	418	907	97	321	**76.79**
plate4-G04	CG1827	CG1827	69	224	16	53	**76.81**
plate8-D12	CG2906	CG2906	276	964	64	212	**76.81**
plate10-E04	CG3254	polypeptide GalNAc transferase 2	69	199	16	53	**76.81**
plate11-C05	CG3389	Cad88C	565	1435	131	434	**76.81**
plate14-F02	CG3989	ade5	673	1751	156	517	**76.82**
plate8-D08	CG2902	NMDA receptor 1	233	646	54	179	**76.82**
plate13-C06	CG30420	Activating transcription factor-2	233	598	54	179	**76.82**
plate13-A04	CG3706	CG3706	82	248	19	63	**76.83**
plate18-F09	CG4673	CG4673	95	376	22	73	**76.84**
plate1-C09	CG1089	alpha-Esterase-5	203	569	47	156	**76.85**
plate15-F08	CG4163	Cyp303a1	216	535	50	166	**76.85**
plate9-E01	CG3059	NTPase	998	2432	231	767	**76.85**
plate11-E04	CG3430	CG3430	458	1293	106	352	**76.86**
plate10-E07	CG3262	CG3262	731	1837	169	562	**76.88**
plate5-H02	CG2056	Serine Protease Immune Response Integrator	212	652	49	163	**76.89**
plate20-H04	CG5023	CG5023	450	1308	104	346	**76.89**

plate8-C07	CG2863	Notchless	342	888	79	263	**76.90**
plate3-E08	CG1602	CG1602	429	1274	99	330	**76.92**
plate3-G12	CG1651	Ankyrin	182	492	42	140	**76.92**
plate5-F08	CG2009	bip2	299	1002	69	230	**76.92**
plate14-G02	CG4009	CG4009	364	1028	84	280	**76.92**
plate16-E12	CG4328	CG4328	26	70	6	20	**76.92**
plate3-C03	CG1530	CG1530	529	1607	122	407	**76.94**
plate12-A07	CG3527	CG3527	386	971	89	297	**76.94**
plate8-B09	CG2846	CG2846	178	411	41	137	**76.97**
plate8-E02	CG2908	CG2908	508	1193	117	391	**76.97**
plate6-A04	CG2082	CG2082	165	455	38	127	**76.97**
plate13-D11	CG3780	Spliceosomal protein on the X	278	901	64	214	**76.98**
plate1-D08	CG1107	-	365	875	84	281	**76.99**
plate16-D05	CG4291	CG4291	100	281	23	77	**77.00**
plate16-G03	CG4353	hemipterous	370	993	85	285	**77.03**
plate17-B06	CG4424	CG4424	74	206	17	57	**77.03**
plate15-F05	CG4159	CG4159	653	1726	150	503	**77.03**
plate2-F08	CG1428	CG1428	122	340	28	94	**77.05**
plate19-E06	CG4816	quaking related 54B	750	2086	172	578	**77.07**
plate7-B02	CG2321	CG2321	240	568	55	185	**77.08**
plate17-C11	CG4453	Nup153	96	378	22	74	**77.08**
plate14-D05	CG3954	corkscrew	729	1947	167	562	**77.09**
plate14-F03	CG3991	tripeptidyl-peptidase II	585	1606	134	451	**77.09**
plate18-G07	CG4686	CG4686	262	687	60	202	**77.10**
plate12-A02	CG3522	Start1	332	938	76	256	**77.11**
plate20-B07	CG4916	maternal expression at 31B	284	881	65	219	**77.11**
plate13-F11	CG3821	Aspartyl-tRNA synthetase	319	875	73	246	**77.12**
plate19-C04	CG4767	Tektin A	796	2261	182	614	**77.14**
plate15-E10	CG4148	weckle	521	1413	119	402	**77.16**
plate12-D02	CG3606	cabeza	552	1459	126	426	**77.17**
plate7-F01	CG2655	Helix loop helix protein 3B	592	1303	135	457	**77.20**
plate4-H03	CG1846	CG42271	443	1050	101	342	**77.20**
plate11-F05	CG30011	gemini	329	918	75	254	**77.20**

plate17-G07	CG4548	XNP	101	252	23	78	**77.23**
plate14-F08	CG3999	CG3999	593	1530	135	458	**77.23**
plate3-B06	CG1516	CG1516	145	512	33	112	**77.24**
plate19-E08	CG4820	CG4820	778	2102	177	601	**77.25**
plate20-F01	CG4974	division abnormally delayed	211	657	48	163	**77.25**
plate7-C07	CG2493	CG2493	444	1019	101	343	**77.25**
plate12-B02	CG3541	piopio	444	1065	101	343	**77.25**
plate1-C01	CG1074	CG1074	343	982	78	265	**77.26**
plate13-D04	CG31475	CG31475	585	1529	133	452	**77.26**
plate19-E11	CG31092	LpR2	739	2155	168	571	**77.27**
plate7-F08	CG2674	Minute (2) 21AB	176	550	40	136	**77.27**
plate13-G03	CG3835	CG3835	220	454	50	170	**77.27**
plate14-H11	CG4043	Rrp46	44	150	10	34	**77.27**
plate17-A09	CG4407	CG4407	22	48	5	17	**77.27**
plate12-A09	CG3530	CG3530	207	579	47	160	**77.29**
plate20-H08	CG5027	CG5027	551	1691	125	426	**77.31**
plate16-F05	CG4334	CG4334	344	984	78	266	**77.33**
plate11-E02	CG3427	Epac	322	859	73	249	**77.33**
plate10-D07	CG3234	timeless	574	1325	130	444	**77.35**
plate19-G01	CG4842	CG4842	159	449	36	123	**77.36**
plate9-B06	CG3017	Aminolevulinate synthase	349	957	79	270	**77.36**
plate4-B01	CG1702	CG1702	345	1084	78	267	**77.39**
plate5-D03	CG1950	CG1950	115	320	26	89	**77.39**
plate19-H10	CG4879	homolog of RecQ	553	1674	125	428	**77.40**
plate2-H09	CG1474	Es2	155	372	35	120	**77.42**
plate14-D12	CG3964	CG3964	31	109	7	24	**77.42**
plate18-E01	CG4643	CG4643	62	204	14	48	**77.42**
plate14-C08	CG3940	CG3940	474	1007	107	367	**77.43**
plate8-D09	CG2903	Hepatocyte growth factor regulated tyrosine kinase substrate	266	711	60	206	**77.44**
plate3-C12	CG1548	cathD	408	1251	92	316	**77.45**
plate12-F07	CG3650	CG3650	856	2197	193	663	**77.45**
plate12-A08	CG3529	CG3529	692	1734	156	536	**77.46**
plate20-F09	CG4989	CG4989	834	2346	188	646	**77.46**

plate2-G02	CG1440	CG1440	386	1056	87	299	**77.46**
plate14-B03	CG3911	CG3911	617	1512	139	478	**77.47**
plate17-F05	CG4523	PTEN-induced putative kinase 1	111	325	25	86	**77.48**
plate16-G06	CG4357	sodium chloride cotransporter 69	231	758	52	179	**77.49**
plate1-B02	CG1057	Mediator complex subunit 31	400	1346	90	310	**77.50**
plate4-G12	CG1841	Transport and Golgi organization	280	780	63	217	**77.50**
plate14-B12	CG3927	CG3927	40	142	9	31	**77.50**
plate9-G03	CG3114	erect wing	667	1566	150	517	**77.51**
plate3-A07	CG1497	CG32523	507	1237	114	393	**77.51**
plate6-F01	CG2182	CG2182	218	536	49	169	**77.52**
plate7-E04	CG2608	CG2608	307	794	69	238	**77.52**
plate10-A04	CG3161	deadpan	89	287	20	69	**77.53**
plate5-F09	CG2010	CG2010	343	828	77	266	**77.55**
plate19-D10	CG4800	Translationally controlled tumor protein	392	1048	88	304	**77.55**
plate17-B05	CG4422	GDP dissociation inhibitor	303	855	68	235	**77.56**
plate7-D03	CG2525	Hus1-like	156	423	35	121	**77.56**
plate16-E03	CG4316	Stubble	370	1021	83	287	**77.57**
plate12-G07	CG3669	CG3669	941	2016	211	730	**77.58**
plate7-E03	CG2604	CG2604	232	608	52	180	**77.59**
plate13-F02	CG3803	CG3803	366	1013	82	284	**77.60**
plate12-C04	CG3584	quaking related 58E-3	250	740	56	194	**77.60**
plate1-E10	CG1133	odd paired	268	716	60	208	**77.61**
plate8-C04	CG30427	CG30427	429	909	96	333	**77.62**
plate10-B01	CG3174	Flavin-containing monooxygenase 2	581	1524	130	451	**77.62**
plate19-H05	CG4869	beta-Tubulin at 97EF	626	1539	140	486	**77.64**
plate11-A09	CG3344	CG3344	170	431	38	132	**77.65**
plate3-A05	CG1492	CG1492	358	1121	80	278	**77.65**
plate15-G11	CG4185	NC2beta	273	839	61	212	**77.66**
plate7-C02	CG2453	CG2453	197	491	44	153	**77.66**
plate12-G03	CG3664	Rab-protein 5	197	562	44	153	**77.66**
plate15-E03	CG4136	CG4136	197	547	44	153	**77.66**
plate9-E04	CG3065	CG3065	600	1331	134	466	**77.67**
plate10-H02	CG3312	RNA-binding protein 4F	1030	2489	230	800	**77.67**

plate12-C09	CG3597	CG3597	412	921	92	320	**77.67**
plate10-D12	CG3248	Cog3	314	919	70	244	**77.71**
plate12-D07	CG3616	Cytochrome P450-9c1	516	1464	115	401	**77.71**
plate15-B08	CG4080	CG4080	175	506	39	136	**77.71**
plate11-D02	CG3408	CG3408	543	1373	121	422	**77.72**
plate15-G03	CG4170	vasa intronic gene	184	529	41	143	**77.72**
plate11-E10	CG3445	pleiohomeotic like	422	1293	94	328	**77.73**
plate14-D08	CG3959	pelota	1073	2496	239	834	**77.73**
plate4-B11	CG15920	resilin	319	941	71	248	**77.74**
plate11-G02	CG3479	outspread	517	1492	115	402	**77.76**
plate1-A11	CG1048	zerknullt-related	81	317	18	63	**77.78**
plate5-E07	CG1976	RhoGAP100F	126	373	28	98	**77.78**
plate6-C09	CG31619	CG31619	63	144	14	49	**77.78**
plate14-D01	CG3947	CG3947	513	1205	114	399	**77.78**
plate14-G05	CG4016	Serine palmitoyltransferase subunit I	243	634	54	189	**77.78**
plate15-D02	CG4108	-	333	922	74	259	**77.78**
plate16-F01	CG4329	CG4329	54	156	12	42	**77.78**
plate17-H07	CG4562	CG4562	45	137	10	35	**77.78**
plate18-H11	CG4720	Protein kinase at 92B	36	116	8	28	**77.78**
plate5-F01	CG1989	Yippee	658	1717	146	512	**77.81**
plate12-G04	CG3665	Fasciclin 2	631	1528	140	491	**77.81**
plate5-D08	CG1962	CG1962	302	780	67	235	**77.81**
plate12-H02	CG3688	lethal (2) 35Bd	221	734	49	172	**77.83**
plate1-A09	CG1041	CG1041	140	410	31	109	**77.86**
plate3-C06	CG1536	singed	420	1454	93	327	**77.86**
plate12-D01	CG3605	CG3605	131	515	29	102	**77.86**
plate20-C04	CG4926	Ror	434	1299	96	338	**77.88**
plate20-G09	CG5010	CG5010	398	1193	88	310	**77.89**
plate5-A11	CG1890	CG1890	86	473	19	67	**77.91**
plate16-F10	CG4338	CG4338	385	1177	85	300	**77.92**
plate19-D03	CG4792	Dicer-1	308	873	68	240	**77.92**
plate4-C02	CG1740	Nuclear transport factor-2	213	690	47	166	**77.93**
plate16-G09	CG4370		272	842	60	212	**77.94**

plate18-B06	CG4593	CG4593	127	339	28	99	**77.95**
plate11-C07	CG3394	CG3394	490	1247	108	382	**77.96**
plate14-E08	CG3980	CG3980	590	1495	130	460	**77.97**
plate2-C06	CG1344	CG1344	336	948	74	262	**77.98**
plate1-A10	CG1046	zerknullt	418	1113	92	326	**77.99**
plate1-H08	CG1249	snRNP2	100	343	22	78	**78.00**
plate6-E06	CG2162	CG2162	350	737	77	273	**78.00**
plate11-A11	CG3352	fat	50	184	11	39	**78.00**
plate19-D02	CG4789	CG4789	350	969	77	273	**78.00**
plate8-G02	CG2948	rev7	323	834	71	252	**78.02**
plate20-D08	CG4948	Tequila	405	1073	89	316	**78.02**
plate20-H06	CG5025	Selenophosphate synthetase 2	132	441	29	103	**78.03**
plate15-E06	CG4141	Pi3K92E	419	1066	92	327	**78.04**
plate3-A09	CG1500	furrowed	205	622	45	160	**78.05**
plate4-B04	CG1705	Methoprene-tolerant	82	307	18	64	**78.05**
plate6-C10	CG2124	CG2124	328	871	72	256	**78.05**
plate6-G12	CG2245	lethal (3) s1921	205	500	45	160	**78.05**
plate19-F05	CG4829	CG4829	319	877	70	249	**78.06**
plate19-G02	CG4843	Tropomyosin 2	310	822	68	242	**78.06**
plate9-B01	CG3009	CG3009	424	1151	93	331	**78.07**
plate1-G02	CG1210	Protein kinase 61C	602	1652	132	470	**78.07**
plate19-F07	CG4831	CG33695	885	2472	194	691	**78.08**
plate6-D08	CG2144	CG2144	146	326	32	114	**78.08**
plate7-D09	CG2543	CG2543	575	1400	126	449	**78.09**
plate10-C09	CG3216	CG3216	137	360	30	107	**78.10**
plate1-C12	CG1093	pollux	306	875	67	239	**78.10**
plate9-F01	CG3088	CG3088	950	2213	208	742	**78.11**
plate4-C03	CG1742	Microsomal glutathione S-transferase-like	192	537	42	150	**78.13**
plate14-B07	CG3921	CG3921	192	525	42	150	**78.13**
plate10-D10	CG3242	sister of odd and bowl	389	1057	85	304	**78.15**
plate10-E01	CG3249	yu	1172	2684	256	916	**78.16**
plate2-C07	CG1345	Glutamine:fructose-6-phosphate aminotransferase 2	252	712	55	197	**78.17**
plate11-H05	CG3501	CG3501	55	239	12	43	**78.18**

plate9-B04	CG3014	CG3014	656	1880	143	513	**78.20**
plate9-B02	CG3011	CG3011	546	1302	119	427	**78.21**
plate15-D07	CG4118	nuclear RNA export factor 2	514	1383	112	402	**78.21**
plate1-A04	CG1019	Muscle LIM protein at 84B	202	671	44	158	**78.22**
plate16-D02	CG4288	CG4288	101	330	22	79	**78.22**
plate14-E07	CG3979	I'm not dead yet	225	505	49	176	**78.22**
plate19-H08	CG4875	CG4875	147	429	32	115	**78.23**
plate15-E08	CG4145	Collagen type IV	340	901	74	266	**78.24**
plate6-E03	CG32484	Sphingosine kinase 2	524	1359	114	410	**78.24**
plate19-G11	CG4859	Matrix metalloproteinase 1	708	2063	154	554	**78.25**
plate2-H04	CG1467	Syntaxin 16	446	1059	97	349	**78.25**
plate6-D10	CG2146	dilute class unconventional myosin	230	660	50	180	**78.26**
plate16-G07	CG4360	CG4360	161	508	35	126	**78.26**
plate10-C02	CG3201	Myosin light chain cytoplasmic	474	1425	103	371	**78.27**
plate3-C04	CG1531	CG1531	465	1416	101	364	**78.28**
plate18-C10	CG4620	unkempt	378	1109	82	296	**78.31**
plate19-D12	CG4803	Tak1-like 2	309	767	67	242	**78.32**
plate19-C05	CG4769	CG4769	346	1167	75	271	**78.32**
plate14-E03	CG3969	Fak-like tyrosine kinase	706	1890	153	553	**78.33**
plate5-B01	CG1893	scramblase 2	383	984	83	300	**78.33**
plate5-F04	CG2003	CG2003	420	1215	91	329	**78.33**
plate1-B05	CG32465	CG34127	314	966	68	246	**78.34**
plate7-C01	CG2448	alpha1,6-fucosyltransferase	254	685	55	199	**78.35**
plate1-D01	CG1098	MLF1-adaptor molecule	231	862	50	181	**78.35**
plate8-F01	CG2925	noisette	268	877	58	210	**78.36**
plate14-C03	CG3935	aristaless	647	1720	140	507	**78.36**
plate17-G02	CG4538	CG4538	430	1236	93	337	**78.37**
plate20-H01	CG5020	Cytoplasmic linker protein 190	111	316	24	87	**78.38**
plate2-C04	CG1342	CG1342	310	878	67	243	**78.39**
plate3-H08	CG1669	kappaB-Ras	213	568	46	167	**78.40**
plate19-G04	CG4847	CG4847	301	745	65	236	**78.41**
plate1-D06	CG1105	CG1105	176	528	38	138	**78.41**
plate13-D05	CG3769	CG3769	366	894	79	287	**78.42**

plate12-A04	CG3524	v(2)k05816	570	1514	123	447	**78.42**
plate13-B05	CG3727	dreadlocks	612	1518	132	480	**78.43**
plate18-G10	CG4697	COP9 complex homolog subunit 1 a	204	635	44	160	**78.43**
plate12-C06	CG3590	CG3590	654	1736	141	513	**78.44**
plate11-H04	CG3500	CG3500	116	693	25	91	**78.45**
plate12-E01	CG3629	Distal-less	543	1333	117	426	**78.45**
plate1-D02	CG1099	Dynamin associated protein 160	608	1910	131	477	**78.45**
plate6-A05	CG2083	CG2083	130	387	28	102	**78.46**
plate13-G08	CG3847	CG3847	130	399	28	102	**78.46**
plate5-H01	CG2049	Protein kinase related to protein kinase N	288	915	62	226	**78.47**
plate10-G12	CG3308	CG3308	144	392	31	113	**78.47**
plate5-A10	CG1889	CG1889	251	590	54	197	**78.49**
plate17-E03	CG4485	Cytochrome P450-9b1	559	1513	120	439	**78.53**
plate20-H09	CG5028	CG5028	373	1249	80	293	**78.55**
plate15-B01	CG4068	CG4068	70	259	15	55	**78.57**
plate1-E03	CG1121	alpha-Esterase-8	420	1195	90	330	**78.57**
plate8-G11	CG2972	CG2972	294	872	63	231	**78.57**
plate12-C05	CG3585	CG3585	280	783	60	220	**78.57**
plate16-E10	CG4323	CG4323	84	235	18	66	**78.57**
plate18-B04	CG4591	Tetraspanin 86D	126	378	27	99	**78.57**
plate18-F03	CG4663	CG4663	140	409	30	110	**78.57**
plate19-C02	CG4764	CG4764	98	288	21	77	**78.57**
plate20-C05	CG4927	CG4927	224	763	48	176	**78.57**
plate20-D11	CG4953	CG4953	574	1738	123	451	**78.57**
plate14-B02	CG3905	Suppressor of zeste 2	411	959	88	323	**78.59**
plate7-G04	CG2684	lodestar	374	916	80	294	**78.61**
plate2-B04	CG1315	CG1315	533	1379	114	419	**78.61**
plate11-H03	CG3499	CG3499	173	588	37	136	**78.61**
plate1-B03	CG1058	ripped pocket	477	1396	102	375	**78.62**
plate15-G05	CG4174	CG4174	407	1057	87	320	**78.62**
plate5-E12	CG1987	Rbp1-like	220	607	47	173	**78.64**
plate12-D09	CG3620	no receptor potential A	749	2064	160	589	**78.64**
plate7-F03	CG2658	CG2658	309	766	66	243	**78.64**

plate20-C01	CG4923	mitochondrial ribosomal protein L9	178	841	38	140	**78.65**
plate20-H05	CG5024	CG5024	89	355	19	70	**78.65**
plate20-B11	CG4920	easter	520	1554	111	409	**78.65**
plate1-G03	CG1212	p130CAS	595	1727	127	468	**78.66**
plate14-D10	CG3961	CG3961	164	466	35	129	**78.66**
plate10-F07	CG3279	CG3279	633	1586	135	498	**78.67**
plate1-B08	CG1065	Succinyl coenzyme A synthetase alpha subunit	333	805	71	262	**78.68**
plate16-E08	CG4322	moody	258	659	55	203	**78.68**
plate6-D03	CG2135	CG2135	319	802	68	251	**78.68**
plate20-E04	CG4961	I'm not dead yet 2	663	1741	141	522	**78.73**
plate4-C05	CG1744	chaoptic	287	1046	61	226	**78.75**
plate17-B07	CG4428	Autophagy-specific gene 4	160	435	34	126	**78.75**
plate2-A01	CG1264	labial	193	468	41	152	**78.76**
plate8-E03	CG2910	spenito	339	1017	72	267	**78.76**
plate20-F03	CG4977	kekkon-2	339	972	72	267	**78.76**
plate19-C06	CG4770	CG4770	179	542	38	141	**78.77**
plate12-H03	CG3689	CG3689	99	470	21	78	**78.79**
plate13-F03	CG3806	eIF2B-epsilon	66	139	14	52	**78.79**
plate1-H07	CG32297	-	217	498	46	171	**78.80**
plate18-G04	CG4683	CG4683	184	488	39	145	**78.80**
plate9-A05	CG2995	-	486	1284	103	383	**78.81**
plate12-B06	CG3552	CG3552	486	1218	103	383	**78.81**
plate10-D06	CG3231	something that sticks like glue	236	797	50	186	**78.81**
plate10-F08	CG3281	CG3281	340	951	72	268	**78.82**
plate20-G02	CG4998	CG4998	529	1619	112	417	**78.83**
plate15-G08	CG4181	Glutathione S transferase D2	326	868	69	257	**78.83**
plate11-C04	CG3388	gooseberry	671	1735	142	529	**78.84**
plate8-F04	CG2930	CG2930	416	918	88	328	**78.85**
plate14-G03	CG4012	genghis khan	539	1492	114	425	**78.85**
plate16-B09	CG4260	alpha-Adaptin	227	641	48	179	**78.85**
plate20-G05	CG5001	CG5001	298	797	63	235	**78.86**
plate5-B03	CG1895	Cyp28c1	123	334	26	97	**78.86**
plate10-E02	CG3251	CG3251	634	1890	134	500	**78.86**

plate19-F02	CG4826	-	336	921	71	265	**78.87**
plate2-D08	CG1381	CG1381	71	205	15	56	**78.87**
plate18-C07	CG4613	CG4613	412	1116	87	325	**78.88**
plate2-G09	CG1453	Klp10A	90	269	19	71	**78.89**
plate18-D06	CG4630	CG4630	289	896	61	228	**78.89**
plate17-E07	CG4495	CG4495	237	716	50	187	**78.90**
plate10-A02	CG3159	Excitatory amino acid transporter 2	939	2673	198	741	**78.91**
plate20-B03	CG4908	CG4908	204	655	43	161	**78.92**
plate4-F03	CG1800	partner of drosha	337	950	71	266	**78.93**
plate16-B03	CG4241	alternative testis transcripts open reading frame A	114	315	24	90	**78.95**
plate19-D08	CG4798	lethal (2) k01209	266	735	56	210	**78.95**
plate20-D07	CG4947	tRNA-guanine transglycosylase	328	1071	69	259	**78.96**
plate11-F04	CG3458	Topoisomerase 3beta	599	1822	126	473	**78.96**
plate13-A08	CG3710	RNA polymerase II elongation factor	271	756	57	214	**78.97**
plate10-G02	CG3290	CG3290	1598	4023	336	1262	**78.97**
plate2-H03	CG1464	eyeless	333	906	70	263	**78.98**
plate18-C04	CG4610	CG4610	119	365	25	94	**78.99**
plate10-B11	CG3194	CG3194	400	930	84	316	**79.00**
plate20-F04	CG4978	Minichromosome maintenance 7	405	1319	85	320	**79.01**
plate8-A08	CG2791	CG2791	353	884	74	279	**79.04**
plate8-E05	CG2914	Ets at 21C	1050	2519	220	830	**79.05**
plate17-E08	CG4496	CG4496	105	285	22	83	**79.05**
plate18-F01	CG4660	CG4660	148	670	31	117	**79.05**
plate2-B12	CG1332	CG1332	172	415	36	136	**79.07**
plate2-C12	CG1362	cdc2-related-kinase	86	202	18	68	**79.07**
plate12-B07	CG3560	CG3560	540	1532	113	427	**79.07**
plate18-F06	CG31738	CG42389	411	1148	86	325	**79.08**
plate12-E02	CG3630	CG3630	545	1447	114	431	**79.08**
plate13-E04	CG3790	CG3790	483	1197	101	382	**79.09**
plate1-F04	CG1147	neuropeptide F receptor	440	1205	92	348	**79.09**
plate5-F11	CG2017	CG2017	110	391	23	87	**79.09**
plate11-B07	CG3365	drongo	177	559	37	140	**79.10**
plate9-A06	CG32702	CG32702	244	684	51	193	**79.10**

plate7-H07	CG2736	CG2736	1067	2440	223	844	**79.10**
plate15-C08	CG4098	CG4098	450	1281	94	356	**79.11**
plate18-A08	CG4582	CG4582	91	278	19	72	**79.12**
plate16-A08	CG4225	CG4225	264	792	55	209	**79.17**
plate1-H02	CG1241	Autophagy-specific gene 2	418	1197	87	331	**79.19**
plate12-E06	CG3634	CG3634	721	2000	150	571	**79.20**
plate10-F01	CG3269	Rab-protein 2	1024	2250	213	811	**79.20**
plate1-A06	CG1028	Antennapedia	101	305	21	80	**79.21**
plate14-C01	CG3929	deltex	77	221	16	61	**79.22**
plate13-A07	CG3709	CG3709	53	179	11	42	**79.25**
plate17-D03	CG4463	Heat shock protein 23	434	1269	90	344	**79.26**
plate19-F06	CG4830	CG4830	381	1256	79	302	**79.27**
plate12-G08	CG3671	Malvolio	685	1900	142	543	**79.27**
plate7-A12	CG32703	CG32703	275	916	57	218	**79.27**
plate17-D05	CG4466	Heat shock protein 27	222	678	46	176	**79.28**
plate19-E01	CG4804	CG4804	140	437	29	111	**79.29**
plate17-E04	CG4486	Cytochrome P450-9b2	449	1355	93	356	**79.29**
plate5-C02	CG1916	Wnt oncogene analog 2	522	1789	108	414	**79.31**
plate9-A01	CG2989	Cht6	290	747	60	230	**79.31**
plate20-D04	CG4943	lethal with a checkpoint kinase	324	960	67	257	**79.32**
plate10-B02	CG3178	Recombination repair protein 1	561	1680	116	445	**79.32**
plate19-E05	CG4815	CG4815	329	901	68	261	**79.33**
plate10-A07	CG3166	anterior open	242	689	50	192	**79.34**
plate4-G10	CG1838	myoglianin	213	605	44	169	**79.34**
plate3-G10	CG1647	CG1647	155	509	32	123	**79.35**
plate8-C08	CG2864	Poly(ADP-ribose) glycohydrolase	407	1077	84	323	**79.36**
plate19-A06	CG4729	CG4729	126	507	26	100	**79.37**
plate14-E05	CG3972	Cytochrome P450-4g1	834	2207	172	662	**79.38**
plate14-C04	CG3936	Notch	388	934	80	308	**79.38**
plate11-D10	CG3422	Proteasome 28kD subunit 1	301	924	62	239	**79.40**
plate1-E11	CG1134	CG1134	68	246	14	54	**79.41**
plate1-G11	CG1234	CG1234	374	1030	77	297	**79.41**
plate20-C03	CG4925	CG4925	306	1040	63	243	**79.41**

plate5-C12	CG1944	Cyp4p2	209	500	43	166	**79.43**
plate8-C03	CG2857	CG2857	593	1428	122	471	**79.43**
plate10-A12	CG3173	CG3173	316	946	65	251	**79.43**
plate12-E04	CG3632	CG3632	530	1489	109	421	**79.43**
plate20-G07	CG5004	CG5004	501	1530	103	398	**79.44**
plate17-G11	CG4552	CG4552	180	543	37	143	**79.44**
plate2-B06	CG1317	CG1317	326	836	67	259	**79.45**
plate13-B06	CG3730	capsuleen	185	507	38	147	**79.46**
plate6-B10	CG2107	CG2107	224	619	46	178	**79.46**
plate4-D04	CG1762	beta[nu] integrin	117	392	24	93	**79.49**
plate13-E03	CG3788	CG3788	117	292	24	93	**79.49**
plate20-B05	CG4912	eEF1delta	234	789	48	186	**79.49**
plate10-B06	CG3182	seizure	239	696	49	190	**79.50**
plate15-A10	CG4064	CG4064	200	670	41	159	**79.50**
plate18-C08	CG4615	CG4615	361	977	74	287	**79.50**
plate4-F05	CG1804	kek6	166	634	34	132	**79.52**
plate5-E04	CG1972	CG1972	464	1610	95	369	**79.53**
plate10-A10	CG3169	Spt3	430	1258	88	342	**79.53**
plate10-A01	CG3158	spindle E	303	957	62	241	**79.54**
plate4-D08	CG1768	diaphanous	308	999	63	245	**79.55**
plate5-A05	CG1883	Ribosomal protein S7	132	492	27	105	**79.55**
plate5-H03	CG2060	Cytochrome P450-4e2	44	155	9	35	**79.55**
plate5-A01	CG1871	enhancer of rudimentary	489	1243	100	389	**79.55**
plate11-A04	CG3333	Nucleolar protein at 60B	225	855	46	179	**79.56**
plate2-B07	CG1318	Hexosaminidase 1	230	662	47	183	**79.57**
plate6-B09	CG2105	Corin	191	654	39	152	**79.58**
plate6-D11	CG2151	Thioredoxin reductase-1	49	142	10	39	**79.59**
plate14-E01	CG3966	neither inactivation nor afterpotential A	196	516	40	156	**79.59**
plate16-A02	CG4212	Rab-protein 14	49	128	10	39	**79.59**
plate20-D12	CG4954	eIF3-S8	49	213	10	39	**79.59**
plate7-G02	CG2681	CG2681	299	738	61	238	**79.60**
plate5-D02	CG1946	CG1946	358	1256	73	285	**79.61**
plate2-H02	CG1463	CG1463	216	676	44	172	**79.63**

plate19-C11	CG4781	CG4781	825	2445	168	657	**79.64**
plate8-H03	CG2977	innexin 7	1444	3584	294	1150	**79.64**
plate3-B03	CG1513	CG1513	447	1251	91	356	**79.64**
plate10-A06	CG3165	CG3165	457	1317	93	364	**79.65**
plate9-A09	CG3003	CG3003	182	492	37	145	**79.67**
plate1-E02	CG1119	Germ line transcription factor 1	305	1328	62	243	**79.67**
plate17-E02	CG4484	CG4484	187	589	38	149	**79.68**
plate2-E04	CG1404	ran	128	434	26	102	**79.69**
plate4-B02	CG1703	CG1703	128	431	26	102	**79.69**
plate10-E11	CG3267	CG3267	458	1207	93	365	**79.69**
plate8-F06	CG2932	Bteb2	330	925	67	263	**79.70**
plate17-D01	CG4459	CG4459	202	581	41	161	**79.70**
plate8-A02	CG2774	CG2774	138	382	28	110	**79.71**
plate13-F09	CG3815	CG3815	321	942	65	256	**79.75**
plate8-B05	CG2831	hoepel1	820	1998	166	654	**79.76**
plate20-A11	CG4903	Misexpression suppressor of ras 4	89	329	18	71	**79.78**
plate13-C01	CG3738	Cyclin-dependent kinase subunit 30A	277	656	56	221	**79.78**
plate4-E03	CG1782	Ubiquitin activating enzyme 1	203	793	41	162	**79.80**
plate9-C10	CG30194	CG30194	307	860	62	245	**79.80**
plate16-F07	CG4336	roughex	307	889	62	245	**79.80**
plate19-D06	CG4796	nocturnin	322	908	65	257	**79.81**
plate10-D01	CG3224	CG3224	441	1087	89	352	**79.82**
plate2-D07	CG1380	sugar transporter 4	223	683	45	178	**79.82**
plate12-H11	CG3701	CG3701	119	274	24	95	**79.83**
plate16-H02	CG4374	CG4374	119	429	24	95	**79.83**
plate11-A10	CG3350	bigmax	124	427	25	99	**79.84**
plate16-G02	CG4351	CG4351	248	784	50	198	**79.84**
plate8-C05	CG2859	TBP-associated factor 10	382	1071	77	305	**79.84**
plate11-E01	CG3425	Type III alcohol dehydrogenase	556	1486	112	444	**79.86**
plate6-F12	CG2202	CG2202	169	413	34	135	**79.88**
plate7-H06	CG2727	epithelial membrane protein	343	854	69	274	**79.88**
plate7-B07	CG2346	FMRFamide-related	174	425	35	139	**79.89**
plate7-D04	CG2528	CG2528	179	518	36	143	**79.89**

plate20-E12	CG4973	CG4973	184	629	37	147	**79.89**
plate18-G06	CG4685	CG4685	194	637	39	155	**79.90**
plate20-C02	CG4924	icln	423	1224	85	338	**79.91**
plate3-D07	CG1571	CG1571	468	1414	94	374	**79.91**
plate10-G03	CG3291	pacman	249	719	50	199	**79.92**
plate4-G03	CG1826	CG1826	304	1008	61	243	**79.93**
plate1-H01	CG1239	CG1239	628	1913	126	502	**79.94**
plate20-F02	CG4976	Mes-4	454	1315	91	363	**79.96**
plate3-D01	CG1550	CG1550	35	150	7	28	**80.00**
plate4-F08	CG1812	CG1812	165	492	33	132	**80.00**
plate5-D01	CG1945	fat facets	205	667	41	164	**80.00**
plate6-F07	CG2191	Sodium-dependent multivitamin transporter	105	289	21	84	**80.00**
plate7-D02	CG2522	GTP-binding protein	345	898	69	276	**80.00**
plate1-H06	CG32297	-	70	243	14	56	**80.00**
plate13-D08	CG3774	CG3774	420	1284	84	336	**80.00**
plate15-B04	CG4071	Vps20	75	596	15	60	**80.00**
plate15-C07	CG4097	Proteasome 26kD subunit	85	286	17	68	**80.00**
plate16-B11	CG4261	Helicase 89B	45	178	9	36	**80.00**
plate16-H01	CG4373	Cyp6d2	15	72	3	12	**80.00**
plate17-A10	CG4408	CG4408	45	140	9	36	**80.00**
plate18-A10	CG4584	Deoxyuridine triphosphatase	70	258	14	56	**80.00**
plate18-H04	CG4705	CG4705	5	19	1	4	**80.00**
plate12-D06	CG3615	Autophagy-specific gene 9	886	2471	177	709	**80.02**
plate11-D03	CG3409	CG3409	451	1261	90	361	**80.04**
plate8-F05	CG2931	CG2931	782	2255	156	626	**80.05**
plate18-E05	CG4648	CG42399	351	1014	70	281	**80.06**
plate8-D02	CG2889	CG2889	336	909	67	269	**80.06**
plate18-E03	CG4645	CG4645	331	967	66	265	**80.06**
plate2-C08	CG1347	CG1347	241	712	48	193	**80.08**
plate20-G10	CG5013	CG5013	397	1091	79	318	**80.10**
plate4-A09	CG1695	CG1695	176	612	35	141	**80.11**
plate5-H09	CG2070	CG2070	307	942	61	246	**80.13**
plate8-D07	CG2901	CG2901	544	1461	108	436	**80.15**

plate18-F04	CG4665	Dihydropteridine reductase	136	435	27	109	**80.15**
plate10-F09	CG3282	CG30291	353	1030	70	283	**80.17**
plate3-E07	CG1600	CG1600	343	1058	68	275	**80.17**
plate20-C07	CG32031	Arginine kinase	318	1100	63	255	**80.19**
plate12-F04	CG3647	shuttle craft	212	563	42	170	**80.19**
plate19-E03	CG4810	CG4810	212	765	42	170	**80.19**
plate9-G04	CG3117	CG3117	475	1153	94	381	**80.21**
plate12-A03	CG3523	CG3523	187	539	37	150	**80.21**
plate15-D09	CG4120	Cyp12c1	273	743	54	219	**80.22**
plate18-A11	CG4586	CG4586	91	268	18	73	**80.22**
plate7-B10	CG2397	Cyp6a13	354	855	70	284	**80.23**
plate2-C02	CG1340	CG1340	349	957	69	280	**80.23**
plate18-B05	CG4592	CG4592	86	255	17	69	**80.23**
plate20-H03	CG5022	CG5022	339	1127	67	272	**80.24**
plate11-E06	CG3434	CG3434	420	1108	83	337	**80.24**
plate16-D08	CG4300	CG4300	680	1890	134	546	**80.29**
plate8-E04	CG2913	yin	406	1073	80	326	**80.30**
plate8-E07	CG2917	Origin recognition complex subunit 4	559	1496	110	449	**80.32**
plate16-D03	CG4289	CG4289	183	632	36	147	**80.33**
plate4-C04	CG1743	Glutamine synthetase 2	117	342	23	94	**80.34**
plate8-D11	CG2905	Nipped-A	117	541	23	94	**80.34**
plate20-E06	CG4965	twine	351	1043	69	282	**80.34**
plate12-F09	CG3654	CG3654	840	2209	165	675	**80.36**
plate19-B05	CG4752	CG4752	336	996	66	270	**80.36**
plate1-B06	CG1063	Inositol 1,4,5,-tris-phosphate receptor	107	313	21	86	**80.37**
plate2-D05	CG1378	tailless	214	591	42	172	**80.37**
plate14-D04	CG3950	-	816	1918	160	656	**80.39**
plate16-F09	CG4341	CG4341	51	184	10	41	**80.39**
plate1-C08	CG1088	Vacuolar H[+]-ATPase 26kD E subunit	643	1708	126	517	**80.40**
plate16-D10	CG4301	CG4301	337	1023	66	271	**80.42**
plate10-A05	CG3162	CG3162	332	926	65	267	**80.42**
plate20-C10	CG4934	brainiac	378	1224	74	304	**80.42**
plate20-C09	CG4933	CG4933	562	1847	110	452	**80.43**

plate6-C07	CG2120	CG2120	46	183	9	37	**80.43**
plate13-D03	CG3764	CG3764	138	370	27	111	**80.43**
plate20-G03	CG4999	Tetraspanin 66E	670	1989	131	539	**80.45**
plate17-G03	CG4542	CG4542	445	1319	87	358	**80.45**
plate4-H01	CG1842	Dynein heavy chain at 89D	174	465	34	140	**80.46**
plate5-D04	CG1951	CG1951	374	1138	73	301	**80.48**
plate12-E09	CG3638	CG3638	364	1204	71	293	**80.49**
plate18-E08	CG4653	CG4653	282	801	55	227	**80.50**
plate3-D10	CG1582	CG1582	231	681	45	186	**80.52**
plate7-H01	CG2712	CG2712	77	216	15	62	**80.52**
plate14-F05	CG3994	-	1001	2778	195	806	**80.52**
plate20-A01	CG4887	CG4887	77	303	15	62	**80.52**
plate17-C03	CG4439	CG4439	190	613	37	153	**80.53**
plate15-D01	CG4107	Pcaf	185	558	36	149	**80.54**
plate2-G11	CG1455	Calcineurin A1	144	498	28	116	**80.56**
plate13-D09	CG3775	CG3775	494	1385	96	398	**80.57**
plate12-D12	CG3626	CG3626	67	183	13	54	**80.60**
plate20-B04	CG4909	Plenty of SH3s	263	958	51	212	**80.61**
plate10-G06	CG3295	CG3295	392	1039	76	316	**80.61**
plate15-A03	CG4051	egalitarian	98	368	19	79	**80.61**
plate10-A11	CG3172	twinfilin	552	1491	107	445	**80.62**
plate2-B01	CG1308	CG1308	258	672	50	208	**80.62**
plate20-A09	CG4901	CG4901	258	853	50	208	**80.62**
plate13-D01	CG32648	Phosphodiesterase 9	160	423	31	129	**80.63**
plate3-H04	CG1663	CG1663	191	521	37	154	**80.63**
plate1-C03	CG1078	CG1078	222	804	43	179	**80.63**
plate20-E07	CG4966	CG4966	687	1976	133	554	**80.64**
plate20-G06	CG5002	CG5002	951	2794	184	767	**80.65**
plate4-A01	CG1681	CG1681	181	609	35	146	**80.66**
plate8-D06	CG2899	kinase suppressor of ras	119	346	23	96	**80.67**
plate14-H03	CG4033	RNA polymerase I 135kD subunit	259	740	50	209	**80.69**
plate18-D02	CG4625	Dihydroxyacetone phosphate acyltransferase	316	907	61	255	**80.70**
plate8-A12	CG2812	CG2812	114	356	22	92	**80.70**

plate19-D05	CG31213	CG31213	425	1221	82	343	**80.71**
plate20-F06	CG4980	CG4980	852	2470	164	688	**80.75**
plate1-E07	CG1130	scratch	130	453	25	105	**80.77**
plate5-C08	CG1938	Dlic2	130	430	25	105	**80.77**
plate8-C02	CG2856	Mnt	312	754	60	252	**80.77**
plate19-E07	CG4817	Structure specific recognition protein	416	1569	80	336	**80.77**
plate17-G01	CG4536	inactive	73	327	14	59	**80.82**
plate10-B09	CG3192	CG3192	339	907	65	274	**80.83**
plate11-C03	CG30277	Organic anion transporting polypeptide 58Da	360	1072	69	291	**80.83**
plate16-F04	CG4332	CG4332	240	738	46	194	**80.83**
plate15-B10	CG4083	Mo25	402	1221	77	325	**80.85**
plate5-G02	CG2025	CG2025	277	978	53	224	**80.87**
plate12-B05	CG30092	jitterbug	361	882	69	292	**80.89**
plate15-B05	CG4074	CG4074	314	822	60	254	**80.89**
plate1-A08	CG1031	alpha-Esterase-1	89	272	17	72	**80.90**
plate14-E02	CG3967	CG3967	529	1474	101	428	**80.91**
plate18-B01	CG31711	-	110	402	21	89	**80.91**
plate6-A08	CG2088	CG30492	241	643	46	195	**80.91**
plate6-C01	CG2111	CG2111	262	751	50	212	**80.92**
plate1-F03	CG1142	CG1142	477	1554	91	386	**80.92**
plate6-E08	CG2168	Ribosomal protein S3A	147	685	28	119	**80.95**
plate13-G05	CG3841	CG3841	147	431	28	119	**80.95**
plate15-B09	CG4082	Minichromosome maintenance 5	147	449	28	119	**80.95**
plate15-D08	CG4119	CG4119	126	411	24	102	**80.95**
plate8-A05	CG2788	Dorothy	689	1864	131	558	**80.99**
plate2-B08	CG1319	CG1319	100	270	19	81	**81.00**
plate17-G04	CG4545	Serotonin transporter	200	637	38	162	**81.00**
plate5-H10	CG2071	Serine protease 6	358	979	68	290	**81.01**
plate5-H04	CG2061	CG2061	316	1046	60	256	**81.01**
plate5-H08	CG2069	Oseg4	174	552	33	141	**81.03**
plate18-D08	CG4634	Nucleosome remodeling factor - 38kD	174	666	33	141	**81.03**
plate9-A03	CG2993	CG2993	327	1018	62	265	**81.04**
plate10-B12	CG3198	CG3198	459	1194	87	372	**81.05**

plate5-G10	CG2052	CG2052	201	648	38	163	**81.09**
plate3-G04	CG1638	CG1638	164	422	31	133	**81.10**
plate19-B02	CG4749	CG4749	127	345	24	103	**81.10**
plate16-A11	CG4236	Chromatin assembly factor 1 subunit	90	274	17	73	**81.11**
plate20-F08	CG4988	CG4988	556	1738	105	451	**81.12**
plate2-D02	CG1371	CG1371	249	743	47	202	**81.12**
plate18-G05	CG4684	nervous wreck	281	854	53	228	**81.14**
plate15-F02	CG31256	Brf	175	499	33	142	**81.14**
plate13-A06	CG3708	CG3708	175	399	33	142	**81.14**
plate17-E09	CG4498	nicotinic Acetylcholine Receptor alpha 34E	175	528	33	142	**81.14**
plate15-A08	CG4061	CG4061	244	744	46	198	**81.15**
plate13-E12	CG3799	Ephexin	191	534	36	155	**81.15**
plate18-A09	CG4583	ire-1	69	275	13	56	**81.16**
plate14-E06	CG3973	CG3973	393	1024	74	319	**81.17**
plate2-A07	CG1287	CG1287	85	274	16	69	**81.18**
plate18-D12	CG31365	CG31365	186	569	35	151	**81.18**
plate16-A07	CG31183	CG31183	202	585	38	164	**81.19**
plate4-G05	CG1828	dre4	250	715	47	203	**81.20**
plate3-B07	CG1517	narrow abdomen	133	403	25	108	**81.20**
plate5-A07	CG1885	CG1885	266	807	50	216	**81.20**
plate10-C10	CG3219	Klp59C	463	1312	87	376	**81.21**
plate8-E06	CG2915	CG2915	261	703	49	212	**81.23**
plate10-B03	CG32048	CG32048	341	847	64	277	**81.23**
plate2-E08	CG1409	CG1409	176	415	33	143	**81.25**
plate5-G09	CG2051	CG2051	192	530	36	156	**81.25**
plate14-A08	CG3891	CG3891	64	220	12	52	**81.25**
plate17-D08	CG4475	Imaginal disc growth factor 2	320	933	60	260	**81.25**
plate11-D11	CG3423	Stromalin	315	825	59	256	**81.27**
plate20-H07	CG5026	CG5026	299	819	56	243	**81.27**
plate18-D04	CG4627	CG4627	203	510	38	165	**81.28**
plate20-B01	CG4905	Syntrophin-like 2	155	538	29	126	**81.29**
plate7-A08	CG2287	Hyperkinetic	139	367	26	113	**81.29**
plate8-G04	CG2956	twist	278	768	52	226	**81.29**

plate17-F01	CG4509	Cad86C	107	395	20	87	**81.31**
plate3-G07	CG1644	Cyp6t1	150	563	28	122	**81.33**
plate6-D05	CG2137	CG2137	150	492	28	122	**81.33**
plate19-F04	CG31293	recombination-defective	525	1490	98	427	**81.33**
plate12-C02	CG3578	bifid	375	1026	70	305	**81.33**
plate17-D06	CG4467	CG4467	134	429	25	109	**81.34**
plate1-G04	CG1213	CG1213	445	1316	83	362	**81.35**
plate6-A01	CG32488	CG32488	86	251	16	70	**81.40**
plate20-B06	CG4914	CG4914	490	1521	91	399	**81.43**
plate6-B08	CG2104	CG2104	264	675	49	215	**81.44**
plate5-C06	CG30337	bruchpilot	248	793	46	202	**81.45**
plate3-H10	CG1673	CG1673	27	72	5	22	**81.48**
plate16-G10	CG4365	CG4365	324	978	60	264	**81.48**
plate16-H04	CG4379	cAMP-dependent protein kinase 1	81	508	15	66	**81.48**
plate17-C04	CG4445	polypeptide GalNAc transferase 3	243	705	45	198	**81.48**
plate19-C07	CG4774	CG4774	746	2297	138	608	**81.50**
plate5-B06	CG1903	strawberry notch	173	644	32	141	**81.50**
plate6-A07	CG2087	pancreatic eIF-2alpha kinase	146	425	27	119	**81.51**
plate18-C02	CG4608	branchless	211	707	39	172	**81.52**
plate16-F02	CG4330	CG4330	249	843	46	203	**81.53**
plate16-B05	CG4252	meiotic 41	287	874	53	234	**81.53**
plate5-A04	CG1882	CG1882	282	816	52	230	**81.56**
plate11-A05	CG3335	CG3335	141	459	26	115	**81.56**
plate11-D01	CG3407	CG3407	537	1485	99	438	**81.56**
plate1-D10	CG1109	CG1109	152	514	28	124	**81.58**
plate20-H02	CG5021	CG5021	342	1156	63	279	**81.58**
plate6-E10	CG2174	unconventional myosin class XV	201	621	37	164	**81.59**
plate19-C03	CG4766	CG4766	201	607	37	164	**81.59**
plate20-A07	CG4899	Photoreceptor dehydrogenase	250	862	46	204	**81.60**
plate13-B12	CG3736	okra	87	237	16	71	**81.61**
plate18-H10	CG4719	tankyrase	87	253	16	71	**81.61**
plate2-F04	CG1420	Slu7	60	182	11	49	**81.67**
plate9-A04	CG32701	lethal (1) G0320	344	947	63	281	**81.69**

plate14-C02	CG3931	Rrp4	344	934	63	281	**81.69**
plate3-D05	CG1560	myospheroid	350	1263	64	286	**81.71**
plate3-D03	CG1555	cinnabar	547	1818	100	447	**81.72**
plate9-H07	CG3143	forkhead box, sub-group O	93	331	17	76	**81.72**
plate17-F03	CG4520	CG4520	476	1377	87	389	**81.72**
plate13-H10	CG3875	CG3875	104	316	19	85	**81.73**
plate18-E04	CG4647	mitochondrial ribosomal protein L49	208	686	38	170	**81.73**
plate2-E03	CG1403	Septin-1	356	1200	65	291	**81.74**
plate12-E05	CG3633	mitochondrial ribosomal protein S29	340	1090	62	278	**81.76**
plate5-E09	CG1981	Thd1	236	713	43	193	**81.78**
plate3-D04	CG1559	Upf1	187	694	34	153	**81.82**
plate8-E09	CG2919	asterless	220	606	40	180	**81.82**
plate10-H07	CG3320	Rab-protein 1	561	1958	102	459	**81.82**
plate20-G04	CG5000	mini spindles	429	1212	78	351	**81.82**
plate16-C09	CG4276	arouser	248	703	45	203	**81.85**
plate15-F09	CG4164	CG4164	116	326	21	95	**81.90**
plate3-E03	CG1594	hopscotch	326	1026	59	267	**81.90**
plate3-H03	CG1659	unc-119	199	650	36	163	**81.91**
plate5-E05	CG1973	CG1973	293	1008	53	240	**81.91**
plate2-B05	CG1316	CG1316	166	481	30	136	**81.93**
plate5-C01	CG1913	alpha-Tubulin at 84B	249	1203	45	204	**81.93**
plate5-G01	CG2023	CG2023	321	871	58	263	**81.93**
plate15-A01	CG4049	CG4049	72	274	13	59	**81.94**
plate19-H09	CG4878	eIF3-S9	72	230	13	59	**81.94**
plate6-E02	CG2158	CG2158	410	1110	74	336	**81.95**
plate6-D01	CG2129	CG2129	133	371	24	109	**81.95**
plate13-G07	CG3843	Ribosomal protein L10Aa	233	629	42	191	**81.97**
plate20-G08	CG5009	CG5009	577	1833	104	473	**81.98**
plate16-G05	CG4356	muscarinic Acetylcholine Receptor 60C	322	913	58	264	**81.99**
plate9-A02	CG2990	CG2990	428	1161	77	351	**82.01**
plate10-F02	CG3270	CG3270	395	1265	71	324	**82.03**
plate6-A09	CG2091	CG2091	540	1366	97	443	**82.04**
plate5-E08	CG1977	alpha Spectrin	117	384	21	96	**82.05**

plate5-B04	CG1896	CG1896	173	454	31	142	**82.08**
plate3-A10	CG1504	CG1504	67	281	12	55	**82.09**
plate11-G01	CG3478	pickpocket	151	654	27	124	**82.12**
plate20-D06	CG4945	CG4945	571	1883	102	469	**82.14**
plate13-H06	CG3869	Mitochondrial assembly regulatory factor	28	73	5	23	**82.14**
plate17-F04	CG4521	methuselah-like 1	129	416	23	106	**82.17**
plate20-D05	CG4944	ciboulot	129	421	23	106	**82.17**
plate6-A12	CG2095	sec8	174	598	31	143	**82.18**
plate7-G06	CG2691	CG2691	320	844	57	263	**82.19**
plate4-D02	CG1759	caudal	292	1093	52	240	**82.19**
plate18-D03	CG4626	frizzled 4	90	266	16	74	**82.22**
plate1-B12	CG1072	Arrowhead	231	736	41	190	**82.25**
plate17-B04	CG4421	Glutathione S transferase D8	79	212	14	65	**82.28**
plate10-F03	CG3271	CG3271	175	500	31	144	**82.29**
plate11-F07	CG3461	prune	543	1369	96	447	**82.32**
plate5-D06	CG1956	Roughened	198	576	35	163	**82.32**
plate20-G01	CG4996	CG4996	317	1060	56	261	**82.33**
plate15-C05	CG4095	CG4095	419	1160	74	345	**82.34**
plate6-C12	CG2128	Hdac3	102	275	18	84	**82.35**
plate13-C12	CG3759	CG3759	102	274	18	84	**82.35**
plate17-E10	CG4500	CG4500	34	98	6	28	**82.35**
plate13-B04	CG3725	Calcium ATPase at 60A	284	843	50	234	**82.39**
plate14-A05	CG3886	Posterior sex combs	182	724	32	150	**82.42**
plate4-B07	CG1716	Set2	154	428	27	127	**82.47**
plate8-D03	CG2890	Protein phosphatase 4 regulatory subunit 2-related protein	348	1065	61	287	**82.47**
plate17-G09	CG4550	neither inactivation nor afterpotential E	97	235	17	80	**82.47**
plate16-F03	CG32645	CG32645	234	733	41	193	**82.48**
plate9-F07	CG32697	lethal (1) G0232	274	715	48	226	**82.48**
plate12-B01	CG3540	Cyp4d14	314	980	55	259	**82.48**
plate5-E06	CG1975	Rep2	229	712	40	189	**82.53**
plate16-C07	CG4271	CG4271	63	212	11	52	**82.54**
plate15-C09	CG4099	Scavenger receptor class C, type I	212	662	37	175	**82.55**
plate3-G06	CG1640	CG1640	86	249	15	71	**82.56**

plate5-D07	CG1957	CG1957	218	729	38	180	**82.57**
plate5-C03	CG1921	sprouty	241	852	42	199	**82.57**
plate8-F07	CG2937	mitochondrial ribosomal protein S2	477	1485	83	394	**82.60**
plate8-G10	CG2970	CG2970	161	455	28	133	**82.61**
plate16-B12	CG4262	embryonic lethal abnormal vision	69	179	12	57	**82.61**
plate10-D02	CG3225	CG3225	656	2047	114	542	**82.62**
plate17-E01	CG4483	CG4483	121	380	21	100	**82.64**
plate18-E07	CG4651	Ribosomal protein L13	294	890	51	243	**82.65**
plate16-B07	CG4257	Signal-transducer and activator of transcription protein at 92E	248	735	43	205	**82.66**
plate14-D03	CG3949	hoi-polloi	75	363	13	62	**82.67**
plate19-C10	CG4780	CG4780	225	695	39	186	**82.67**
plate17-B02	CG4415	CG4415	127	350	22	105	**82.68**
plate10-D09	CG3239	CG3239	485	1331	84	401	**82.68**
plate12-F03	CG3646	frizzled	631	1700	109	522	**82.73**
plate13-A02	CG3704	CG3704	139	356	24	115	**82.73**
plate20-D03	CG4942	CG4942	168	580	29	139	**82.74**
plate11-F02	CG3455	Rpt4	197	762	34	163	**82.74**
plate19-G06	CG4849	CG4849	197	828	34	163	**82.74**
plate14-H12	CG4045	CG4045	29	71	5	24	**82.76**
plate17-C02	CG4438	CG4438	180	546	31	149	**82.78**
plate3-F07	CG1625	CG1625	93	300	16	77	**82.80**
plate14-G04	CG4013	Smrter	291	943	50	241	**82.82**
plate16-E09	CG4324	CG4324	99	319	17	82	**82.83**
plate7-B03	CG2328	even skipped	233	583	40	193	**82.83**
plate19-G07	CG4852	severas	513	1501	88	425	**82.85**
plate16-B02	CG4239	CG4239	105	381	18	87	**82.86**
plate16-B06	CG4254	twinstar	140	494	24	116	**82.86**
plate17-D02	CG4462	CG4462	374	1225	64	310	**82.89**
plate5-F10	CG2014	CG2014	339	1094	58	281	**82.89**
plate20-A08	CG4900	Iron regulatory protein 1A	193	602	33	160	**82.90**
plate17-C08	CG4450	-	141	469	24	117	**82.98**
plate5-B02	CG1894	CG1894	253	969	43	210	**83.00**
plate6-B02	CG2097	CG2097	153	467	26	127	**83.01**

plate16-A10	CG4226	Glutamate receptor IIC	265	766	45	220	**83.02**
plate8-G09	CG2969	ABC transporter expressed in trachea	112	287	19	93	**83.04**
plate20-A02	CG4889	wingless	142	466	24	118	**83.10**
plate16-E04	CG4317	Multiple inositol polyphosphate phosphatase 2	208	630	35	173	**83.17**
plate18-B12	CG4606	alpha-Man-IIb	113	319	19	94	**83.19**
plate10-D05	CG33123	CG33123	143	495	24	119	**83.22**
plate16-C04	CG4266	CG4266	304	1009	51	253	**83.22**
plate5-G04	CG2031	Hpr1	203	674	34	169	**83.25**
plate3-D09	CG1578	CG1578	102	354	17	85	**83.33**
plate6-G10	CG2241	CG2241	66	207	11	55	**83.33**
plate8-G05	CG2957	mitochondrial ribosomal protein S9	306	948	51	255	**83.33**
plate14-A10	CG3896	NADPH oxidase	132	484	22	110	**83.33**
plate14-H10	CG4042	CG4042	60	194	10	50	**83.33**
plate15-H04	CG4199	CG4199	6	57	1	5	**83.33**
plate17-C01	CG4437	Peptidoglycan recognition protein LF	138	582	23	115	**83.33**
plate17-H03	CG4557	CG4557	30	116	5	25	**83.33**
plate17-H06	CG4561	Tyrosyl-tRNA synthetase	6	23	1	5	**83.33**
plate19-G08	CG4853	CG4853	246	710	41	205	**83.33**
plate6-E05	CG2161	Regena	54	157	9	45	**83.33**
plate10-A03	CG3160	CG3160	427	1375	71	356	**83.37**
plate20-E05	CG4963	CG4963	507	1719	84	423	**83.43**
plate3-F02	CG1616	disc proliferation abnormal	151	618	25	126	**83.44**
plate20-F07	CG32778	CG32778	363	1490	60	303	**83.47**
plate6-H01	CG2246	CG2246	109	443	18	91	**83.49**
plate16-E02	CG4314	scarlet	212	703	35	177	**83.49**
plate19-H06	CG4871	Sialyltransferase	212	710	35	177	**83.49**
plate1-B10	CG1070	Alhambra	79	294	13	66	**83.54**
plate13-A10	CG3714	CG3714	146	502	24	122	**83.56**
plate7-A01	CG2263	CG2263	61	220	10	51	**83.61**
plate12-C03	CG3582	U2 small nuclear riboprotein auxiliary factor 38	232	715	38	194	**83.62**
plate16-E01	CG4313	CG4313	55	172	9	46	**83.64**
plate18-A07	CG4581	Thiolase	55	153	9	46	**83.64**
plate14-H05	CG4036	CG4036	288	1086	47	241	**83.68**

plate16-A06	CG4221	CG4221	190	716	31	159	**83.68**
plate20-E01	CG4956	CG4956	233	815	38	195	**83.69**
plate2-F03	CG1418	CG1418	184	577	30	154	**83.70**
plate8-B02	CG2819	PvuII-PstI homology 13	313	936	51	262	**83.71**
plate16-C02	CG4264	Heat shock protein cognate 4	43	184	7	36	**83.72**
plate16-D06	CG4293	CG4293	375	1185	61	314	**83.73**
plate18-A02	CG4573	CG4573	80	294	13	67	**83.75**
plate17-B03	CG4420	CG4420	228	728	37	191	**83.77**
plate3-G05	CG1639	lethal (1) 10Bb	74	284	12	62	**83.78**
plate20-C06	CG4928	CG4928	426	1415	69	357	**83.80**
plate15-H02	CG4193	deadhead	105	313	17	88	**83.81**
plate13-A09	CG3711	CG3711	161	518	26	135	**83.85**
plate5-C05	CG1924	CG1924	192	632	31	161	**83.85**
plate4-A07	CG1692	maroon-like	93	296	15	78	**83.87**
plate9-H01	CG3131	Dual oxidase	62	313	10	52	**83.87**
plate3-F04	CG1618	comatose	149	540	24	125	**83.89**
plate18-B03	CG4590	innexin 2	168	490	27	141	**83.93**
plate9-D06	CG3048	TNF-receptor-associated factor 4	193	543	31	162	**83.94**
plate18-A01	CG4572	CG4572	81	365	13	68	**83.95**
plate20-D02	CG4938	Seryl-tRNA synthetase	381	1288	61	320	**83.99**
plate20-A04	CG4894	Ca[2+]-channel protein alpha[[1]] subunit D	169	574	27	142	**84.02**
plate14-C12	CG3945	Rad9	69	214	11	58	**84.06**
plate20-E03	CG4960	CG4960	253	743	40	213	**84.19**
plate14-B06	CG3917	gamma-tubulin ring protein 84	272	836	43	229	**84.19**
plate1-H09	CG1250	sec23	19	65	3	16	**84.21**
plate7-A07	CG2286	NADH:ubiquinone reductase 75kD subunit precursor	57	222	9	48	**84.21**
plate8-A11	CG2808	-	114	317	18	96	**84.21**
plate18-C03	CG4609	failed axon connections	152	453	24	128	**84.21**
plate18-G12	CG4700	Sema-2a	19	61	3	16	**84.21**
plate18-H06	CG4707	CG4707	19	96	3	16	**84.21**
plate19-B01	CG4746	mab-21	89	274	14	75	**84.27**
plate15-F04	CG4158	worniu	70	342	11	59	**84.29**
plate16-H10	CG4386	CG4386	51	181	8	43	**84.31**

plate14-B01	CG3903	Gliotactin	185	514	29	156	**84.32**
plate16-G08	CG4364	CG4364	185	613	29	156	**84.32**
plate3-G02	CG1635	CG1635	192	676	30	162	**84.38**
plate13-H07	CG3870	chrowded	32	127	5	27	**84.38**
plate18-D01	CG4624	CG4624	32	143	5	27	**84.38**
plate13-C07	CG3751	Ribosomal protein S24	186	629	29	157	**84.41**
plate16-A04	CG4215	spellchecker1	187	623	29	158	**84.49**
plate2-E05	CG32604	lethal (1) G0007	208	761	32	176	**84.62**
plate3-C07	CG1539	tropomodulin	410	1280	63	347	**84.63**
plate18-C05	CG4611	CG4611	314	1026	48	266	**84.71**
plate5-A08	CG1886	ATP7	229	711	35	194	**84.72**
plate16-D01	CG4282	CG4282	72	207	11	61	**84.72**
plate15-G01	CG4168	CG4168	190	555	29	161	**84.74**
plate16-F08	CG4337	mitochondrial single stranded DNA-binding protein	118	361	18	100	**84.75**
plate13-H12	CG3878	CoRest	46	175	7	39	**84.78**
plate2-E06	CG1406	U2A	395	1531	60	335	**84.81**
plate15-G02	CG4169	-	349	1361	53	296	**84.81**
plate6-C05	CG2116	CG2116	112	303	17	95	**84.82**
plate5-G08	CG2049	Protein kinase related to protein kinase N	245	753	37	208	**84.90**
plate17-B01	CG4413	trade embargo	73	335	11	62	**84.93**
plate3-A08	CG1499	CG1499	273	961	41	232	**84.98**
plate13-H04	CG3862	CG3862	120	411	18	102	**85.00**
plate11-A02	CG3331	ebony	147	477	22	125	**85.03**
plate16-A05	CG4217	mitochondrial transcription factor A	107	398	16	91	**85.05**
plate13-B03	CG3724	Phosphogluconate dehydrogenase	67	199	10	57	**85.07**
plate19-A04	CG4725	CG4725	228	692	34	194	**85.09**
plate19-G03	CG4845	phagocyte signaling impaired	47	142	7	40	**85.11**
plate15-E11	CG4152	lethal (2) 35Df	101	270	15	86	**85.15**
plate3-E02	CG1591	REG	54	210	8	46	**85.19**
plate17-H01	CG32743	Smg1	27	173	4	23	**85.19**
plate13-F07	CG3811	Organic anion transporting polypeptide 30B	217	751	32	185	**85.25**
plate5-D05	CG1954	Protein C kinase 98E	319	1051	47	272	**85.27**
plate2-C03	CG1341	Rpt1	34	147	5	29	**85.29**

plate12-H01	CG30445	Tyrosine decarboxylase 1	102	471	15	87	**85.29**
plate16-G04	CG4355	ia2	245	808	36	209	**85.31**
plate1-H11	CG1258	pavarotti	109	433	16	93	**85.32**
plate5-B12	CG1912	Guanylyl cyclase alpha-subunit at 99B	164	627	24	140	**85.37**
plate15-H03	CG4196	CG4196	41	187	6	35	**85.37**
plate16-H09	CG4386	CG4386	82	257	12	70	**85.37**
plate10-F06	CG3278	-	130	352	19	111	**85.38**
plate19-A03	CG32574	Tweedlealpha	158	578	23	135	**85.44**
plate3-C09	CG1543	Tyramine beta hydroxylase	166	551	24	142	**85.54**
plate3-F03	CG32717	stardust	263	844	38	225	**85.55**
plate12-A12	CG3539	SLY-1 homologous	104	310	15	89	**85.58**
plate4-C07	CG1747	Sphingosine kinase 1	382	1239	55	327	**85.60**
plate17-A06	CG4402	lysyl oxidase-like 2	139	423	20	119	**85.61**
plate16-C05	CG4267	CG4267	285	916	41	244	**85.61**
plate3-H09	CG1671	CG1671	133	456	19	114	**85.71**
plate18-H01	CG4701	CG4701	7	60	1	6	**85.71**
plate19-A07	CG4730	CG4730	168	642	24	144	**85.71**
plate7-C03	CG2469	CG2469	98	393	14	84	**85.71**
plate5-E03	CG1970	CG1970	232	1044	33	199	**85.78**
plate12-D04	CG3612	bellwether	106	456	15	91	**85.85**
plate3-C02	CG1529	CG1529	198	748	28	170	**85.86**
plate5-B11	CG1911	CAP-D2 condensin subunit	128	623	18	110	**85.94**
plate6-B12	CG2109	mitochondrial ribosomal protein L44	121	379	17	104	**85.95**
plate16-E05	CG4318	CG4318	193	561	27	166	**86.01**
plate3-E09	CG1603	CG1603	94	274	13	81	**86.17**
plate16-A09	CG4233	Glutamate oxaloacetate transaminase 2	123	391	17	106	**86.18**
plate16-G12	CG4372	CG4372	29	120	4	25	**86.21**
plate19-H07	CG4872	CG4872	58	203	8	50	**86.21**
plate4-D07	CG1765	Ecdysone receptor	262	856	36	226	**86.26**
plate6-D09	CG2145	CG2145	102	277	14	88	**86.27**
plate18-E02	CG4644	CG4644	124	557	17	107	**86.29**
plate3-B09	CG1519	Proteasome alpha7 subunit	95	419	13	82	**86.32**
plate11-A03	CG3332	CG3332	205	677	28	177	**86.34**

plate17-A01	CG4393	CG4393	44	300	6	38	**86.36**
plate5-H07	CG2064	CG2064	221	674	30	191	**86.43**
plate5-B08	CG1906	alphabet	185	711	25	160	**86.49**
plate16-H08	CG4384	Peptidoglycan recognition protein LA	37	110	5	32	**86.49**
plate12-F01	CG3644	bicaudal	200	901	27	173	**86.50**
plate17-G08	CG32743	Smg1	156	606	21	135	**86.54**
plate19-C01	CG4761	knirps-like	83	262	11	72	**86.75**
plate20-D01	CG4937	RhoGAP15B	220	749	29	191	**86.82**
plate1-A03	CG1017	CG1017	175	795	23	152	**86.86**
plate6-B05	CG2101	mitochondrial ribosomal protein S35	138	462	18	120	**86.96**
plate12-F12	CG3658	CDC45L	62	229	8	54	**87.10**
plate1-A12	CG1049	CTP:phosphocholine cytidylyltransferase 1	156	592	20	136	**87.18**
plate16-D04	CG4290	CG4290	195	602	25	170	**87.18**
plate15-A05	CG4054	Fish-lips	47	208	6	41	**87.23**
plate13-F04	CG3808	CG3808	157	484	20	137	**87.26**
plate15-C01	CG4087	Ribosomal protein LP1	181	620	23	158	**87.29**
plate18-H03	CG4704	CG4704	71	450	9	62	**87.32**
plate14-A02	CG3881	GlcAT-S	150	474	19	131	**87.33**
plate18-G03	CG4679	CG4679	87	376	11	76	**87.36**
plate5-A03	CG1877	lin-19-like	208	1012	26	182	**87.50**
plate16-A03	CG4214	Syntaxin 5	56	212	7	49	**87.50**
plate18-G01	CG4677	lame duck	32	133	4	28	**87.50**
plate2-D04	CG32604	lethal (1) G0007	266	1040	33	233	**87.59**
plate3-D02	CG1554	RNA polymerase II 215kD subunit	81	478	10	71	**87.65**
plate11-H02	CG3497	Suppressor of Hairless	73	326	9	64	**87.67**
plate3-A02	CG1488	Cyp311a1	187	810	23	164	**87.70**
plate18-H07	CG4709	CG4709	49	1614	6	43	**87.76**
plate3-A01	CG1487	kurtz	131	513	16	115	**87.79**
plate10-B05	CG3181	Ts	280	1133	34	246	**87.86**
plate3-H06	CG1665	CG1665	116	365	14	102	**87.93**
plate13-A03	CG3705	astray	50	169	6	44	**88.00**
plate13-E01	CG3782	mitochondrial ribosomal protein L28	118	386	14	104	**88.14**
plate20-A03	CG4893	CG4893	118	465	14	104	**88.14**

plate6-C06	CG2118	CG2118	68	180	8	60	**88.24**
plate16-D09	CG4303	Brahma associated protein 60kD	17	59	2	15	**88.24**
plate18-D07	CG4633	mitochondrial alanyl-tRNA synthetase	111	415	13	98	**88.29**
plate12-B03	CG3542	CG3542	387	1253	45	342	**88.37**
plate20-B09	CG4918	Ribosomal protein LP2	215	711	25	190	**88.37**
plate3-G01	CG1634	Neuroglian	121	551	14	107	**88.43**
plate4-A02	CG1683	Adenine nucleotide translocase 2	96	344	11	85	**88.54**
plate18-A03	CG4574	Phospholipase C at 21C	35	161	4	31	**88.57**
plate19-H01	CG4861	LpR1	79	301	9	70	**88.61**
plate3-H02	CG1657	CG1657	54	245	6	48	**88.89**
plate5-A02	CG1873	Elongation factor 1alpha100E	81	268	9	72	**88.89**
plate17-A07	CG4405	junctophilin	135	509	15	120	**88.89**
plate17-H04	CG4559	Imaginal disc growth factor 3	54	220	6	48	**88.89**
plate17-F12	CG4535	FK506-binding protein FKBP59	46	188	5	41	**89.13**
plate3-A04	CG1490	ubiquitin-specific protease 7	74	252	8	66	**89.19**
plate15-A06	CG4058	Neprilysin 4	37	121	4	33	**89.19**
plate13-H02	CG3856	Octopamine receptor in mushroom bodies	56	193	6	50	**89.29**
plate3-D08	CG1575	CG1575	75	273	8	67	**89.33**
plate16-E11	CG4326	mitochondrial ribosomal protein S17	38	179	4	34	**89.47**
plate16-H11	CG4390	CG4390	190	770	20	170	**89.47**
plate6-G05	CG2221	lethal (1) G0289	77	353	8	69	**89.61**
plate5-A06	CG1884	Not1	106	462	11	95	**89.62**
plate16-C06	CG4268	Pitslre	59	276	6	53	**89.83**
plate3-C01	CG1528	gamma-coatomer protein	10	42	1	9	**90.00**
plate18-G08	CG4694	hermaphrodite	41	157	4	37	**90.24**
plate16-H06	CG4382	CG4382	21	113	2	19	**90.48**
plate18-A04	CG4576	CG4576	42	140	4	38	**90.48**
plate3-B02	CG1512	cul-2	106	456	10	96	**90.57**
plate12-A01	CG3515	CG3515	96	335	9	87	**90.63**
plate16-B08	CG4258	dribble	64	200	6	58	**90.63**
plate17-D04	CG4464	Ribosomal protein S19a	151	609	14	137	**90.73**
plate3-F01	CG1615	Open rectifier K[+] channel 1	11	64	1	10	**90.91**
plate11-A12	CG3353	CG3353	33	123	3	30	**90.91**
