# Supplementary material for: Identification of Drosophila Gene Products Required for Phagocytosis of Leishmania donovani
Source: PLoS One. 2012 Dec 13;7(12):e51831. doi: 10.1371/journal.pone.0051831 (PMC3521716; doi:10.1371/journal.pone.0051831)
Supplement: Data S6 — Hits removed from further analysis, as gene products predicted to be involved in proteolysis, translation or RNA processing. (PDF) [file pone.0051831.s006.pdf]

| CG number | Name | No of Cells | Percentage infected | Function |
|---|---|---|---|---|
| **CG3208** | *rhoGAP5A* | 68 | 35.29 | GTPase activating protein |
| **CG1941** | *CG1941* | 306 | 50.98 | Contains diacylglycerol acyltransferase domain |
| **CG1785** | *CG1785* | 329 | 55.02 | Uknown, has p60 tumor suppressor-like domain |
| **CG3494** | *CG3494* | 50 | 56.00 | Phagocytosis, engulfment |
| **CG4162** | *lace* | 84 | 57.14 | Serine palmitoyl transferase subunit 2 |
| **CG2577** | *CG2577* | 430 | 59.77 | Protein amino acid phosphorylation |
| **CG1112** | *alpha-esterase-7* | 217 | 59.91 | Carboxylesterase, type B |
| **CG2076** | *CG2076* | 264 | 60.23 | Homolog of growth hormone inducible transmembrane protein |
| **CG3956** | *snail* | 399 | 60.65 | Transcription factor |
| **CG2086** | *draper* | 72 | 61.11 | Cell adhesion, apoptotic cell receptor |
| **CG31973** | *CG31973* | 524 | 61.45 | Chitin metabolic process |
| **CG1546** | *prolyl-4-hydroxylase-alpha SG2* | 143 | 61.54 | Salivary gland morphogenesis |
| **CG1859** | *spn43Ad* | 168 | 61.90 | Serine-type endopeptidase inhibitor |
| **CG4931** | *sra-1* | 239 | 61.92 | Actin cytoskeleton organization, Rac1 associated |
| **CG4105** | *cytochrome P450-4e3* | 61 | 62.30 | Predicted monooxygenase activity |
| **CG1963** | *pterin-4a-carbinolamine dehydratase* | 308 | 62.34 | Tetrahydrobiopterin biosynthetic process |
| **CG4021** | *CG4021* | 447 | 62.64 | Unknown |
| **CG2179** | *xe7* | 500 | 63.00 | Unknown |
| **CG3036** | *CG3036* | 87 | 63.22 | Posible lysosomal sialic acid transporter |
| **CG1515** | *lethal (1) G0155* | 253 | 63.24 | Vesicle-mediated transport, YKT6 homolog |
| **CG3035** | *carmine* | 445 | 63.37 | Vesicle-mediated transport, AP3µ subunit |
| **CG3380** | *organic anion transporting polypeptide 58Dc* | 52 | 63.46 | Sodium-independent organic anion transmembrane transporter |
| **CG3119** | *CG3119* | 1417 | 63.51 | Unknown |
| **CG3477** | *peroxidase* | 280 | 63.57 | Response to oxidative stress, immunity |
| **CG3085** | *CG3085* | 231 | 63.64 | Microtubule cytoskeleton |
| **CG1071** | *E2F transcription factor 2* | 88 | 63.64 | Transcription factor |
| **CG3480** | *myb-interacting protein 130* | 265 | 63.77 | Centrosome organization; mitotic spindle organization |
| **CG3397** | *CG3397* | 388 | 63.92 | Potassium ion transport |
| **CG1764** | *CG1764* | 374 | 64.44 | Dimethylarginine dimethylaminohydrolase 1 (DDAH1) |
| **CG3156** | *CG3156* | 377 | 64.46 | Predicted ABCB10 (MDR/TAP) family mitochondrial transporter |
| **CG2292** | *CG2292* | 127 | 64.57 | GPI anchor biosynthetic process |
| **CG2201** | *CG2201* | 302 | 64.57 | Choline kinaseα |
| **CG2381** | *syt7* | 195 | 64.62 | Neurotransmitter secretion, vesicle-mediated transport; synaptic vesicle exocytosis |
| **CG1472** | *sec24* | 145 | 64.83 | ER to Golgi vesicle-mediated transport |

**Supplementary Table 3**

Curated list of genes that resulted in decreased infection rates post RNAi.

Predicted functions are shown where data are available.
