# Supplementary material for: Identification of Drosophila Gene Products Required for Phagocytosis of Leishmania donovani
Source: PLoS One. 2012 Dec 13;7(12):e51831. doi: 10.1371/journal.pone.0051831 (PMC3521716; doi:10.1371/journal.pone.0051831)
Supplement: Data S7 — Targets removed from further analysis as their introduction led to cell death. (PDF) [file pone.0051831.s007.pdf]

Target	Original probe generated?	Validation probe generated?	Validation probe Amplicon ID
**GFP**	Yes (control)	-	
**CG1764**	Yes	Yes	DRSC40329
**RhoGap5a**	No	Yes	DRSC38990
**SCAR**	No	Yes	DRSC03426
**Carmine**	Yes	Yes	BKN27773
**Rab5**	No	Yes	DRSC23710
**Lace**	Yes	Yes	DRSC32141
**Sra1**	No	Yes	DRSC31675
**Syt 7**	No	Yes	DRSC17130
**Arpc3a**	No	Yes	DRSC27915
**Sec24**	Yes	Yes	DRSC25940
**CG2076**	Yes	Yes	DRSC35041
**ABCB10**	Yes	Yes	E-RNAi
**Draper**	Yes	Yes	DRSC08143
**CG1515**	Yes	Yes	DRSC31634
**Neuroglian**	Yes	Yes	DRSC27797
**Syntaxin V**	No	Yes	DRSC03432

Supplementary Data 7: List of probes used for validation screening
