# Supplementary material for: Identification of Drosophila Gene Products Required for Phagocytosis of Leishmania donovani
Source: PLoS One. 2012 Dec 13;7(12):e51831. doi: 10.1371/journal.pone.0051831 (PMC3521716; doi:10.1371/journal.pone.0051831)
Supplement: Data S8 — Hits removed from further analysis as their dsRNAs contained multiple ≥19nt sequences with identity to other gene products and thus are predicted to generate off target effects. (DOCX) [file pone.0051831.s008.docx]

| CG number | Gene Name | No of cells | Percentage infected | Function | No of OTEs |
| --- | --- | --- | --- | --- | --- |
| CG2577 | *CG2577* | 430 | 60 | Protein amino acid phosphorylation | **5** |
| CG3367 | *CG42340* | 234 | 60 | Potassium ion transport | **26** |
| CG2028 | *casein kinase I alpha* | 60 | 62 | DNA repair, protein amino acid phosphorylation | **6** |
| CG1179 | *lysozyme B* | 303 | 63 | Antimicrobial humoral response | **5** |
| CG3827 | *scute* | 64 | 64 | Bristle morphogenesis, nervous system development, negative regulation of apoptosis | **4** |
| CG2256 | *CG2256* | 253 | 65 | Unknown | **88** |
| CG1829 | *cyp6v1* | 295 | 65 | Cytochrome P450 | **26** |
| CG2252 | *female sterile (1) homeotic* | 259 | 66 | Regulation of transcription from RNA polymerase II promoter | **856** |
| CG1775 | *medea* | 405 | 66 | Transforming growth factor beta receptor signalling pathway, regulation of transcription | **10** |

**Supplementary Data 8:** Hits removed from further analysis as their dsRNA contained multiple ≥19nt sequences with identity to other gene products and thus are predicted to generate off target effects.
