# Supplementary material for: Identification of Drosophila Gene Products Required for Phagocytosis of Leishmania donovani
Source: PLoS One. 2012 Dec 13;7(12):e51831. doi: 10.1371/journal.pone.0051831 (PMC3521716; doi:10.1371/journal.pone.0051831)
Supplement: Data S9 — Curated list of hits that decreased infection rates. (DOCX) [file pone.0051831.s009.docx]

| CG number | Name | No of Cells | Percentage infected | Function |
| --- | --- | --- | --- | --- |
| CG3208 | *rhoGAP5A* | 68 | 35.29 | GTPase activating protein |
| CG1941 | *CG1941* | 306 | 50.98 | Contains diacylglycerol acyltransferase domain |
| CG1785 | *CG1785* | 329 | 55.02 | Uknown, has p60 tumor suppressor-like domain |
| CG3494 | *CG3494* | 50 | 56.00 | Phagocytosis, engulfment |
| CG4162 | *lace* | 84 | 57.14 | Serine palmitoyl transferase subunit 2 |
| CG2577 | *CG2577* | 430 | 59.77 | Protein amino acid phosphorylation |
| CG1112 | *alpha-esterase-7* | 217 | 59.91 | Carboxylesterase, type B |
| CG2076 | *CG2076* | 264 | 60.23 | Homolog of growth hormone inducible transmembrane protein |
| CG3956 | *snail* | 399 | 60.65 | Transcription factor |
| CG2086 | *draper* | 72 | 61.11 | Cell adhesion, apoptotic cell receptor |
| CG31973 | *CG31973* | 524 | 61.45 | Chitin metabolic process |
| CG1546 | *prolyl-4-hydroxylase-alpha SG2* | 143 | 61.54 | Salivary gland morphogenesis |
| CG1859 | *spn43Ad* | 168 | 61.90 | Serine-type endopeptidase inhibitor |
| CG4931 | *sra-1* | 239 | 61.92 | Actin cytoskeleton organization, Rac1 associated |
| CG4105 | *cytochrome P450-4e3* | 61 | 62.30 | Predicted monooxygenase activity |
| CG1963 | *pterin-4a-carbinolamine dehydratase* | 308 | 62.34 | Tetrahydrobiopterin biosynthetic process |
| CG4021 | *CG4021* | 447 | 62.64 | Unknown |
| CG2179 | *xe7* | 500 | 63.00 | Unknown |
| CG3036 | *CG3036* | 87 | 63.22 | Posible lysosomal sialic acid transporter |
| CG1515 | *lethal (1) G0155* | 253 | 63.24 | Vesicle-mediated transport, YKT6 homolog |
| CG3035 | *carmine* | 445 | 63.37 | Vesicle-mediated transport, AP3µ subunit |
| CG3380 | *organic anion transporting polypeptide 58Dc* | 52 | 63.46 | Sodium-independent organic anion transmembrane transporter |
| CG3119 | *CG3119* | 1417 | 63.51 | Unknown |
| CG3477 | *peroxidase* | 280 | 63.57 | Response to oxidative stress, immunity |
| CG3085 | *CG3085* | 231 | 63.64 | Microtubule cytoskeleton |
| CG1071 | *E2F transcription factor 2* | 88 | 63.64 | Transcription factor |
| CG3480 | *myb-interacting protein 130* | 265 | 63.77 | Centrosome organization; mitotic spindle organization |
| CG3397 | *CG3397* | 388 | 63.92 | Potassium ion transport |
| CG1764 | *CG1764* | 374 | 64.44 | Dimethylarginine dimethylaminohydrolase 1 (DDAH1) |
| CG3156 | *CG3156* | 377 | 64.46 | Predicted ABCB10 (MDR/TAP) family mitochondrial transporter |
| CG2292 | *CG2292* | 127 | 64.57 | GPI anchor biosynthetic process |
| CG2201 | *CG2201* | 302 | 64.57 | Choline kinaseα |
| CG2381 | *syt7* | 195 | 64.62 | Neurotransmitter secretion, vesicle-mediated transport; synaptic vesicle exocytosis |
| CG1472 | *sec24* | 145 | 64.83 | ER to Golgi vesicle-mediated transport |

**Supplementary Data 9:** Curated list of genes that resulted in decreased infection rates post RNAi. Predicted functions are shown where data are available.
