# Supplementary material for: Identification of Drosophila Gene Products Required for Phagocytosis of Leishmania donovani
Source: PLoS One. 2012 Dec 13;7(12):e51831. doi: 10.1371/journal.pone.0051831 (PMC3521716; doi:10.1371/journal.pone.0051831)
Supplement: Data S10 — List of dsRNA probes used for validation screening. (DOCX) [file pone.0051831.s010.docx]

| **Target** | **Original probe**  **re-synthesised?** | **Validation probe**  **synthesised?** | **Validation probe Amplicon ID** |
| --- | --- | --- | --- |
| **GFP** | Yes (control) | - |  |
| **CG1764** | Yes | Yes | DRSC40329 |
| **RhoGap5a** | No | Yes | DRSC38990 |
| **SCAR** | No | Yes | DRSC03426 |
| **Carmine** | Yes | Yes | BKN27773 |
| **Rab5** | No | Yes | DRSC23710 |
| **Lace** | Yes | Yes | DRSC32141 |
| **Sra1** | No | Yes | DRSC31675 |
| **Syt 7** | No | Yes | DRSC17130 |
| **Arpc3a** | No | Yes | DRSC27915 |
| **Sec24** | Yes | Yes | DRSC25940 |
| **CG2076** | Yes | Yes | DRSC35041 |
| **ABCB10** | Yes | Yes | E-RNAi |
| **Draper** | Yes | Yes | DRSC08143 |
| **CG1515** | Yes | Yes | DRSC31634 |
| **Neuroglian** | Yes | Yes | DRSC27797 |
| **Syntaxin V** | No | Yes | DRSC03432 |

**Supplementary Data 10**: List of probes used for validation screening
